# Palmitoylation-driven PHF2 ubiquitination remodels lipid metabolism through the SREBP1c axis in hepatocellular carcinoma

**DOI:** 10.1038/s41467-023-42170-0

**Published:** 2023-10-12

**Authors:** Do-Won Jeong, Jong-Wan Park, Kyeong Seog Kim, Jiyoung Kim, June Huh, Jieun Seo, Ye Lee Kim, Joo-Youn Cho, Kwang-Woong Lee, Junji Fukuda, Yang-Sook Chun

**Affiliations:** 1 Department of Biomedical Sciences, Seoul National University College of Medicine, Seoul, 03080 Korea; 2 Department of Physiology, Seoul National University College of Medicine, Seoul, 03080 Korea; 3 Ischemic/Hypoxic Disease Institute, Seoul National University College of Medicine, Seoul, 03080 Korea; 4 Department of Clinical Pharmacology and Therapeutics, Seoul National University College of Medicine and Hospital, Seoul, 03080 Korea; 5 Department of Chemical and Biological Engineering, Korea University, Seoul, 02841 Korea; 6 Faculty of Engineering, Yokohama National University, Yokohama, 240-8501 Japan; 7 Department of Surgery, Seoul National University College of Medicine, Seoul, 03080 Korea

**Keywords:** Ubiquitin ligases, Mechanisms of disease, Cancer metabolism, Fat metabolism, Ubiquitylation

## Abstract

Palmitic acid (PA) is the most common fatty acid in humans and mediates palmitoylation through its conversion into palmitoyl coenzyme A. Although palmitoylation affects many proteins, its pathophysiological functions are only partially understood. Here we demonstrate that PA acts as a molecular checkpoint of lipid reprogramming in HepG2 and Hep3B cells. The zinc finger DHHC-type palmitoyltransferase 23 (ZDHHC23) mediates the palmitoylation of plant homeodomain finger protein 2 (PHF2), subsequently enhancing ubiquitin-dependent degradation of PHF2. This study also reveals that PHF2 functions as a tumor suppressor by acting as an E3 ubiquitin ligase of sterol regulatory element-binding protein 1c (SREBP1c), a master transcription factor of lipogenesis. PHF2 directly destabilizes SREBP1c and reduces SREBP1c-dependent lipogenesis. Notably, SREBP1c increases free fatty acids in hepatocellular carcinoma (HCC) cells, and the consequent PA induction triggers the PHF2/SREBP1c axis. Since PA seems central to activating this axis, we suggest that levels of dietary PA should be carefully monitored in patients with HCC.

## Abstract (web summary)

Palmitoylation of proteins can have pathophysiological implications. Here, the authors show that palmitoylation enhances the proteasomal degradation of the histone demethylase PHF2, leading to increased lipogenesis and cell proliferation in an SREBP1c dependent manner and further show that PHF2 acts as an E3 ligase of SREBP1c, suppressing the growth of liver cancer cells.

## Introduction

Plant homeodomain finger protein 2 (PHF2) demethylates lysine 9 in histone 3 (H3K9) and relieves gene silencing as an epigenetic regulator via its Jumonji C domain-dependent demethylase activity^1^. PHF2 is located in the human chromosomal region 9q22.3^2^ and is often absent in several cancers, including bladder, esophageal, head and neck, and prostate cancers^2,3^. Moreover, several studies have revealed that PHF2 acts as a tumor suppressor. PHF2 is an epigenetic coactivator of p53 in colon cancer^4^, and miR-221-mediated PHF2 downregulation is linked to higher cell migration and poor liver cancer prognosis^5^. PHF2 is downregulated in breast cancer, and overexpression of PHF2 has been shown to inhibit the proliferation of breast cancer cells^6^. PHF2 also contains plant homeodomain (PHD), structurally similar to RING E3 ubiquitin ligase^7^. Although PHD-containing proteins exhibit E3 ligase activity and mediate target-protein ubiquitination^8,9^, the function of PHF2 as an E3 ligase has yet to be elucidated.

High-fat diet (HFD) gives tumor cells a survival advantage. High amounts of fatty acids (FAs) in an HFD produce energy in cells and are needed for an intracellular signaling molecule involved in the development of cancers^10,11^. Several studies have indicated that the impact of dietary fat depends on individual FA levels. For example, saturated and n-6 polyunsaturated FAs boost the metastatic potential and increase cancer risk^12,13^, whereas n-3 polyunsaturated FAs are more likely to prevent carcinogenesis^14,15^. However, the effect of monounsaturated FAs in cancer progression is tissue-dependent and still controversial^16,17^.

Hepatocellular carcinoma (HCC) is the third leading cause of cancer-related death, with a 5-year survival rate of <9%^18^. HCC incidence is closely related to obesity and nonalcoholic fatty liver disease^19^, and the FAs profile is altered in HCC patients^20^. The levels of polyunsaturated FAs, including linoleic acid, are inversely associated with HCC risk^21^. In contrast, aggressive HCC is associated with increased levels of oleic acid and palmitic acid (PA)^22,23^. PA is the most abundant FA in the liver^24^. Under normal conditions, the liver regulates PA concentration by mediating its desaturation into palmitoleic acid or elongation into stearic and oleic acid^24^. However, PA balance is disrupted under pathophysiological conditions and aberrant accumulation of PA results in aggravation of cancer progression^24^. Indeed, PA treatment increases cell proliferation and tumor sphere formation in HCC cells^23^. Dietary PA decreases fat oxidation and increases energy storage^25,26^, and PA is less prone to lipid peroxidation than polyunsaturated FAs^27^. In addition, the increased uptake of PA by cancer cells from the tumor microenvironment leads to impaired CD8 + T cell infiltration^28^. Even though PA provides cancer cells with a better environment for survival and may act as a valuable molecular checkpoint for HCC, the underlying mechanisms of how PA contributes to HCC progression are yet to be elucidated.

Many proteins undergo irreversible lipidation events, including N‐myristoylation, S‐farnesylation, O‐palmitoylation, and N‐palmitoylation^29^. By contrast, protein S‐palmitoylation (hereafter simply palmitoylation) is the thioesterification of palmitate to an internal cysteine residue of proteins catalyzed by ZDHHCs^30^. It alters the stability, function, and localization of hundreds of proteins in the cell^31^. Palmitoylation is responsible for the function of both oncogenes (e.g., EGFR) and tumor suppressors (e.g., SCRIB and melanocortin 1 receptor)^32^. Thus, understanding how protein palmitoylation regulates the function of individual proteins in normal and cancer cells is an important driver of current cancer research.

In the liver, lipid homeostasis is regulated via a regulatory feedback system by sterol regulatory element-binding protein 1c (SREBP1c)^33^. SREBP1c stimulates multiple lipogenic genes resulting in quantitative changes of FAs such as stearic and oleic acid^34,35^. Notably, SREBP1c may be a critical link between oncogenic signaling and lipid metabolism in various types of cancer. Increased SREBP1 expression has been reported in breast, gastric, skin, and thyroid cancers^36–39^. In addition, SREBP1 promotes the malignant characteristics of colon, gastric, kidney, and liver cancer^39–42^. Moreover, inhibition of the SREBP pathway reduces intracellular PA in liver cancer cells^34^ and HCC progression by repressing tumor-promoting cytokines such as interleukin (IL) 6 and tumor necrosis factor alpha^43^. Hence, it is likely that cancer cells govern SREBP1c to maintain their proliferation. Despite the importance of SREBP1c, most studies have focused on regulating its transcriptional activity rather than on its post-translational modification.

Here, we report that PA rewires lipid metabolism, subsequently regulating HCC progression in a PHF2/SREBP1c axis-dependent manner. Following ZDHHC23-mediated PHF2 palmitoylation, PA reduces PHF2 protein level through proteasomal degradation. We found that PA-induced lipid accumulation is due to palmitoylation-driven PHF2 protein degradation and SREBP1c induction. This study also reveals that PHF2 acts as an E3 ubiquitin ligase for SREBP1c. Because PA acts as a molecular checkpoint for the linkage between the PHF2/SREBP1c axis and HCC progression, we suggest that the PA intake of patients with HCC should be carefully monitored.

## Results

### PHF2 is palmitoylated at cysteine 23

**Fig. 1:**
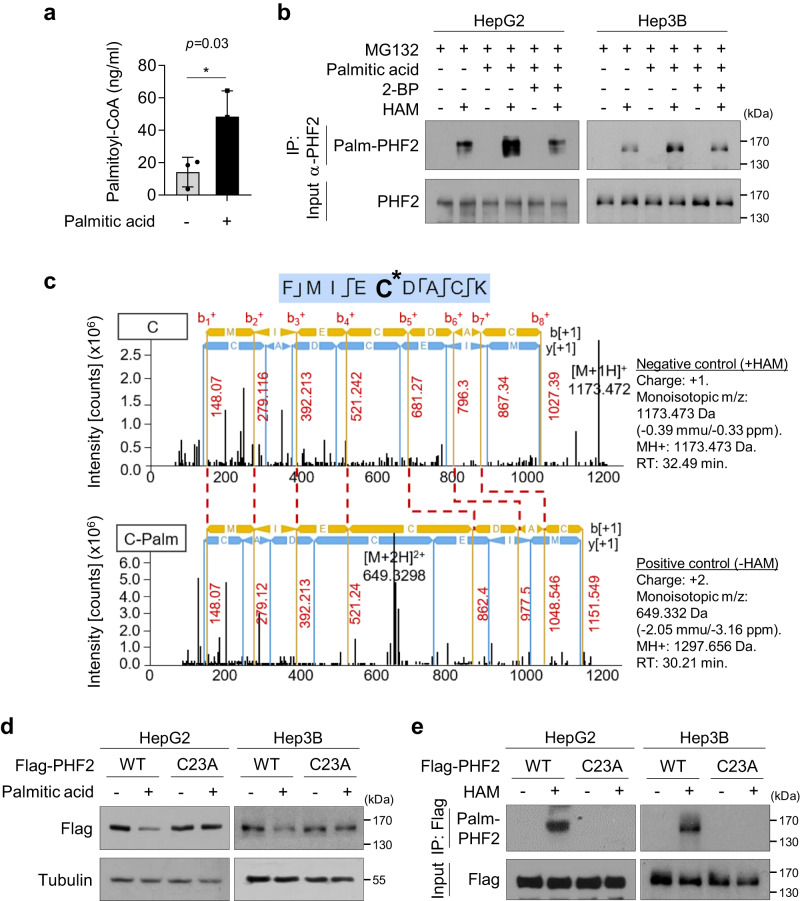
PHF2 is palmitoylated at cysteine 23. **a** Levels of palmitoyl-coenzyme A (palmitoyl-CoA) were quantified using liquid chromatography-mass spectrometry (LC-MS) in HepG2 cells treated by palmitic acid (PA) treatment for 24 h. Mean ± SD (*n* = 3 independent samples); *P < 0.05. Statistical analyses were based on a two-tailed unpaired *t* test. The exact p-values are presented in Supplementary Data 2. **b** Hepatocellular carcinoma (HCC) cells were pre-treated with 2-bromopalmitate (2-BP, 50 µM) or dimethyl sulfoxide (DMSO) for 24 h and incubated with PA for 24 h, followed by MG132 (10 µM) for 8 h. Immunoprecipitation was performed using an anti-PHF2 antibody. The precipitated proteins were subjected to an acyl-biotin exchange (ABE) assay using hydroxylamine (HAM) treatment to remove PA from palmitoylated cysteine residues. Free cysteines were labeled with BMCC-Biotin. Finally, palmitoylated proteins were detected using streptavidin-HRP. *n* = 3 independent experiments. IP: immunoprecipitation; Palm-PHF2: palmitoylated PHF2. **c** The palmitoylation site within PHF2 was identified using LC-MS analysis. The y and b fragments detected are as indicated in the sequence. The palmitoylation of cysteine 23 residue (C23) in PHF2 was identified based on the shift of peptide peaks. **d** After transfection with wild-type (WT) PHF2 or C23A-mutated PHF2 plasmids, HCC cells lysates were subjected to western blotting. *n* = 3 independent experiments. **e** HCC cells were transfected with Flag-PHF2-WT or Flag-PHF2-C23A mutant. The expressed proteins were immunoprecipitated with Flag affinity beads and subjected to ABE assay. The palmitoylated proteins were detected using western blotting. *n* = 3 independent experiments. Source data are provided as a Source Data file.

Reprogramming of FA metabolism is a hallmark of cancer cells; therefore, we first examined whether excessive changes in FAs flux could regulate PHF2 levels in HCC cells. HepG2 and Hep3B cells were treated with various FAs, of which PA reduced PHF2 levels (Supplementary Fig. 1a); PA also reduced PHF2 expression in other liver cancer cell lines (Supplementary Fig. 1b). Because excess PA may be metabolized into palmitoyl-coenzyme A (CoA) and then transferred to the cysteine residue of target proteins by ZDHHC-mediated S-palmitoylation, we checked whether PA treatment increased palmitoyl-CoA and PHF2 palmitoylation in HCC cells. Data from liquid chromatography-mass spectrometry (LC-MS) analysis showed an increased level of palmitoyl-CoA after PA treatment in HepG2 cells (Fig. 1a). Palmitoylation of endogenous PHF2 was evaluated using acyl-biotin-exchange assay and was enhanced by PA treatment in HCC cells (Fig. 1b). Moreover, the palmitoylation inhibitor, 2-bromopalmitate (2-BP, 50 µM), abolished PA-induced PHF2 palmitoylation comparable to the control level (Fig. 1b). Next, we generated four segments of PHF2. We found that only the PHD motif was palmitoylated, and its protein expression was reduced by PA (Supplementary Fig. 1c, d). An LC-MS analysis showed that cysteine 23 (C23) at PHF2 was palmitoylated (Fig. 1c and Supplementary Fig. 1e). We generated a cysteine to alanine PHF2 mutant (PHF2-C23A) and confirmed that the substitution did not change the structure of the PHD domain using computational simulation Dynamut^44^ (Supplementary Fig. 1f). The PHF2-C23A mutant failed to undergo reduction and palmitoylation by PA treatment (Fig. 1d, e). Collectively, these data indicate that PHF2 is palmitoylated at the C23 residue.

### ZDHHC23-mediated PHF2 palmitoylation at cysteine 23 enhances its ubiquitination

**Fig. 2:**
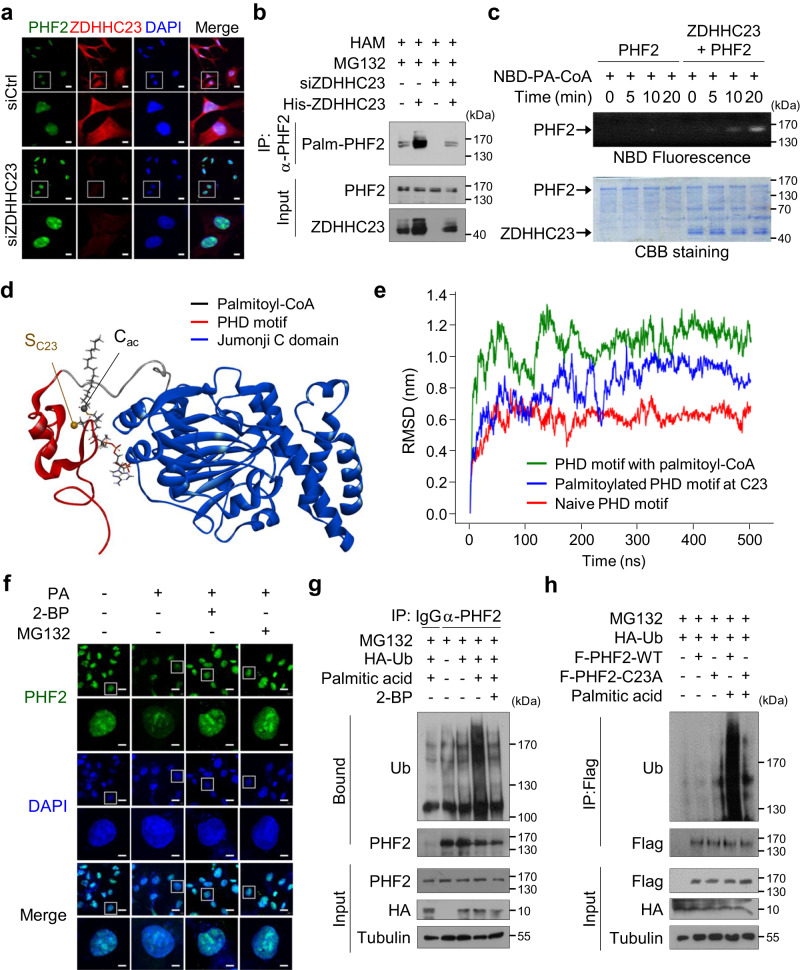
ZDHHC23-mediated PHF2 palmitoylation at cysteine 23 enhances its ubiquitination. **a** Hep3B cells were transfected with siZDHHC23 and immunofluorescence analysis was performed using indicated antibodies. Scale bar = 20 (low magnification) and 6 µm (high magnification). **b** HepG2 cells were transfected with siZDHHC23 or His-ZDHHC23, and treated with MG132. After immunoprecipitation with an anti-PHF2 antibody, the palmitoylated PHF2 was detected using ABE assay. *n* = 3 independent experiments. **c** An in vitro palmitoylation assay was performed using purified PHF2 and ZDHHC23 proteins. PHF2 palmitoylation was detected using a fluorescence-labeled palmitoyl-CoA analog. The proteins used in the assay were stained using Coomassie Brilliant Blue staining. *n* = 3 independent experiments. NBD-PA-CoA: (N-[(7-nitro-2-1,3-benzoxadiazol-4-yl)-methyl] amino) palmitoyl-coenzyme A. **d** 3D structure of PHF2 when the palmitoyl-CoA is in close proximity to C23 of the PHD domain (*d* = 5 Å). Red and blue ribbons represent the plant homeodomain (PHD) and Jumonji C domain, respectively, and sticks represent palmitoyl-CoA. **e** The time evolution of root mean square deviation (RMSD) for the PHD motif in the presence of palmitoyl-CoA (*d* = 5 Å), the PHD motif in the absence of palmitoyl-CoA, and the palmitoylated PHD domain in target to those of native particulate methane monooxygenase (pMMO) as a reference structure. **f** Hep3B cells were pre-treated with 2-BP and incubated with PA, followed by treatment with MG132. The level of PHF2 protein was determined using immunofluorescence. Scale bar = 20 (low magnification) and 5 µm (high magnification). **g** HepG2 cells were transfected with the indicated plasmids and treated with 2-BP and PA. After treatment of MG132 for 8 h, cells were immunoprecipitated using an anti-PHF2 antibody and subjected to immunoblotting. *n* = 3 independent experiments. **h** Flag-PHF2-WT or Flag-PHF2-C23A transfected cells were treated with PA and MG132. Immunoprecipitation was performed using Flag affinity beads. *n* = 3 independent experiments. F: Flag. Source data are provided as a Source Data file.

To identify the enzymes involved in PHF2 palmitoylation, we knocked down 23 ZDHHCs using siRNAs (Supplementary Fig. 2a). siZDHHC4, siZDHHC5, siZDHHC11, siZDHHC17, and siZDHHC23 significantly inhibited PHF2 palmitoylation (Supplementary Fig. 2b), and ZDHHC23 was identified as the predominant enzyme for endogenous PHF2 palmitoylation in HepG2 cells (Supplementary Fig. 2c). Moreover, siZDHHC23 significantly blocked endogenous PHF2 palmitoylation even in the presence of high levels of PA (Supplementary Fig. 2d). Among the mRNA expressions of endogenous *ZDHHCs* in HepG2 and Hep3B cells, *ZDHHC23* was highly expressed in both cell lines compared with other *ZDHHCs* family members (Supplementary Fig. 2e). Most ZDHHCs are known to be localized in ER/Plasma membrane^45^. However, immunohistochemical analysis showed that ZDHHC23 was localized both in the cytoplasm and nucleus of human liver tissue (Supplementary Fig. 2f). We also confirmed that PHF2 and ZDHHC23 were co-localized in the nucleus using immunofluorescence and nuclear extraction assays in HCC cells (Fig. 2a and Supplementary Fig. 2g). Consistent with the reported interactions between palmitoyltransferases and their substrates^46,47^, ZDHHC23 bound directly to wild-type (WT-PHF2) but not to the PHF2–C23A mutant (Supplementary Fig. 2h). Furthermore, siZDHHC23 increased endogenous PHF2 protein level but the ectopic expression of ZDHHC23 reversed the PHF2 expression back to the control level (Supplementary Fig. 2i), in addition to increasing PHF2 palmitoylation in the siZDHHC23-transfected cells (Fig. 2b). Because some proteins undergo autopalmitoylation^48^, we tried to examine the possibility of PHF2 autopalmitoylation using an in vitro palmitoylation assay. ZDHHC23 exhibited palmitoyltransferase activity, whereas PHF2 did not show autoacylation activity (Fig. 2c).

Next, to explore the molecular mechanism responsible for the reduction of PHF2 induced by PA, we first analyzed the structural alteration of PHF2 in the presence of palmitoyl-CoA. We used an atomistic molecular dynamics (MD) simulation to study the position of palmitoyl-CoA in PHF2. To do this, we enforced a distance restraint between the sulfur atom of C23 (S_C23_) and the acyl carbon (C_ac_) of palmitoyl-CoA. According to the potential of mean force (PMF) profile, which shows the change in free energy, palmitoyl-CoA and the PHD domain formed a complex when the distance between the S_C23_-C_ac_ distance was approximately 5 Å following Jarzynski’s theorem^49^ (Fig. 2d and Supplementary Fig. 3a). It can be considered that the minimum at *d* ≅ 5 Å is a stationary state for palmitoyl-CoA, where it is in its closest position to C23 before the palmitoylation takes place. Next, the structural change of the PHD domain in the presence of palmitoyl-CoA was investigated by tracking the time evolution of root mean square deviation (RMSD) and Define Secondary Structure of Proteins (DSSP) analysis^50^ during the distance restraint-MD simulation at *d* = 5 Å. The RMSD of the PHD domain with palmitoyl-CoA fluctuated around 11 Å, which is approximately two times larger than that for the PHD domain in the absence of palmitoyl-CoA (Fig. 2e). A similar but slightly smaller RMSD (≅9 Å) was observed for the palmitoylated PHD motif. The smaller RMSD possibly reflected the removal of the bulky CoA group after the palmitoylation of C23 (Fig. 2e). DSSP analysis also revealed that the presence of palmitoyl-CoA resulted in notable alterations in the secondary structure of the PHD motif. In particular, the β-sheets in the region encompassing amino acids 19-31 were disrupted (Supplementary Fig. 3b). These results indicated that the PHD domain underwent substantial structural alterations when palmitoyl-CoA was in proximity to C23. Because palmitoylation modulates protein stability^31^, we next checked palmitoylation-mediated PHF2 degradation using proteasomal inhibitors (MG132 and bortezomib) and autophagy inhibitors (hydroxychloroquine and NH_4_Cl). PA-induced PHF2 reduction was ameliorated by 2-BP or proteasomal inhibitors (Supplementary Fig. 3c), implying the interplay between PHF2 palmitoylation and proteasomal degradation but not lysosomal degradation. Immunofluorescence assay supported the observation that PA-mediated PHF2 reduction was recovered by 2-BP and MG132 (Fig. 2f and Supplementary Fig. 3d). However, *PHF2* mRNA levels in HCC cells were not affected by PA treatment (Supplementary Fig. 3e). Finally, we found that PA enhanced PHF2 ubiquitination, which was reversed by 2-BP (Fig. 2g). WT-PHF2 underwent dramatically enhanced ubiquitination by PA treatment. However, the PHF2-C23A mutant was nearly negative even after PA treatment (Fig. 2h), indicating that PHF2 palmitoylation at C23 is essential for PHF2 ubiquitination. Moreover, PA treatment highly enhanced PHF2 degradation rates by 5.6-fold (Supplementary Fig. 3f, left panel), and PHF2 protein was stabilized in siZDHHC23 expressing HepG2 cells. In addition, ZDHHC23 knock-down HepG2 cells failed to palmitoylate PHF2, and PHF2 became stable even in a PA-enriched environment (Supplementary Fig. 3f, right panel). Taken together, PHF2 palmitoylation at C23 enhances its ubiquitination-dependent degradation.

### PHF2 is a determinant of PA-induced lipogenesis in HCC cells

**Fig. 3:**
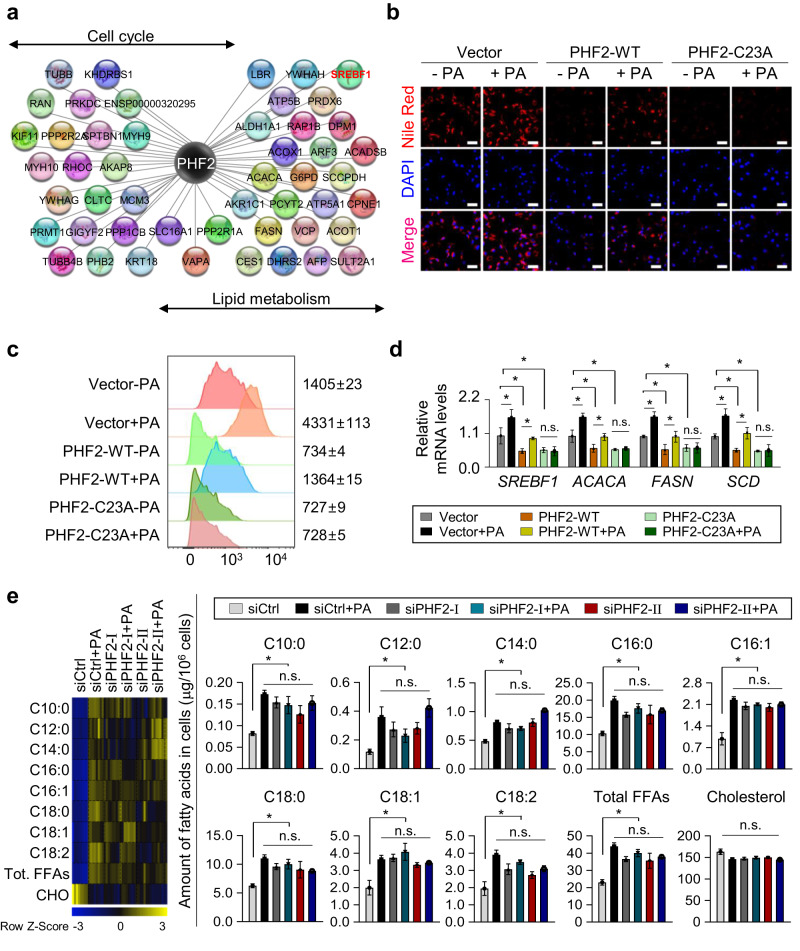
PHF2 is a determinant of PA-induced lipogenesis in HCC cells. **a** HepG2 cells were transfected with Flag/SBP-PHF2 plasmid. The proteins purified by Flag or SA affinity beads were subjected to LC-MS and then co-expressed proteins were analyzed. Systemic networks generated by Cytoscape presented the functions of PHF2-interacting proteins. Hep3B cells were transfected with the indicated plasmids and treated with or without PA. **b** Cells were fixed with formaldehyde, stained with Nile Red and DAPI, and visualized using a fluorescence microscope. Scale bar = 60 µm. *n* = 3 independent experiments. **c** FACS analysis of Nile Red stained cells. Numbers indicate the mean fluorescence intensity. **d** Total RNAs were isolated from the transfected Hep3B cells with the indicated plasmids. The expression of lipogenesis-related genes was quantified by RT-qPCR relative to 18S RNA. Mean ± SD (*n* = 3 independent experiments); *P < 0.05. Statistical analyses were based on a two-tailed unpaired t-test. **e** siControl or siPHF2s transfected HepG2 cells were treated with or without PA. Each free fatty acid (FFA) of cells (µg/10^6^ cells) was analyzed by GC-TOF/MS. The color scale bar represents relative expression values ranging from low (blue) to high (yellow). Mean ± SD (*n* = 8 independent samples); *P < 0.05 by a two-tailed unpaired *t* test. Tot. total; CHO cholesterol. The exact *p* values are provided in Supplementary Data 2.

To explore the role of PHF2 in HCC progression, we analyzed PHF2-interacting proteins in HCC cells using LC-MS combined with immunoprecipitation (IP). In total, 295 PHF2-interacting proteins were identified and annotated using functional enrichment analysis. The results revealed that PHF2 was closely associated with lipid metabolism and cell cycle (Fig. 3a and Supplementary Fig. 4a). Thus, we first evaluated whether PA-induced PHF2 loss could affect lipogenesis in HCC cells. Nile Red staining showed lipid accumulation was similarly enhanced by PA treatment and PHF2 knockdown in HCC cells. Notably, PA treatment did not improve lipogenesis in PHF2 knock-down HCC cells (Supplementary Fig. 4b), indicating that PHF2 is a determinant of PA-induced lipogenesis in HCC cells. In contrast, Nile Red staining and flow cytometric analysis of Nile Red stained cells showed that the WT-PHF2 and PHF2-C23A mutant overexpression markedly reduced lipid accumulation. Moreover, PA increased lipid accumulation in cells expressing WT-PHF2 but failed to restore lipid accumulation in HCC cells expressing the PHF2-C23A mutant (Fig. 3b, c, and Supplementary Fig. 4c, d). Based on these findings, it appears that the accumulation of lipids caused by PA may be linked to the palmitoylated PHF2, which leads to a decrease in PHF2 levels. Following this trend, mRNA levels of lipogenic genes such as FA synthase (*FASN*) and stearoyl-CoA desaturase (*SCD*) showed similar changes (Fig. 3d and Supplementary Fig. 4e,f). To quantify captured free FAs (FFAs) in HepG2 cells, we performed gas chromatography–time-of-flight–mass spectrometry (GC-TOF/MS). Although various FFAs, including PA (C16:0), were upregulated in the siPHF2s and PA-treated groups, those transfected with siPHF2 showed no further induction by PA treatment (Fig. 3e). Collectively, these results suggest that PHF2 is an essential mediator of PA-induced lipogenesis in HCC cells.

### SREBP1c is essential for PHF2 loss-induced lipogenesis and cell proliferation

Increased de novo lipid synthesis gives cancer cells a survival advantage and is closely associated with poor prognosis. Because our results showed that PHF2 negatively regulated lipogenesis in HCC cells, we wondered whether PHF2 has a tumor-suppressive role in HCC and what the mediator of PHF2 is. Among PHF2-interacting proteins (Fig. 3a), SREBP1c is a crucial transcription factor involved in lipid metabolism in the liver. Therefore, we analyzed the levels of *PHF2* and SREBP1c downstream genes using the gene expression data (transcriptome; gene expression, and non-coding RNA profiling by array) of human HCC tissues from a public dataset (GSE54238). Notably, *PHF2* mRNA levels were lower, and the mRNA levels of *SREBF1*, acetyl-CoA carboxylase, and *FASN*, which are the SREBP1c downstream genes, were higher in HCC tissues compared with those in the adjacent normal tissues (Supplementary Fig. 5a). In the dataset, *PHF2* mRNA levels were negatively correlated with the expression of cell cycle-related genes, HCC markers, and SREBP1c downstream genes (Supplementary Fig. 5b).

**Fig. 4:**
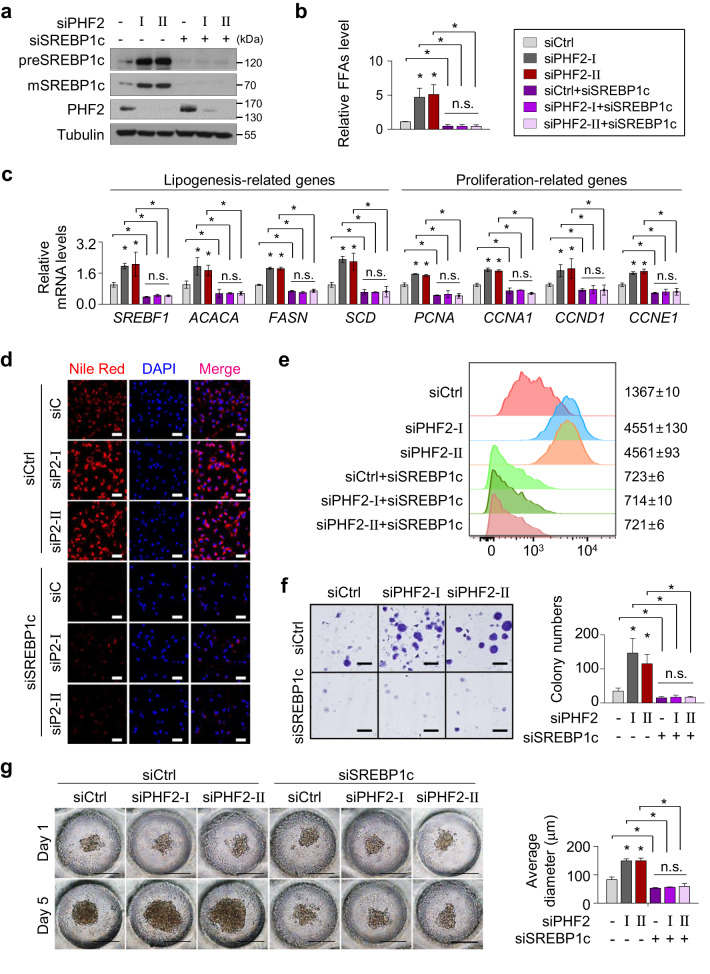
SREBP1c is essential for PHF2 loss-induced lipogenesis and cell proliferation. **a**–**g** Hep3B cells were transfected with the indicated siRNAs. **a** Proteins were analyzed using western blotting. *n* = 3 independent experiments. Source data are provided as a Source Data file. **b** Total FFA levels of cells were measured; mean ± SD (*n* = 3 independent samples). *P < 0.05. **c** The expression of lipogenesis- and proliferation-related genes were analyzed by RT-qPCR. mRNA levels were quantified relative to 18S RNA levels; mean ± SD (*n* = 3 independent experiments). *P < 0.05. **d** Representative images of Nile Red stained cells. Hep3B cells stained by Nile Red and DAPI were visualized by fluorescence microscopy. *n* = 3 independent experiments. Scale bar = 60 µm. siP2: siPHF2. **e** FACS analysis of Nile Red stained cells. Numbers indicate the mean fluorescence intensity. **f** The colony formation assay of Hep3B cells. Hep3B cells (1 × 10^4^ cells/well) were seeded in six-well plates. Images were acquired after fixation with methanol and staining with 0.5% crystal violet. Scale bar = 4 mm. Colony numbers were counted; mean ± SD (*n* = 3 independent samples); *P < 0.05. **g** Representative light micrographs of spheroid formation of Hep3B cells cultured on oxygen-permeable chips for 1 and 5 days after seeding. Scale bar = 200 µm. The average spheroid diameter on Oxy chips was quantified using ImageJ. Mean ± SD (*n* = 3 independent experiments); *P < 0.05. For the analyses in (**b, c, f, g**), an unpaired two-tailed Student′s *t* test was conducted. The exact *p* values are shown in Supplementary Data 2.

Next, we investigated whether PHF2 could regulate lipogenesis in an SREBP1c-dependent manner in HCC cells. PHF2 knockdown using siRNAs increased SREBP1c protein levels and total FFAs production in Hep3B cells (Fig. 4a, b, columns 1–3). SREBP1c undergoes auto-regulation and increases *SREBF1* mRNA and SREBP1c protein levels^51^. Consistent with this, we found that PHF2 depletion increased the expression of *SREBF1* and SREBP1c downstream genes (Fig. 4c, columns 1–3). However, concomitant transfection with siSREBP1c abolished these effects in Hep3B cells (Fig. 4a–c, columns 4–6), indicating that SREBP1c is a vital transcription factor of the PHF2 pathway. In addition, SREBP1c knock-down increased endogenous PHF2 expression (Fig. 4a). Nile Red staining and flow cytometric analysis of Nile Red stained cells also showed that PHF2 loss-mediated lipid accumulation largely depended on SREBP1c (Fig. 4d, e, and Supplementary Fig. 6a). On the other hand, ectopic PHF2 expression decreased SREBP1c expression, total FFAs production, the mRNA levels of target genes of SREBP1c, and lipid accumulation in HCC cells (Supplementary Fig. 6c, e, g, i, k). The SREBP1c pathway plays a role in lipid metabolism and oncogenic signaling in various cancer types. To validate the influence of the PHF2/SREBP1c axis on cell proliferation, we performed a cell counting assay, colony formation assay, and a 3D culture based on oxygen-permeable polydimethylsiloxane (PDMS) to simulate in vivo conditions. PHF2 knockdown promoted the levels of proliferation-related genes and cell proliferating features, but SREBP1c co-knockdown abolished these effects in Hep3B cells (Fig. 4c, f, g, and Supplementary Figs. 6h, 7a, c, e–g). In contrast, the ectopic PHF2 expression abolished SREBP1c-induced cell proliferating features in Hep3B cells (Supplementary Figs. 6i, 7b, d, h, i). We also observed coincident results in the HepG2 cells (Supplementary Figs. 6 and 7). These results indicate that PHF2 engages SREBP1c regulation, thereby regulating lipogenesis and cell proliferation of HCC cells.

### A palmitic acid-enriched diet increases, but PHF2 decreases tumor progression in mice

**Fig. 5:**
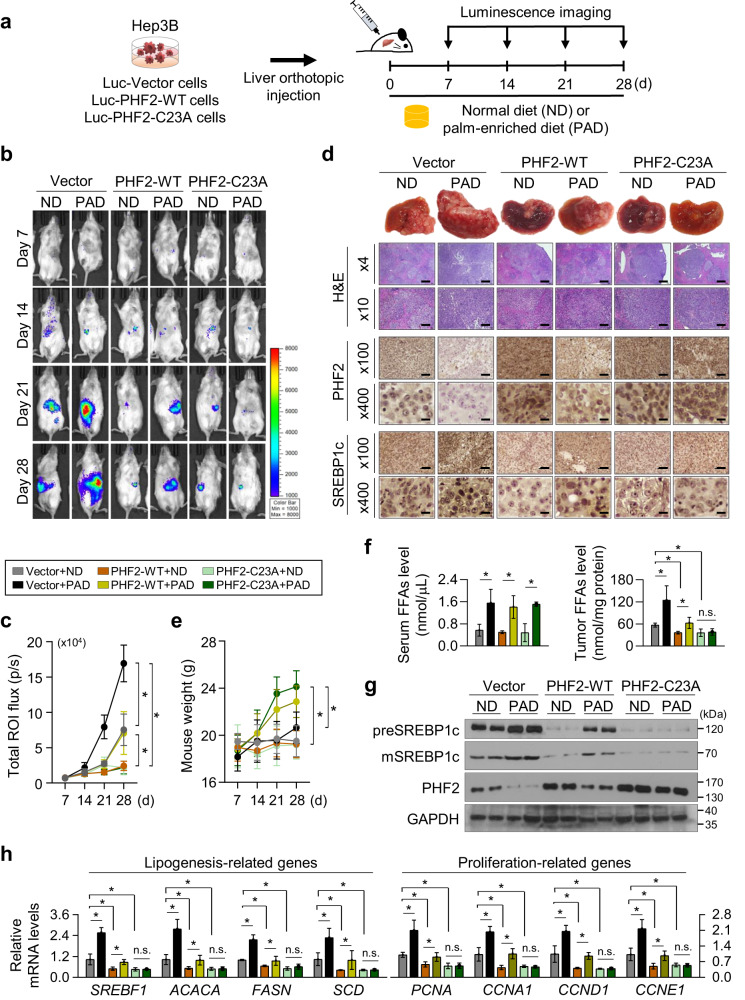
A palmitic acid-enriched diet increases, but PHF2 decreases mice tumor growth. **a** Schematic diagram of the in vivo model. Hep3B cells were transfected with luciferase-IRES-GFP-pcDNA (Vector), luciferase-IRES-GFP-WT-PHF2, or luciferase-IRES-GFP-PHF2-C23A. The cells from a single colony (5 × 10^5^) by G418 selection were transplanted by orthotopic injection into the livers of the immunodeficient NOD.Cg-*Prkdc*^*scid*^*Il2rg*^*tm1Wjl*^/SzJ (NSG) mice. Mice were fed a PA-enriched diet (PAD) or normal diet (ND) for 28 days (*n* = 7 independent animals in each group). **b** Bioluminescence images of mice obtained every 7 days after transplantation using the Xenogen IVIS® Lumina Spectrum. Color scale bars represent luminescence intensity ranging from low (purple) to high (red). **c** Total flux (photons/s/cm^2^/sr) was measured and growth curves were plotted based on the bioluminescence intensities. Mean ± SD (*n* = 7 independent animals in each group); *P < 0.05. **d** (Top) Representative images of liver morphology. (Bottom) Hematoxylin and Eosin (H&E) staining and immunohistochemical analysis of tumor sections using the indicated antibodies and DAB staining. The scale bar represents 400, 100, and 25 µm in images taken at ×4, ×100, and ×400 magnifications, respectively. **e** The body weight of mice was measured at the indicated time point; mean ± SD (*n* = 7 independent animals for each group); *P < 0.05. **f** Total FFAs levels in mice serum and tumor tissues were presented; mean ± SD (*n* = 3 independent samples); *P < 0.05. **g** The expression levels of the indicated proteins in tumors of mice were analyzed using western blotting. *n* = 3 independent experiments. **h** mRNA levels of lipogenesis- and proliferation-related genes in tumors were quantified relative to 18S mRNA; mean ± SD (*n* = 3 independent experiments); *P < 0.05. For the analyses in (**c, e, f, h**), statistical significance was evaluated by a two-tailed Student’s *t* test. The exact *p* values are presented in Supplementary Data 2. Source data are provided as a Source Data file.

We next explored the tumor-suppressive role of PHF2 and the dietary effect of PA in the liver microenvironment. Hep3B cells stably co-expressing luciferase with WT-PHF2 or PHF2-C23A were injected into the livers of NOD.Cg-*Prkdc*^*scid*^*Il2rg*^*tm1Wjl*^/SzJ (NSG) mice (Fig. 5a and Supplementary Fig. 8a). Each group was randomly divided into the normal diet (ND)- or PA-enriched diet (PAD)-fed groups. The Vector/PAD-fed group showed a significant increase in tumor growth compared to that observed in the Vector/ND-fed group on the bioluminescent and liver images (Fig. 5b–d, lanes 1–2, and Supplementary Fig. 8b). The PHF2-WT group showed reduced tumor growth compared to the Vector/ND-fed group, and tumor growth was somewhat increased by PAD feeding (Fig. 5b–d, lanes 3-4). Nevertheless, the PHF2-WT/PAD-fed group showed much-reduced tumor growth than the Vector/PAD-fed group. The PHF2-C23A group also showed dramatically reduced tumor growth than the Vector/ND group. The PHF2-C23A group did not exhibit noticeable effect on tumor growth when exposed to PAD feeding (Fig. 5b–d, lanes 5–6). This result indicates that the degradation of PHF2 protein, a result of palmitoylation, could be the cause of tumor growth by PAD feeding. Although the mice’s body weights in the ND groups showed no significant changes for one month, PAD feeding increased body weight in all groups except the Vector/PAD-fed group with a high tumor burden (Fig. 5e). FFA contents in the sera of all the PAD-fed groups were elevated. Still, FFAs in the tumor tissues were well matched with the tumor growth patterns (Fig. 5f). PAD feeding increased the SREBP1c protein level and expression of lipogenic genes; in contrast, their expressions were decreased in the WT- or C23A-PHF2 expressing groups. In addition, the PHF2-C23A group showed no further induction of SREBP1c protein and the levels of lipogenic genes by PAD feeding (Fig. 5d, g, h, and Supplementary Fig. 8c, d). As expected, the expression patterns of proliferation-related genes were consistent with those of lipogenesis-related genes (Fig. 5h). Overall, these results suggest that palmitoylation-dependent PHF2 degradation enhances both tumor growth and lipid metabolism in tumor-bearing mice.

We also tested whether PHF2 loss could increase the tumor growth of HCC cells. HepG2 cells stably expressing luciferase were transfected with siCtrl or siPHF2s and then injected into the livers of the NSG mice (Supplementary Fig. 9a). Each siRNA-treated group was randomly divided into two subgroups: fed an ND and a PAD. Bioluminescent images showed similar tumor growth in the siCtrl/PAD, siPHF2s/ND, and siPHF2s/PAD group, which were much larger than that observed in the siCtrl/ND group (Supplementary Fig. 9b, c). In addition, the mice in the PAD-fed groups showed slightly higher body weights than those in the ND-fed groups (Supplementary Fig. 9d). The liver tissues from the siCtrl/PAD-fed group and all siPHF2s-injected groups showed reduced PHF2 protein levels but increased SREBP1c protein level and expression of SREBP1c target genes compared to those from the siCtrl/ND-fed group (Supplementary Fig. 9e, f). The hepatic FFAs level showed similar results (Supplementary Fig. 9g). Moreover, we noticed a considerable rise in PHF2 palmitoylation levels in the remaining PHF2 despite a significant decrease in PHF2 protein levels by PAD feeding or siPHF2s (Supplementary Fig. 9h). These results indicate that PA increases tumor progression in a PHF2-dependent manner.

### PHF2 destroys SREBP1c protein as an E3 ubiquitin ligase

**Fig. 6:**
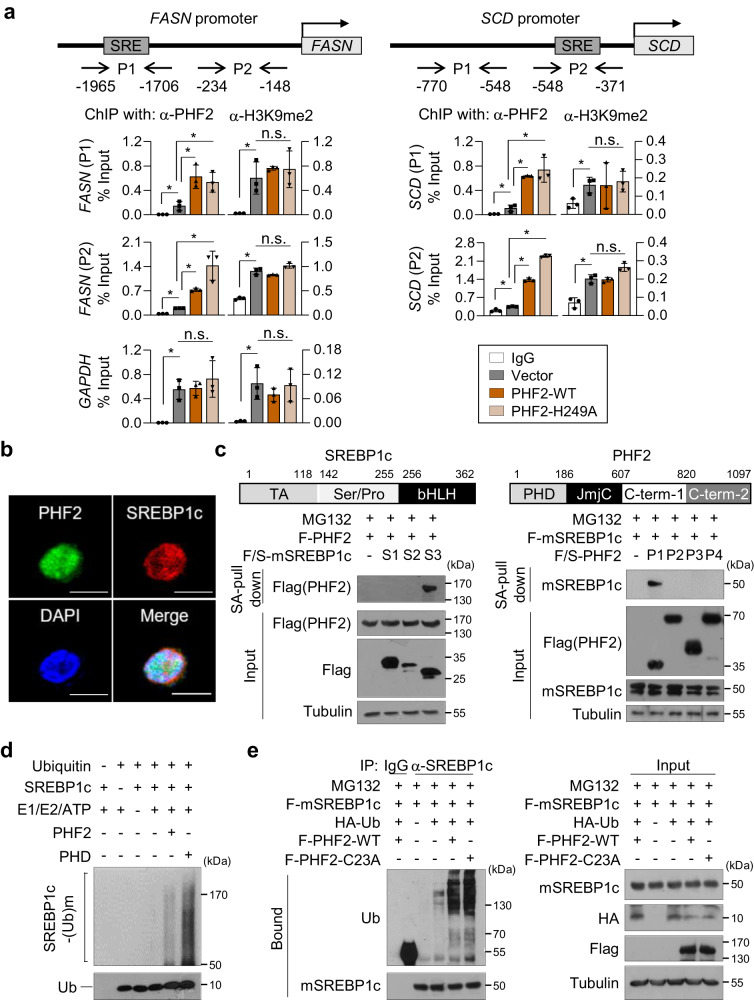
PHF2 destroys SREBP1c as an E3 ubiquitin ligase. **a** HepG2 cells were transfected with the indicated plasmids. Cell lysates were subjected to ChIP-qPCR assays using anti-H3K9me2 or anti-PHF2 antibodies at the *FASN* or *SCD* promoters. *GAPDH* was used as a negative control; mean ± SD (*n* = 3 independent experiments); *P < 0.05. Statistical analyses were based on a two-tailed unpaired t-test. The exact p-values are shown in Supplementary Data 2. P1, P2: promoter 1, 2; H3K9me2: histone H3 lysine 9 dimethylation. **b** Immunofluorescence analysis was performed using the indicated antibodies in Hep3B cells. *n* = 3 independent experiments. Scale bar = 10 µm. **c** (Top) Schematic diagram of the segments of SREBP1c or PHF2. The indicated plasmids were transfected into HepG2 cells (Bottom, left) or 293 T cells (Bottom, right). Then, cells were subjected to immunoprecipitation and immunoblotting after MG132 treatment for 8 h. Western blotting using the indicated antibodies evaluated the purified proteins using SA affinity beads. *n* = 3 independent experiments. TA: a transcription-activation domain; Ser/Pro: a serine- and proline-rich region; bHLH: a basic helix-loop-helix domain; PHD: a plant homeodomain; JmjC: a Jumonji C domain; C-term: C-terminus. **d** An in vitro ubiquitination assay was performed. The enzymatic reaction was stopped by adding a sample buffer and proteins were analyzed by western blotting. *n* = 3 independent experiments. **e** HepG2 cells were transfected with the indicated plasmids and then incubated with MG132 for 8 h. Cell lysates were pull-downed with the anti-SREBP1 antibody, and the pull-downed proteins were analyzed by western blotting using the indicated antibodies. *n* = 3 independent experiments. Source data are provided as a Source Data file.

Although PHF2 is known to relieve gene silencing and enhance gene transcription as an epigenetic activator^1^, our results indicated that PHF2 negatively regulated SREBP1c protein level and gene expression in HCC cells. These results prompted us to uncover the role of PHF2 beyond its classical function as a demethylase. Chromatin IP assay showed that WT-PHF2 and a PHF2 mutant (H249A) lacking demethylase activity were slightly but statistically significantly recruited into the SREBP1c-binding site at the promoter regions of *FASN* or *SCD*^52^. Despite this, it was observed in Fig. 6a that the H3K9me2 level remained unchanged. This result suggests that PHF2 has additional functions besides serving as a histone demethylase in its impact on SREBP1c. To understand the mechanism by which PHF2 decreases the expression of SREBP1c, the stability of SREBP1c was evaluated by measuring its half-life. Ectopically expressed PHF2 shortened the half-life of SREBP1c by 4.3-fold (Supplementary Fig. 10a), and MG132 treatment ameliorated PHF2-mediated SREBP1c reduction (Supplementary Fig. 10b). We next tested the interaction between SREBP1c and PHF2. Immunofluorescence analysis showed that SREBP1c and PHF2 were co-localized in the nucleus (Fig. 6b). PHF2 interacted with both endogenous or ectopically expressed SREBP1c (Supplementary Fig. 10c, d). SREBP1c was specifically bound to the PHD motif in PHF2 via its helix-loop-helix domain (Fig. 6c). Therefore, we assumed that PHF2 acts as a ubiquitin E3 ligase for SREBP1c because PHF2 has a PHD domain that acts as a ubiquitin E3 ligase^8,9^. To address this, we screened the major E2 enzymes for ubiquitination in a human liver dataset (Supplementary Fig. 10e). We performed an in vitro ubiquitination assay using the top six E2 enzymes from this dataset. The results showed that PHF2 ubiquitinated SREBP1c, specifically after the UbcH6-mediated E2 reaction (Supplementary Fig. 10f). Further, in vitro ubiquitination and IP assay confirmed that both the PHD motif and full-length PHF2 exerted E3 ubiquitin ligase activity toward SREBP1c (Fig. 6d and Supplementary Fig. 10g). Because the PHD motif was palmitoylated, we investigated whether palmitoylation of PHF2 could affect its E3 ligase activity. However, the E3 ligase activity of PHF2 did not differ in PHF2-WT and PHF2-C23A (Fig. 6e). Taken together, in HCC cells, PHF2 functions as an E3 ubiquitin ligase towards SREBP1c, irrespective of its palmitoylation status.

### The PA/PHF2/SREBP1c loop rewires lipogenesis and cell proliferation in HCC cells

**Fig. 7:**
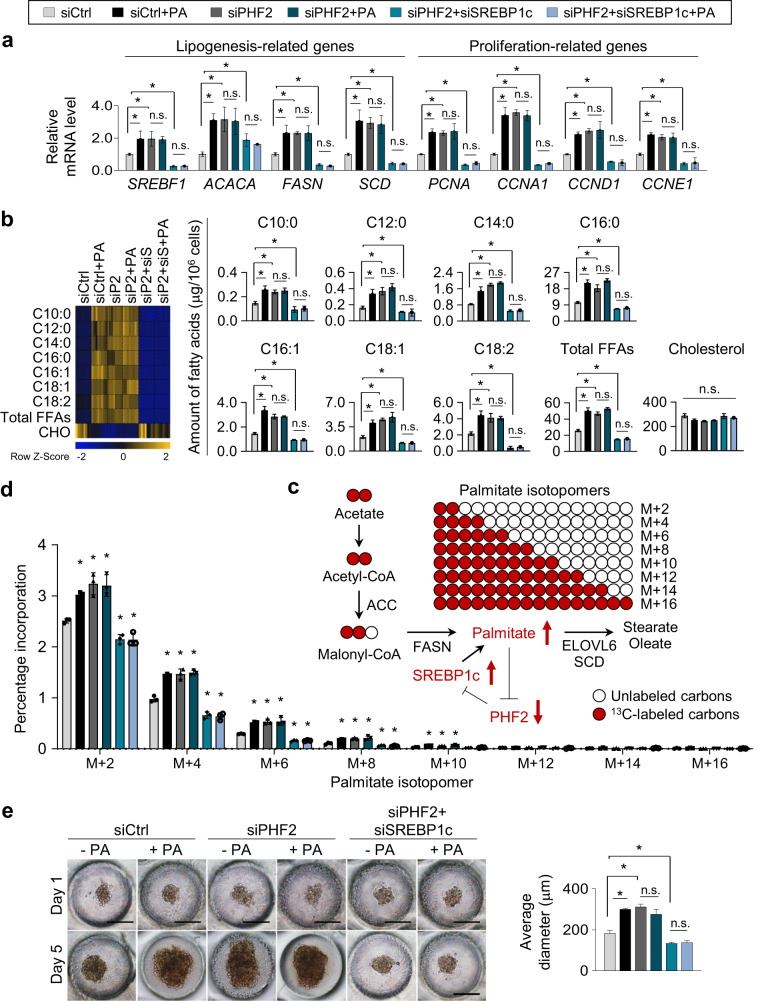
The PA/PHF2/SREBP1c loop rewires lipogenesis and proliferation in HCC cells. **a** Hep3B cells were transfected with the indicated siRNAs and incubated with PA for 24 h. The expressions of lipogenesis- and proliferation-related genes in Hep3B cells were quantified by RT-qPCR relative to 18S RNA; mean ± SD (*n* = 3 independent experiments); *P < 0.05. **b** Each FFA of HepG2 cells was calculated using GC-TOF/MS. The color scale bar represents relative expression values; blue shows a low expression score; yellow shows a high expression score. Mean ± SD (*n* = 8 independent samples); *P < 0.05. siS siSREBP1c. **c** Schematic diagram showing incorporation of 2-carbon units from ^13^C-labeled acetate into PA. **d** The de novo distribution of ^13^C-labeled even isotopomers of palmitate in HepG2 cells was measured using LC-MS. Mean ± SD (*n* = 3 independent samples); *P < 0.05. **e** Representative photographs of spheroid-formed cells on oxygen-permeable chips on 1 and 5 day. Scale bar = 200 µm. The right panel shows the average diameter of the spheroids quantified using ImageJ. Mean ± SD (*n* = 3 independent samples); *P < 0.05. For the analyses in (**a, b, d, e**), an unpaired two-tailed Student′s *t* test was conducted. The exact *p* values are presented in Supplementary Data 2.

We then wondered whether the PA/PHF2 axis could influence lipogenesis and cell proliferation in an SREBP1c-dependent manner. First, PA treatment or PHF2 knockdown increased lipogenesis- and cell proliferation-related gene expressions in HCC cells (Fig. 7a and Supplementary Fig. 11a, columns 1–4), whereas SREBP1c knockdown abolished it (Fig. 7a and Supplementary Fig. 11a, columns 5–6). Second, FFAs level obtained from GC-TOF/MS analysis also showed that PA treatment and siPHF2 increased a variety of FFAs, including PA (C16:0), in HepG2 cells in an SREBP1c-dependent manner (Fig. 7b). After synthesis by ACC and FASN, PA concentration is regulated by its elongation and desaturation into stearic (C18:0) and oleic acid (C18:1) by elongation of very long chain FAs protein 6 (ELOVL6) and SCD (Fig. 7c)^24^. To trace newly synthesized and elongated FAs via the PA/PHF2/SREBP1c axis, we used stable ^13^C-labeled acetate and monitored metabolites in HepG2 cells. PA treatment and PHF2 knockdown showed higher degrees of newly synthesized ^13^C-labeled FAs, such as myristic acid (C14:0) and PA. However, co-transfection of siPHF2 with siSREBP1c abolished this effect (Fig. 7d and Supplementary Fig. 11b). In addition, elongated and desaturated FAs from PA were increased in PA-treated and PHF2 knockdown HepG2 cells. However, the co-knockdown of SREBP1c abolished this effect (Supplementary Fig. 11c, d). These results coincided with spheroid formation obtained from 3D culture (Fig. 7e and Supplementary Fig. 11e, f). Thus, the PA↑ → PHF2↓ → SREBP1c↑ → PA↑ axis may be a vicious cycle that remodels lipid metabolism and cell proliferation in HCC cells.

### PHF2, ZDHHC23, and SREBP1c expression are reciprocally associated with clinical outcomes in HCC

**Fig. 8:**
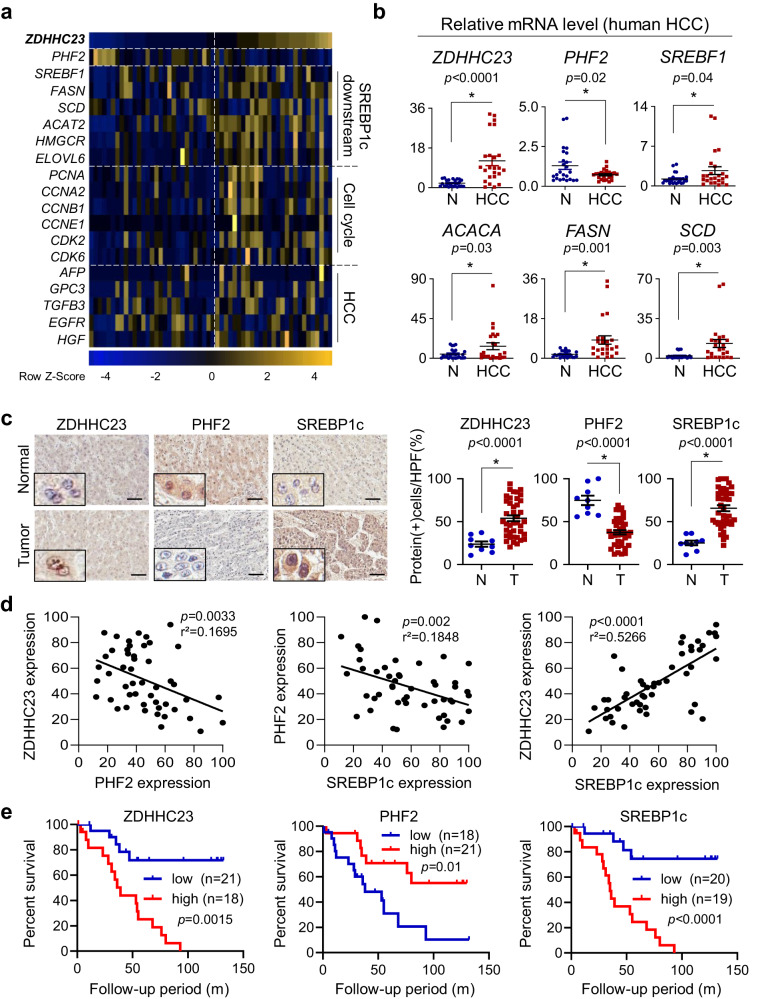
PHF2, ZDHHC23, and SREBP1c expression are reciprocally associated with clinical outcomes in HCC. **a** The profile of gene expressions of liver tissues obtained from the NCBI GEO dataset (GSE54238). A heatmap of gene expressions was displayed based on *ZDHHC23* expression values. The color scale bar represents relative expression values ranging from low (blue) to high (yellow). **b** The mRNA levels were measured in HCC tissues and adjacent normal liver tissues from 30 HCC patients. Comparisons were performed by the unpaired t-test; mean ± SD (*n* = 30 independent liver tissues). Statistical analyses were based on a two-tailed unpaired t-test. **c**–**e** Analysis of a human HCC tissue array containing 38 pairs of clinical HCC with corresponding nine normal tissues (SuperBioChips Lab, Seoul, South Korea). **c** (Left) Representative images of HCC tissues immunostained with the indicated antibodies. Scale bar = 60 µm. (Right) The staining scores were calculated based on immune-positive cell numbers; mean ± SD; *P* values were calculated by a two-tailed unpaired t-test. HPF a high-power field; N adjacent normal tissues; T HCC tissues. **d** A simple linear regression analysis examined correlations between proteins in HCC tissues. *r*^2^, a measure of goodness-of-fit, is calculated by comparing the sum-of-squares from the regression line with the sum-of-squares. **e** Kaplan–Meier analysis of the survival of HCC patients. Blue and red lines represent the indicated proteins’ low- and high-expression groups, respectively. *P* values were calculated using the log-rank test.

**Fig. 9:**
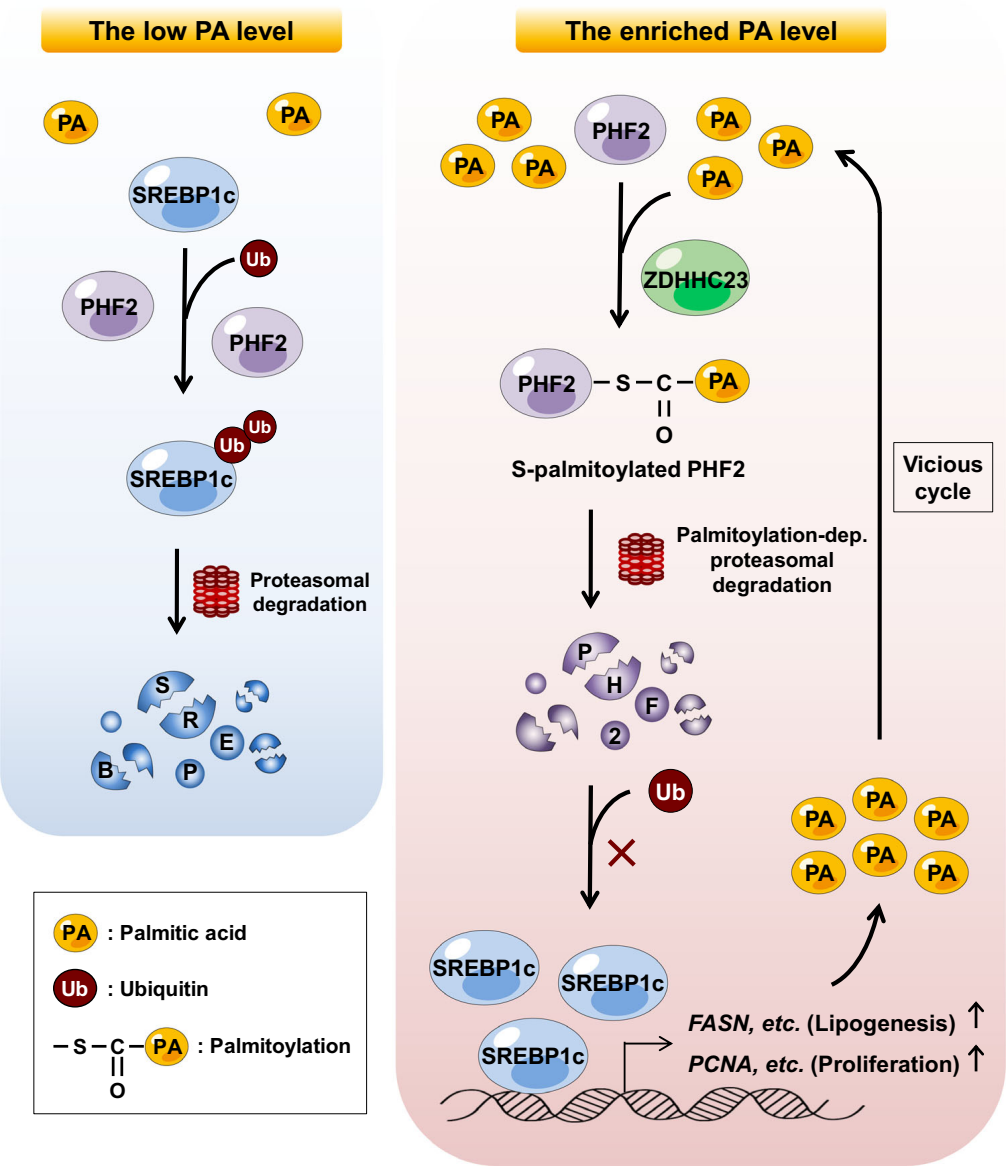
Diagram of the regulatory loop formed by the PA/PHF2/SREBP1c axis. Low PA cellular levels cause PHF2-mediated SREBP1c degradation (left panel). PA-enriched conditions enhance ZDHHC23-dependent PHF2 palmitoylation, resulting in the proteasomal degradation of PHF2. Following PHF2 degradation, SREBP1c becomes stable and induces PA levels in HCC cells (right panel). Finally, the vicious cycle of PA/PHF2/SREBP1c results in a poor HCC prognosis.

Finally, we analyzed the PA/PHF2/SREPB1c pathway using clinical samples. From the UALCAN web resources based on TCGA datasets, *ZDHHC23* expression was positively correlated with the HCC stage and tumor grade (Supplementary Fig. 12a). NCBI GEO dataset from HCC patients (GSE54238) indicated that *ZDHHC23* expression was negatively correlated with *PHF2* expression but positively correlated with the expression of *SREBF1*, its target genes, cell cycle-related genes, and HCC marker genes (Fig. 8a and Supplementary Fig. 12b). In the biopsied liver tissues from 30 HCC patients, *PHF2* mRNA was hardly expressed; in contrast, gene expressions of *ZDHHC23*, *SREBF1*, and FA biosynthetic enzymes were higher in HCC tissues than in adjacent normal tissues (Fig. 8b). Immunohistochemical analysis of liver tissues from HCC patients revealed higher ZDHHC23 and SREBP1c expression and lower PHF2 expression in cancer tissues than in adjacent normal tissues (Fig. 8c). Immunohistochemical analysis also confirmed the converse correlation between ZDHHC23/PHF2/SREBP1c (Fig. 8d). According to Kaplan–Meier survival analysis, patients with high PHF2 expression showed prolonged overall survival. In contrast, those with high ZDHHC23 or SREBP1c expression had shorter overall survival (Fig. 8e). Taken together, the feedback loop of PA/PHF2/SREBP1c critically modulates HCC progression. Therefore, it might be a potential prognostic marker in patients with HCC (Fig. 9).

## Discussion

PHF2 alterations are associated with pathophysiology in various organs. PHF2 is known as a histone demethylase and, by doing so, acts as a tumor suppressor. PHF2 participates in energy homeostasis and facilitates adipogenesis by epigenetically regulating transcriptional activities of C/EBPα/δ^53,54^. PHF2 removes the methyl group from methylated RUNX2 by SUV39H1, thereby promoting osteoblastogenesis^55^. Furthermore, PHF2 is essential for long-term memory consolidation after training as an epigenetic coactivator of CREB in the hippocampus^56^. In liver pathogenesis, PHF2 has dual effects. PHF2 induces simple hepato-steatosis by epigenetically coactivating ChREBP^57,58^. However, it protects the liver from fibrogenesis by facilitating Nrf2 activity, a major transcription factor in the defense against oxidative stress during non-alcoholic fatty liver disease^58^. Here, we found that PHF2 is an intrinsic tumor suppressor as a ubiquitin E3 ligase of SREBP1c in HCC cells.

Rapid cell proliferation requires an abundant supply of lipids for membrane synthesis and activation of signaling pathways; therefore, altered lipid metabolism is now considered a hallmark of cancer aggressiveness^59^. SREBP1c contributes to FA synthesis consequently promoting cancer cell survival^35,39^. Silencing of SREBP downstream genes reduces de novo FA synthesis and cancer proliferation, indicating that SREBPs-induced lipogenesis is required for cancer growth^60^. Furthermore, SREBP1a regulates all SREBP-responsive genes, whereas SREBP2 contributes to cholesterol homeostasis^61^. Our results indicate that the PA/PHF2 axis affects the FA profiles but not the cholesterol levels in HepG2 cells (Figs. 3e and 7b). This result suggests that SREBP1c could be a significant factor in this process.

SREBP1c is regulated at several levels in cells: transcription, proteolytic maturation, and posttranslational modification. After two sequential cleavages, mature SREBP1c enters the nucleus and binds to the target gene promoters^61^. Once SREBP1c is induced, an auto-amplification mechanism leads to its robust induction^51^. Regarding posttranslational modification, neddylation stabilizes SREBP1c at the protein level by competing with its ubiquitination^62^. SREBP1 is phosphorylated by GSK-3β, leading to FBW7-dependent SREBP1 ubiquitination^63^. RNF20 induces SREBP1c degradation upon PKA activation^64^. The SREBP1c protein level was more strongly induced in the siPHF2-transfected HepG2 cells than in the siFBW7- or siRNF20-transfected cells. On the other hand, siRNF20 induced SREBP1c expression in only A549 cells (Supplementary Fig. 13a, b), as Lee et al. reported^41^. The existence of other E3 ligases for SREBP1c ubiquitination in HCC cells is still open and remains possible.

Excess PA treatment and a diet rich in PA affect several signaling pathways. PA treatment in hepatocytes deregulates amino acid biosynthesis, including isoleucine and phenylalanine, with changes in NAD+-related metabolites^65^. Regarding immune pathways, PA increases proinflammatory cytokines such as IL-8 and IL-6 via an NF-κB pathway in hepatocytes^66^ and keratinocytes^67^, and PA-induced macrophage activation impairs insulin signaling in myocytes^68^. Furthermore, PA has been shown to affect cell fate; it induces a senescent phenotype in adipocytes by increasing the levels of γH2A.X DNA damage foci, telomere-associated foci, and p16INK4a^69^, and also increases endothelial senescence by negatively regulating autophagy through AMPK regulation^70^. PA overload causes cells to become apoptotic in pancreatic β-cells^71^. Regarding cancer progression, dietary PA promotes metastasis in oral carcinoma and melanoma in mice by increasing FA transporter CD36^12^. Moreover, PA treatment enhances the expression of stemness-related genes such as *SOX2* leading to cancer stem cell properties of HepG2 cells^23,72^. Our data demonstrate that PA exerts tumor-proliferating effects on HCC cells by rewiring FA metabolism through the PHF2/SREBP1c axis. Because PA has many tissue-specific functions, further studies to evaluate the molecular mechanisms of how PA affects cell signaling pathways are required.

Nearly 150 cancer studies have revealed genomic alterations in several ZDHHCs. Although there are no specific patterns of ZDHHC gene expression for different tumor types, palmitoylation of crucial signaling proteins has been identified in many cancers. For example, EZH2 palmitoylation by ZDHHC5 leads to the malignant progression of p53-mutant glioma^73^. In the liver, ZDHHC16-mediated PCSK9 palmitoylation promotes cell proliferation by activating PI3K/AkT pathway^74^. In this study, we found that expression of ZDHHC23 is significantly correlated with poor survival of HCC patients. In contrast, palmitoylation could suppress tumor growth. For example, ZDHHC6-mediated palmitoylation of AEG-1 enhances proteasomal degradation of AEG-1, suppressing HCC tumor growth^75^. ZDHHC7-mediated SCRIB palmitoylation enhances tumor suppressive activity of SCRIB^76^. Melanocortin 1 receptor (MC1R) palmitoylation by ZDHHC13 is essential for the activation of MC1R signaling and prevents melanomagenesis^77^. Differences in the expression of ZDHHCs and diverse palmitoylation substrates between tissues may explain these contrasting effects of palmitoylation on cancer.

Palmitoylation-mediated alteration of protein stability has been reported. Tlg1 palmitoylation stabilizes Tlg1 protein by inhibiting its interaction with the Tul1 RING E3 ubiquitin ligase^78^. Likewise, palmitoylation-deficient TEDA4 and TEM8 mutants undergo proteasomal degradation by interacting with E3 ubiquitin ligase U-box CHIP and Cbl, respectively^79,80^. Disruption of Fas palmitoylation enhances its lysosomal localization and subsequent lysosomal degradation^81^. PD-L1 palmitoylation stabilizes PD-L1 by internalizing it into recycling endosomes and keeps PD-L1 from lysosomal degradation^47^. We found that the ZDHHC23 palmitoylates PHF2 in a PA-abundant environment, and the palmitoylated PHF2 goes through ubiquitin-dependent degradation. Further studies of the interplay between palmitoylation and protein stability are required to unravel its diverse functions.

Through our research, we have uncovered a mechanism involving the PA/PHF2/SREBP1 axis that impacts lipid metabolism and cell proliferation in HCC cells. PA leads to the degradation of PHF2 through palmitoylation, which in turn induces SREBP1c and raises FFAs in HCC cells. This creates a vicious cycle where PA induction activates the PHF2/SREBP1c axis. Further research is required to ascertain the effectiveness of blocking the PA/PHF2/SREBP1c axis in an in vivo system, as many observations were made using in vitro systems. Finally, our research emphasizes the potential health hazards that a diet high in PA can pose and the negative effects on HCC progression.

## Methods

### Cell lines

HepG2 cells (No. 88065) were obtained from the Korea Cell Line Bank (Seoul, Korea). HEK293T (No. CRL-3216), Hep3B (No. HB-8064), PLC/PRF/5 (No. CRL-8024), and SK-HEP-1 (No. HTB-52) cells were obtained from the American Type Culture Collection (ATCC, Manassas, VA). The cell lines were genetically authenticated by the supplier based on growth and morphology. STR DNA profiling of the cell lines was conducted by the Korea Cell Line Bank. Cells were maintained in Dulbecco’s Modified Eagle’s Medium or Eagle’s Minimum Essential Medium supplemented with 10% fetal bovine serum (FBS) and 1% penicillin/streptomycin at 37 °C and 5% CO_2_.

### Orthotopic xenograft experiments

For the animal study, all experiments were performed by the guidelines of the Seoul National University Institutional Animal Care and Use Committee (approval No. SNU-200108-5-2). Mice were housed in a pathogen-free facility at a temperature of 22–26 °C and humidity of 40–60% under a 12-h light/12-h dark cycle. NOD.Cg-*Prkdc*^*scid*^*Il2rg*^*tm1Wjl*^/SzJ mice (NSG, female, 6 weeks old) were obtained from the Jackson Laboratory. As HepG2 and Hep3B cells are derived from human tumors, severe combined immunodeficient NSG mice were used to avoid tumor cell rejection. To establish stable cell lines, HepG2 cells were transfected with luciferase-IRES-GFP-pcDNA, and Hep3B cells were transfected with luciferase-IRES-GFP-pcDNA, luciferase-IRES-GFP-WT-PHF2 or luciferase-IRES-GFP-PHF2-C23A. Cells stably expressing indicated plasmids were selected with G418. To avoid transcriptional validation, a single colony was established and sub-cultured it. For gene silencing, PHF2-targeting siRNAs were transfected directly into HepG2 cells using in vivo-jetPEI transfection reagent (PolyPlus) on the first day of transplantation. To avoid gene recovery, siRNAs/jetPEI were administered via the tail vein after 5 d according to the manufacturer’s protocol. HepG2 cells (2 × 10^6^) and Hep3B cells (5 × 10^5^) were orthotopically injected into the livers of NSG mice. After injecting HCC cells, each group was randomly divided into two subgroups; one fed a normal diet or a custom-made 45% PA-enriched diet (RaonBio Inc., Gyeonggi-Do, Korea; Supplementary Table 3). Food consumption and body weight were measured, and tumor growth was monitored using the Xenogen IVIS Lumina in vivo imaging technology platform (Xenogen, Alameda, CA). At the end of the experiment, mice were sacrificed, and tumor tissues were stored at −80 °C for western blotting and RT-qPCR analysis. The free fatty acid levels in mice serum and tumor tissues were quantified using a free fatty acid assay kit (MAK044-1KT, Sigma, St. Louis, MO) according to the manufacturer’s protocol.

For ethics oversight, tumor growth could not be detected without bioluminescence monitoring because cancer cells were directly injected into the livers of mice. Thus, the tumor growth was monitored using the IVIS Lumina in vivo imaging technology platform (Xenogen) every week. Mice were monitored every other day for signs of pain such as hunched posture, highly reduced body weight, and reduced mobility after tumor cell injection. Euthanasia was considered in case of severe signs of pain even before the experiment’s endpoint. If euthanasia is needed, mice were subjected to 70% CO_2_, and complete euthanasia was confirmed by checking the tail pinch. No sex analysis was performed in the study design to avoid bias induced by sex.

### Human liver tissues for RT-qPCR analysis

For analyzing mRNA levels in human hepatocellular carcinoma (HCC), a total of 30 HCC tissues and the corresponding adjacent noncancerous specimens were obtained from the tissue bank of Seoul National University Hospital (Seoul, South Korea) with consent under approval from the Institutional Review Board of Seoul National University Hospital (approval No. C-1908-039-1053). Sex and/or gender were not considered in the study design to avoid bias induced by sex and/or gender. Detailed patient information is presented in Supplementary Table 4.

### Immunohistochemical analysis of human tissue array

An HCC tissue slide containing 38 pairs of clinical HCC with corresponding nine normal tissues was obtained from SuperBioChips Lab (Seoul, South Korea) for immunohistochemistry. Patient information is summarized in Supplementary Table 5. Tissue slides were dried for 1 h at 60 °C, dewaxed, autoclaved, and incubated with the primary antibody overnight at 4 °C, followed by the biotinylated secondary antibody for 1 h at room temperature. The immune complexes were visualized using a Vectastatic ABC kit (Vector Laboratories, Burlingame, CA) and counterstained with hematoxylin. The slides were photographed using a TMA scanner (Leica, APERIO AT1). The expression of proteins was evaluated based on intensity and positively stained cell number in four independent high-power fields on each sample.

### Treatment with fatty acids, 2-boromopalmitate, and MG132

Fatty acids were prepared as 100 mM stock solutions by dissolving them in EtOH. Next, fatty acids were complexed in 2% bovine serum albumin (BSA)-conjugated culture medium with 10% charcoal-stripped FBS and 1% penicillin/streptomycin. The mixtures were warmed to 56 °C. Before palmitic acid treatment, cells were pre-treated with 2-bromopalmitate (2-BP, 50 µM) or dimethyl sulfoxide (DMSO) for 24 h. Then, to block the proteasomal degradation of proteins, cells were treated with MG132 (10 µM) or DMSO for 8 h.

### Quantitative RT-PCR

Total RNAs were isolated using TRIzol reagent (Invitrogen, Carlsbad, CA), and cDNA synthesis was performed using an EasyScript cDNA Synthesis Kit (Applied Biological Materials, Richmond, BC, Canada). The cDNAs were amplified with EvaGreen qPCR master mix reagent using a StepOne Real-time PCR System. The nucleotide sequences of primers are summarized in Supplementary Table 1.

### Plasmids, small interfering RNAs, and transfection

Flag-tagged rat SREBP1c encoding the amino-terminal fragment of the wild-type SREBP1c protein with maximal activity (1–403 amino acids) was generated^82,83^. In addition, the SREBP1c-TA (1–118), SREBP1c-Ser/Pro (142–255), and SREBP1c-bHLH (256–362 amino acids) domains were amplified by PCR and inserted into Flag/steptavidin binding peptide (SBP)-tagged pcDNA. First, all small interfering RNAs (siRNAs) for each gene were synthesized by Integrated DNA Technologies (Coralville, IA). Then, according to the manufacturer’s instructions, plasmids and siRNAs were transfected into cells using Lipofectamine 2000 or Lipofectamine RNAiMAX (Thermo Fisher Scientific, Newark, DE). The sequences of siRNAs are listed in Supplementary Table 2.

### Immunoprecipitation, streptavidin pull-down assay, and immunoblotting

Cells were lysed with immunoprecipitation buffer (50 mM Tris-Cl, 100 mM NaCl, 1% NP-40, and 5 mM ethylenediaminetetraacetic acid (EDTA)) containing a protease inhibitor cocktail (Sigma). Cell lysates were incubated with EZview Red anti-FLAG M2 Affinity Gel (Sigma) for 4 h at 4 °C. For endogenous IP, cell lysates were incubated with the antibody for 16 h at 4 °C. The immune complexes were precipitated with protein A/G Sepharose beads (Sigma) for 4 h at 4 °C. To pull down the SREBP1c segments tagged with Flag/SBP vector, cell lysates were incubated with streptavidin affinity beads (GE17-5113-01, Sigma) for 16 h at 4 °C. The beads were washed five times with lysis buffer, and the proteins were eluted using denaturing sodium dodecyl-sulfate (SDS) sample buffer. Cell lysates were separated using SDS polyacrylamide gel electrophoresis (SDS-PAGE) and transferred onto Immobilon-P PVDF membranes (Millipore, Billerica, MA). The membranes were blocked with 3% milk or 3% BSA in tris-buffered saline containing 0.1% Tween 20 for 1 h and then incubated overnight with primary antibody at 4 °C. The membranes were incubated with horseradish peroxidase-conjugated secondary antibody for 1 h at room temperature and then visualized using an ECL Plus Kit (Thermo Fisher Scientific). The antibody sources for the immunoblotting analyses are listed in Supplementary Table 6. Source data are provided as Source Data and Supplementary Information files.

### Molecular Dynamics (MD) simulations

All-atom atomistic MD simulations were performed to investigate the PHD domain’s stability when palmitoyl-CoA is close to cysteine 23 (C23) and when C23 is palmitoylated. The initial 3D structure of PHF2 (amino acids 1–444) was constructed using the SWISS-MODEL homology modeling server^84^ with PDB file 8f8z^85^ to generate a missing structure for amino acids 66–85. The CHARMM36 forcefield was employed throughout the simulation for natural amino acid residues, the palmitoylated cysteine residue, and palmitoyl-CoA. In the simulation box, water molecules solvated each system with 100 mM NaCl as a periodic boundary condition. The proximity of palmitoyl-CoA to C23 in PHF2 was modeled by enforcing a distance restraint between the sulfur atom (S_C23_) of C23 and the acyl carbon (C_ac_; carbon double bonded to oxygen in the acyl group of palmitoyl-CoA). The optimal S_C23_-C_ac_ distance to be used as the restraint distance was determined using a combination of docking and subsequent steered MD simulations. Specifically, docking simulations of palmitoyl-CoA to the PHD domain were performed using the ZDOCK server^86^ to identify the potential binding sites and orientation of palmitoyl-CoA concerning the PHD domain. Steered MD simulations were then carried out to calculate the potential of mean force (PMF), representing the free energy change associated with palmitoyl-CoA experiencing its molecular environment as a function of the S_C23_-C_ac_ distance (*d*), using Jarzynski’s theorem^49^. After determining the optimal S_C23_-C_ac_ distance for the restraint, NPT-ensemble MD simulations with the distant restraint were carried out at 310 K and 1 bar using the NAMD program^87^. The Langevin thermostat^88^ and Langevin piston barostat^89^ were used for maintaining the temperature (310 K) and pressure (1 bar). The particle-mesh Ewald method employed a periodic electrostatic system with a 1 Å grid spacing^90^. The cutoff and switching distances for van der Waals forces were 12 Å and 10 Å, respectively. The bonds involving hydrogen were constrained to be rigid using the SHAKE algorithm. The MD system was equilibrated for 100 ns with a 2 fs time step, and the structure analysis was conducted by simulating a further 500 ns run and recording every 50 ps.

### Sample preparation for mass spectrometry

To identify PHF2-interacting proteins, HepG2 cells were transfected with the Flag/SBP-PHF2 plasmid. Proteins were immunoprecipitated using Flag or streptavidin affinity beads. For detection of palmitoylation sites of PHF2, HEK293T cells were transfected with Flag-PHF2 and immunoprecipitated with anti-Flag affinity beads. After washing with lysis buffer (LB buffer, 50 mM Tris-Cl, 150 mM NaCl, 10% NP-40, and 10% glycerol), the beads were divided into two groups, and only one group was treated with hydroxylamine (HAM, Sigma) to de-palmitoylate the palmitoylated cysteine residues. The beads were washed three times and eluted in 2X SDS buffer. The proteins were electrophoresed, subjected to in-gel digestion, and analyzed using liquid chromatography–mass spectrometry (LC-MS).

### LC-MS

PHF2 gel pieces cut from SDS-polyacrylamide gels were destained with 50% acetonitrile in 25 mM ammonium bicarbonate and then with 100% acetonitrile. To detect PHF2-interacting proteins, the proteins were reduced in the gels using 20 mM dithiothreitol at 56 °C for 1 h. To identify palmitoyl peptides in PHF2, 5 mM dithiothreitol was used at 37 °C^91^. After alkylation with 50 mM iodoacetamide in 25 mM ammonium bicarbonate for 1 h in the dark, the gel pieces were washed with 50 mM ammonium bicarbonate and then dehydrated in acetonitrile. Next, the dried gel pieces were rehydrated in a sequencing-grade modified trypsin (Promega) solution in 25 mM ammonium bicarbonate for digestion overnight. The tryptic peptides were extracted from the gel using 50% acetonitrile in 5% formic acid and 70% acetonitrile in 5% formic acid. Digested peptides of PHF2 were analyzed by Q-Exactive mass spectrometry with an Easy nLC-1000 pump and autosampler system (Thermo Fisher Scientific). The tryptic peptides were separated using a linear gradient of 5%–60% acetonitrile in water in 0.1% formic acid over 70 min. The mass spectrometer was operated in data-dependent mode with a full scan (m/z 350–2000) followed by MS/MS for each cycle’s top 20 precursor ions. The acquired MS/MS spectra were searched against the target (PHF2) database using SEQUEST software in Proteome Discoverer 1.4 (Ver. 1.4.0.288; Thermo Fisher Scientific). Two missed trypsin cleavages were allowed, and the peptide mass tolerances for MS/MS and MS were set to 0.5 Da and 10 ppm, respectively. Other parameters used for the SEQUEST searches included the variable modification of palmitoylation (+238.230 Da) and carbamidomethylation at cysteine (+57.021 Da), and oxidation at methionine (+15.995 Da). MS/MS spectra of palmitoylated peptides were manually validated to confirm peptide identification and palmitoylation site localization. The SEQUEST algorithm (Proteome Discoverer Ver. 1.4; Thermo Fisher Scientific) was used to detect the PHF2-interacting proteins (Supplementary Data 1).

### Lipid profiling using gas chromatography-mass spectrometry time-of-flight (GC-TOF/MS) analysis

Cells (1 × 10^6^) were washed and collected with cold phosphate-buffered saline (PBS). After centrifuging at 2800 × *g* for 5 min, samples were added with methyl tert-butyl ether (MTBE) and vortexed until cells were suspended entirely. For internal standard (IS), oleic acid-d_17_ (C18:1-d7) was diluted with MTBE at 10 µg/mL concentration. The commercial information is as follows: https://www.caymanchem.com/product/9000432/oleic-acid-d17. First, a 10 µL volume of IS was spiked, and then 200 µL of water was added to induce phase separation. After vortex and centrifugation, supernatants were collected and dried under nitrogen streams. Dried residues were reconstituted with 100 µL of N-methyl-N-(trimethylsilyl)trifluoroacetamide. The sample was transferred into a glass autosampler and derivatized for an hour at 70 °C.

GC-MS analysis was performed using an Agilent 7890B GC system (Agilent, Santa Clara, CA, USA) coupled with a LECO Pegasus HT TOF/MS device (LECO, St. Joseph, MI, USA). A 1 µL aliquot of each prepared sample was injected into the GC device with a front-inlet split ratio of 20. After the injection, the lipids were separated using an Rtx-5MS column (Restek, Bellefonte, PA, USA). The GC oven temperature was 50 °C initially, increased at 50 °C/min to 150 °C, and at 10 °C/min to 330 °C. The transfer line and ion source temperatures were 280 °C and 250 °C, respectively. Electron ionization was set to be 70 eV. The mass spectrometer operated at a 50–500 mass-to-charge ratio (m/z). The data were processed using ChromaTOF 4.6 (LECO) to determine the concentration of each compound. The unique mass of target compounds defined by ChromaTOF was used as the quantification ions. The concentrations were calculated using each lipid standard with the calibration range of 0.7–10 µg/mL, whereas 3–1000 µg/mL for palmitic acid and cholesterol.

### Stable-isotope tracing using LC-MS

Cells (4 × 10^6^) were quenched with 1 mL of methanol. After centrifuging at 2800 × *g* for 5 min, the supernatants were collected in a glass tube. A 10 µL volume of heptadecanoic acid (100 µg/mL; Sigma-Aldrich) was spiked and used as an IS. The samples were vortexed and dried using a nitrogen evaporator. Then, samples were reconstituted with 100 µL of 2-propanol (IPA): acetonitrile (ACN): water (2:1:1, v/v/v). Analysis of ^13^C-labeled FAs was performed using LC-MS (Synapt G2-S, Waters, Milford, MA, USA). A 5 µL sample was injected into an ACQUITY BEH C18 column 1.7 µm (2.1 × 100 mm). The column temperature was kept at 40 °C. LC mobile phases A and B comprise 10 mM ammonium formate (AmF) in 40% ACN and 10 mM AmF in IPA: ACN (9:1, v/v), respectively. The flow rate was kept at 0.25 mL/min, and the separation gradient elution was as follows: 40% B at 0 min, 40 to 65% B at 5 min, 50 to 100% B in 5 min, and 40% B in 3 min. The MS data were acquired using electrospray ionization (ESI) in the negative mode, with a scan range of 50-1000 m/z. The molecular mass of compounds was accurately determined using leucine-enkephalin as a lock mass calibrant^92^. Fatty acids were identified as matching the retention time and m/z values of the fatty acid analytical standard. Isotopologues of fatty acids were determined with m/z < 0.01 of their theoretical monoisotopic mass and retention time window within 0.2 min of their corresponding fatty acids. The LC-MS data were processed by QuanLynx (Ver. 4.2, Waters, Milford, MA, USA), and ElemCor was used to correct natural isotope abundance^93^.

### Palmitoyl-CoA determination using LC-MS analysis

Cells (3 × 10^6^) were quenched with 1 mL of methanol containing 5% formic acid. The supernatants were collected after centrifugation at 2800 × *g* for 5 min and dried using a nitrogen evaporator. The dried extracts were reconstituted with 100 µL of 50% methanol containing 5% formic acid. A 5 µL sample was injected into Synapt G2-S with an ACQUITY BEH C18 column 1.7 µm (2.1 × 100 mm). The column temperature was kept at 40 °C and was eluted in a gradient mode of mobile phase A: 10 mM ammonium bicarbonate in water and B:ACN. Linear gradient elution was as follows: 5% B at 0 min, 5 to 90% B in 2 min, 90 to 5% B in 1 min, and equilibrated for 10 min at 5% B. The flow rate was kept at 0.25 mL/min. Selected reaction monitoring (SRM) data were acquired using ESI in positive mode with 40 V as collision energy. SRM transition was 1006.4 > 499.5 m/z. The data were analyzed using QuanLynx (Ver. 4.2).

### Acyl-biotin exchange (ABE) palmitoylation assay

Cells were lysed in LB buffer with 50 mM N-ethylmaleimide (Sigma) containing a protease inhibitor cocktail. Endogenous PHF2 was purified using specific antibodies and protein A/G Sepharose beads, and Flag-PHF2 was immunoprecipitated with anti-Flag affinity beads. Purified PHF2 protein was divided into two groups. One group was treated with only LB buffer, and the other was treated with LB buffer + 1 M HAM for 1 h at room temperature. The beads were incubated with EZ-Link™ BMCC-Biotin (Thermo Fisher Scientific) for 1 h at 4 °C. Beads were eluted in 2X SDS sample buffer and used for western blotting analysis with streptavidin-HRP^77,94^.

### Nuclear extraction

HCC cells were washed with ice-cold PBS and collected by scraping and centrifugation at 700 × *g* for 5 min at 4 °C. First, the resulting cell pellets were lysed using extraction buffer (20 mM Tris-Cl, 1.5 mM MgCl_2_, 10 mM KCl, 0.2 mM EDTA, 0.5 mM dithiothreitol, and 0.5 mM phenylmethylsulfonyl fluoride) with the addition of a protease inhibitor cocktail, and incubated on ice for 10 min. And then, 0.6% NP-40 was added, and cell lysates were centrifuged at 2,800 × *g* for 5 min at 4 °C to obtain the cytosolic supernatant. Next, the pellets were resuspended using an extraction buffer containing 5% glycerol and 400 mM NaCl and incubated on ice for 30 min. Next, lysates were centrifuged at 11,000 × *g* for 10 min at 4 °C, and the nuclear fraction was obtained as the supernatant.

### Immunofluorescence analysis

Cells were fixed with 4% paraformaldehyde for 10 min, permeated with PBS containing 0.3% Triton X-100. After blocking with PBS containing 3% BSA and 0.3% Triton X-100 for 1 h at room temperature, cells were incubated with primary antibody at 4 °C overnight. After washing three times with PBS, cells were incubated with fluorescent conjugated secondary antibody for 1 h at room temperature. The nuclei of cells were stained with DAPI. The slides were mounted with FluorSave™ (Merck Millipore, Billerica, MA). Images were observed using a confocal microscope (FV3000; Olympus, Tokyo, Japan).

### Lipid accumulation assay using Nile Red

A stock solution of Nile Red was prepared at 1 mg/mL in dimethyl sulfoxide (DMSO) and protected from the light. Cells were washed twice with PBS and fixed with 4% formaldehyde solution for 10 min at room temperature. After fixation, cells were rinsed three times with PBS and incubated with Nile Red solution at a final concentration of 1 μg/mL in PBS for 20 min at 37 °C, followed by incubation with DAPI for 1 min in the dark. The slides were mounted with FluorSave™ Reagent for visualization under a fluorescence microscope. For flow cytometric analysis, cells were washed with PBS and stained with Nile Red solution at 10 ng/mL concentration in PBS for 20 min at 37 °C in the dark. The cells were stained with DAPI for 2 min at room temperature. Finally, the stained cells’ lipids were analyzed using a flow cytometer (FACSymphony A5, BD Biosciences).

### Cell proliferation assay

At 24 h post-transfection, the cells were digested with trypsin and resuspended as a single-cell suspension. Aliquots of 3 × 10^5^ cells were seeded and harvested daily, and cell numbers were counted. Each experiment was performed in triplicate.

### Colony formation assay

HepG2 cells (5 × 10^3^ cells/well) and Hep3B cells (1 × 10^4^ cells/well) were grown for 14 days at 37 °C and 5% CO_2_. To avoid gene recovery, siRNAs were transfected every 3 days. In addition, the cells were fixed with methanol and stained with freshly prepared 0.5% crystal violet for 40 min at room temperature. Each experiment was performed in triplicate.

### Oxygen-permeable 3D culture

PDMS prepolymer was prepared by mixing the base and curing agent (CAT-1300; Shin-Etsu Chemical, Tokyo, Japan) at a ratio of 10:1. After removal of air bubbles using a vacuum desiccator, and the prepolymer was poured into a negative mold for the second molding process and stored at 4 °C overnight. After solidification for 2 h at 60 °C, the PDMS substrate was peeled off from the device, immersed in water, and autoclaved. Before use, PDMS chips were laid into 6-well plates with a round hole (2.5 × 2.5 cm) in the central region to avoid inhibition of oxygen permeation. The Oxy chips were then incubated with 2 mL of 4% Pluronic F-127 solution for 12 h and rinsed thrice with warmed PBS^95,96^. Cells were inoculated into the chips at a cell density of 1 × 10^6^ cells, and the culture medium was changed daily. Spheroids formed on the Oxy chips were photographed daily, and the average spheroid diameter was quantified using ImageJ (Ver. 1.52a). Full-size images and the average spheroid diameter are presented in Supplementary Figs. 7 and11.

### In vitro palmitoylation

F/S-PHF2 and His-ZDHHC23 were transfected into 293T cells. Cells were lysed in a palmitoylation buffer containing 50 mM Tris-HCl (pH 8.0), 250 mM NaCl, 5% glycerol, 5 mM β-mercaptoethanol, 40 mM n-dodecyl-beta-maltoside detergent (DDM, Thermo Fisher Scientific), and a protease inhibitor cocktail. Flag- and His-tagged proteins were pulled down using Flag affinity and nickel-NTA beads (QIAGEN, Hilden, Germany). After washing with a palmitoylation buffer containing 2 mM DDM, proteins were eluted with 3X FLAG peptide (APExBIO, Houston, TX) or 250 mM imidazole containing 2 mM DDM. Purified PHF2 (1 µM) and ZDHHC23 (100 nM) were diluted in reaction buffer (50 mM HEPES (pH 7.0), 200 mM NaCl, 0.2 μM DTT, 1 mM EDTA, and 300 μM DDM). The reaction was initiated by adding 1 μM 16-NBD-16:0 Coenzyme A (Avanti Polar Lipids, Birmingham, AL) at room temperature. The samples were quenched with non-reducing SDS buffer and separated in SDS-PAGE gels. The Gel-Doc EQ system detected band fluorescence (BIO-RAD, Hercules, CA).

### In vitro ubiquitination

For enzyme purification, 293T cells were transfected with F/S-PHF2 or F/S-PHD domain and lysed with immunoprecipitation buffer. The cell lysates were incubated with Flag affinity gel and washed with a buffer. The pulled-downed proteins were eluted in 3X FLAG peptide. Ubiquitination assay for SREBP1c recombinant protein (Abnova, Taipei, Taiwan) was carried out using a ubiquitinylation kit according to the manufacturer’s protocol (Enzo Life Sciences, Farmingdale, NY). Reactions were terminated with an SDS sample buffer after 4 h incubation at 37 °C.

### Statistical methods

All data were analyzed using Microsoft Excel 2010 (Microsoft) or GraphPad Prism Software (Ver. 8.0.2), and results are expressed as the mean ± standard deviation (SD). The two-tailed unpaired Student’s *t* test was used to compare the means of the two groups. A simple linear regression analysis by GraphPad Prism Software (Ver. 8.0.2) computed correlations between two groups. *r*^2^, a measure of goodness-of-fit, was calculated by comparing the sum-of-squares from the regression line with the sum-of-squares. The survival rate of patients from the tissue array was assessed using the Kaplan–Meier method. In all analyses, *P < 0.05 was taken to indicate statistical significance. The exact p-values are provided in Supplementary Data 2.

### Reporting summary

Further information on research design is available in the Nature Portfolio Reporting Summary linked to this article.

### Supplementary information

Supplementary Information

Description of Additional Supplementary Information

Supplementary Data 1

Supplementary Data 2

Reporting Summary

### Source data

Source Data

## Data Availability

Structural difference between wild-type and mutant residues of protein (PDB file 3kqi) was predicted using Dynamut (http://biosig.unimelb.edu.au/dynamut/). Total PHF2-interacting proteins were annotated using DAVID Bioinformatics Resources (https://david.ncifcrf.gov/chartReport.jsp?d-16544-p=1&d-16544-o=1&annot=85&d-16544-s=5). The mass spectrometry proteomics data have been deposited to the ProteomeXchange Consortium via the PRIDE repository with the dataset accession number PXD044277. The public NCBI dataset was acquired under accession codes GSE54238 and GSE89632. Arraystar human lncRNA microarray GPL16955 and GPL14951 platforms were obtained from GEO public database. Publicly available databases from UALCAN web resources (https://ualcan.path.uab.edu/) were used for analyzing ZDHHC23 expression. Source data are provided with this paper.
