# Supplementary material for: Palmitoylation-driven PHF2 ubiquitination remodels lipid metabolism through the SREBP1c axis in hepatocellular carcinoma
Source: Nat Commun. 2023 Oct 12;14:6370. doi: 10.1038/s41467-023-42170-0 (PMC10570296; doi:10.1038/s41467-023-42170-0)
Supplement: Supplementary file 1 — Supplementary Information [file 41467_2023_42170_MOESM1_ESM.pdf]

Supplementary Information for

Palmitoylation-driven PHF2 ubiquitination remodels lipid metabolism through the SREBP1c axis in hepatocellular carcinoma

Do-Won Jeong[1,2], Jong-Wan Park[1,3], Kyeong Seog Kim[1,4], Jiyoung Kim[1], June Huh[5], Jieun Seo[1,2,6], Ye Lee Kim[1,2], Joo-Youn Cho[1,4], Kwang-Woong Lee[7], Junji Fukuda[6], and Yang-Sook Chun[1,2,3]

[1]Department of Biomedical Sciences, Seoul National University College of Medicine, Seoul, 03080, Korea

[2]Department of Physiology, Seoul National University College of Medicine, Seoul, 03080, Korea

[3]Ischemic/Hypoxic Disease Institute, Seoul National University College of Medicine, Seoul, 03080, Korea

[4]Department of Clinical Pharmacology and Therapeutics, Seoul National University College of Medicine and Hospital, Seoul, 03080, Korea

[5]Department of Chemical and Biological Engineering, Korea University, Seoul, 02841, Korea

[6]Faculty of Engineering, Yokohama National University, Yokohama, 240-8501, Japan

[7]Department of Surgery, Seoul National University College of Medicine, Seoul, 03080, Korea

Supplementary Figures **2**

Supplementary Tables **15**

Supplementary Figures

Supplementary Fig. 1

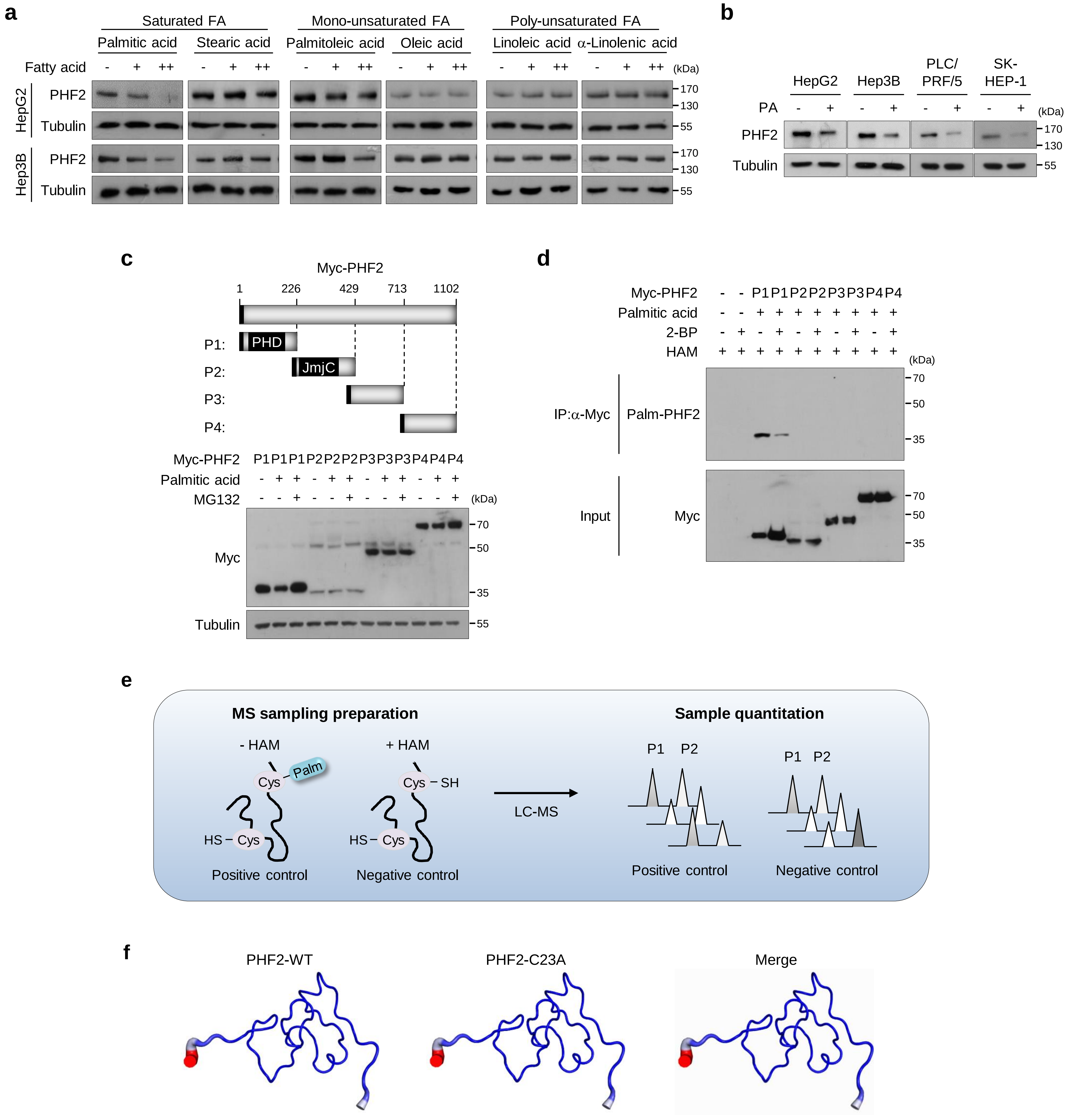

Supplementary Fig. 1: PHF2 is palmitoylated at the C23 residue and a point mutation from cysteine to alanine causes no structural change in the PHF2 protein. **a** HepG2 and Hep3B cells were treated with bovine serum albumin (BSA)-conjugated fatty acid (FA, 0, 150, 300 μM) for 24 h. Proteins were eluted and subjected to SDS-PAGE. **b** Hepatocellular carcinoma (HCC) cells were treated with palmitic acid (PA, 300 μM). Proteins were eluted and subjected to western blotting. **c** (Top) Schematic diagram of Myc-PHF2 segments. (Bottom) 293T cells were transfected with Myc-PHF2 fragments and treated with PA for 24 h and MG132 (10 μM) for 8 h. **d** After transfection with Myc-PHF2 domains, 293T cells were pre-treated with 2-bromopalmitate (2-BP, 50 μM) or dimethyl sulfoxide (DMSO), and incubated with PA, followed by MG132. PHF2 palmitoylation was detected after an immunoprecipitation (IP) assay with an anti-MYC antibody, and proteins were subjected to an acyl-biotin exchange (ABE) assay. Palm-PHF2: palmitoylated PHF2. **e** Schematic illustration of sample preparation for liquid chromatography-mass spectrometry (LC-MS) (related to Fig. 1c). **f** Prediction of atomic fluctuation from Dynamut. The structure provides the amplitude of the absolute atomic motion. The magnitude of the fluctuation is represented by tubes colored blue (low), white (moderate), and red (high). Supplementary Fig. **1a-d** were performed in triplicate.

Supplementary Fig. 2

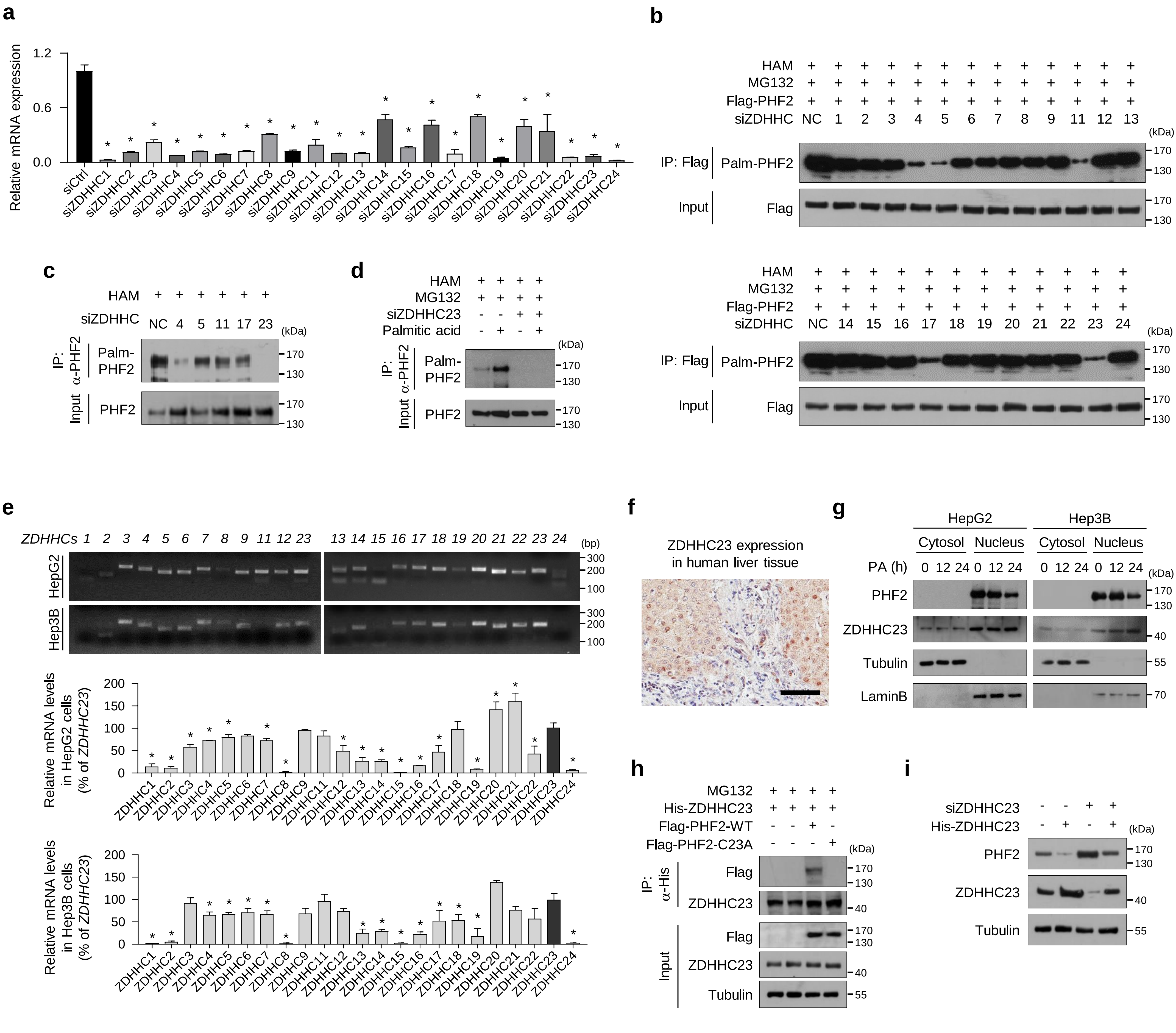

Supplementary Fig. 2: ZDHHC23 is a major palmitoyltransferase of PHF2.

a Verification of 23 siRNAs targeting 23 ZDHHCs. 293T cells were transfected with the indicated siRNAs and subjected to RT-qPCR. Mean ± SD (n = 3 independent samples); *P < 0.05. Statistical analyses were based on a two-tailed unpaired t-test. **b** After transfection with the indicated siRNAs and Flag-PHF2, 293T cells were treated with MG132 for 8 hours. After immunoprecipitation using Flag affinity beads, the precipitated proteins were subjected to ABE assay. **c** ABE assay to detect endogenous PHF2 palmitoylation. HepG2 cells were transfected with the indicated siRNAs targeting ZDHHCs, followed by IP using an anti-PHF2 antibody. Palmitoylated PHF2 was detected using SDS-PAGE. NC: negative control. **d** HepG2 cells were transfected with siZDHHC23 and incubated with PA, followed by treatment with MG132. After immunoprecipitation using an anti-PHF2 antibody, palmitoylated PHF2 was detected using ABE assay. **e** (Top) Representative agarose gel images from qPCR assay. (Bottom) Relative mRNA expressions of each *ZDHHC* expressed in HepG2 and Hep3B cells are presented as a percentage of *ZDHHC23*. Mean ± SD (n = 3 independent samples); *P < 0.05 by an unpaired two-tailed Student′s t-test. **f** Representative images of human liver tissues immunostained with an anti-ZDHHC23 antibody. Scale bar = 20 µm. **g** HepG2 and Hep3B cells were subjected to nuclear extraction assay after PA treatment. The cytoplasmic to nuclear ratio = 8:2. Indicated proteins were analyzed by western blotting. **h** HepG2 cells were transfected with the indicated plasmids and treated with MG132 for 8 h. After immunoprecipitation using an anti-His antibody, precipitated proteins were examined using immunoblotting. **i** Western blotting of lysates from HepG2 cells transfected with siRNAs or plasmids. Supplementary Fig. **2b-d** and **2f-i** were performed in triplicate. The exact p-values are provided in Supplementary Data 2.

Supplementary Fig. 3

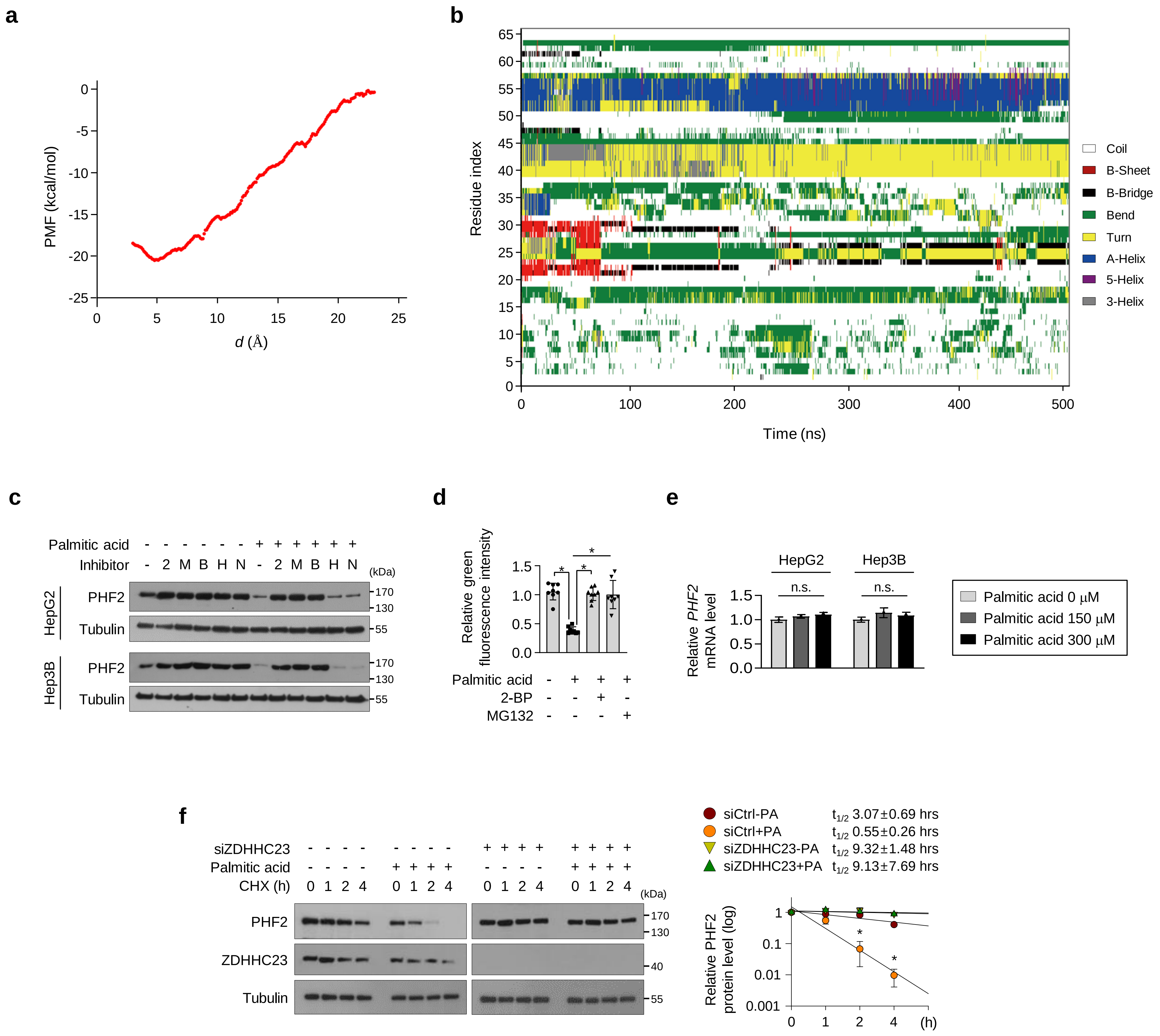

Supplementary Fig. 3: ZDHHC23-mediated PHF2 palmitoylation enhances its ubiquitination.
a The optimal S_{C23}-C_{ac} distance was determined using a combination of docking simulations and subsequent steered molecular dynamics simulations. Potential of mean force (PMF) profiles as a function of the distance (*d*) between the sulfur atom (S_{C23}) of C23 and the acyl carbon (C_{ac}; a carbon double bonded to oxygen in the acyl group of palmitoyl-coenzyme A). **b** The dictionary of protein secondary structure analysis for PHD motif in the presence of palmitoyl-coenzyme A (d = 5 Å). **c** Cells were pre-treated with 2-BP or DMSO and incubated with PA, followed by treatment with MG132 (M), bortezomib (B), hydroxychloroquine (H), and NH_4Cl (N). PHF2 levels were measured using immunoblotting. **d** The intensity of green fluorescence (related to Fig. 2f) was quantified using ImageJ. Mean ± SD (n = 3 independent samples); $^*P < 0.05$. **e** After treatment with PA (0, 150, and 300 μM), *PHF2* mRNA levels in HCC cells were determined using RT-qPCR. Gene expression was quantified relative to 18S RNA. Mean ± SD (n = 3 independent experiments). n.s.: non-significant. **f** HepG2 cells were transfected with siZDHHC23 and treated with PA for 24 h, followed by incubation with cycloheximide (CHX, 50 μg/mL). Band intensities were quantified using ImageJ and the half-life of PHF2 was measured. Mean ± SD (n = 3 independent experiments); $^*P < 0.05$. For the analyses in (**d-f**), an unpaired two-tailed Student′s t-test was conducted. Supplementary Fig. **3c,f** were performed in triplicate. The exact p-values are provided in Supplementary Data 2.

Supplementary Fig. 4

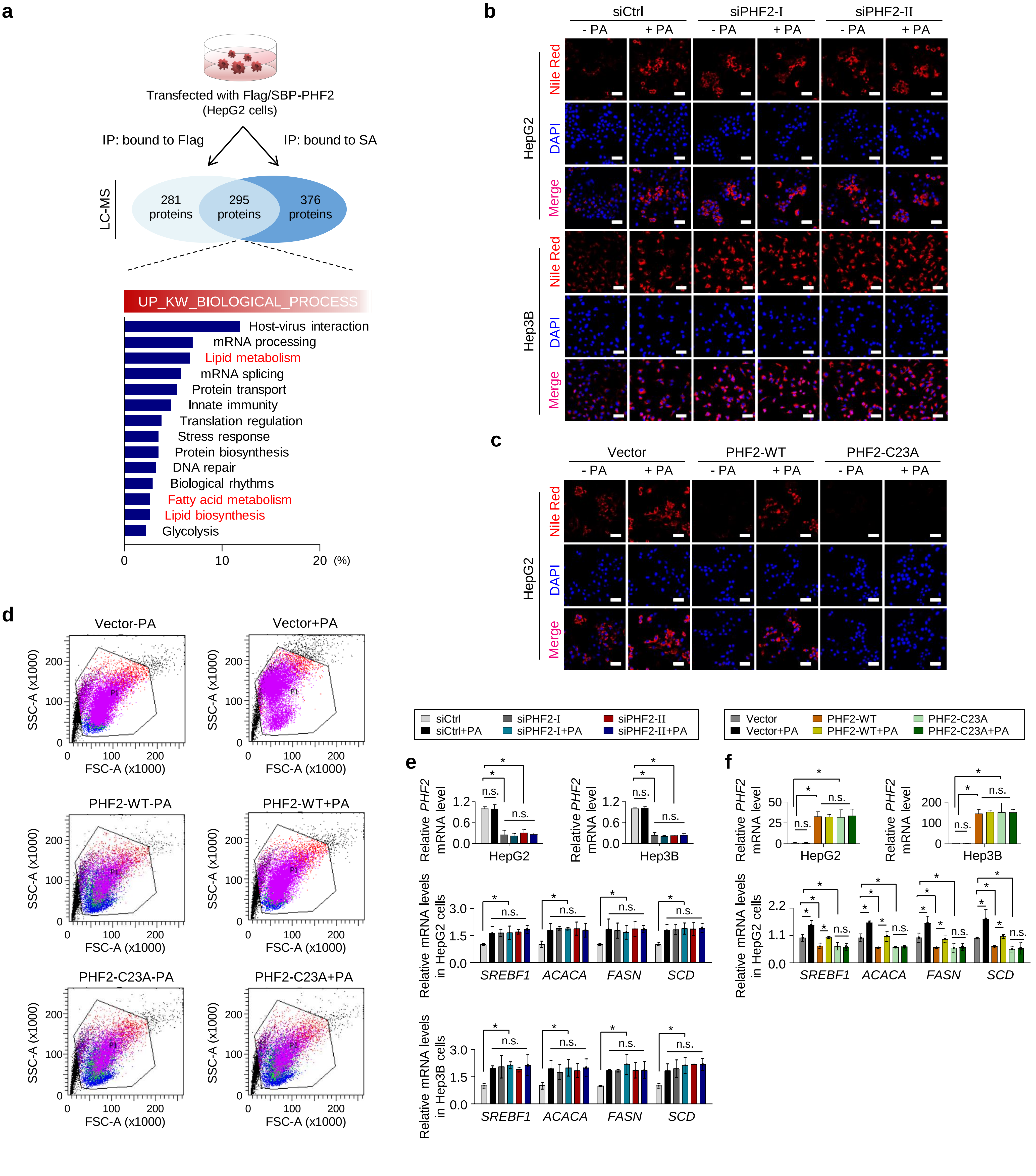

Supplementary Fig. 4: PHF2 is negatively associated with lipogenesis in HCC cells.

a (Top) Schematic diagram of LC-MS combined with IP (related to Fig. 3a). HepG2 cells were transfected with a Flag/SBP-PHF2 plasmid. Proteins were precipitated using Flag or SA affinity beads and co-expressed proteins were analyzed by LC-MS. (Bottom) Bar chart illustrating the biological process of PHF2-interacting proteins according to a DAVID annotation tool. **b-e** HCC cells were transfected with the indicated siRNAs or plasmids, and treated with PA. (**b**,**c**) Cells were fixed with formaldehyde, stained with Nile Red and DAPI, and visualized by fluorescence microscopy. Scale bar = 60 µm. (**d**) For quantification, a flow cytometric analysis of Nile Red stained cells was performed (related to Fig. 3c). Fluorescence was read at excitation = 550 nm. SSC: the side scatter parameter; FSC: the forward scatter parameter. (**e**,**f**) Gene expression levels were quantified using RT-qPCR relative to 18S RNA. Mean ± SD (n = 3 independent experiments); *P < 0.05 by a two-tailed unpaired t-test. Supplementary Fig. **4b-c** were performed in triplicate. The exact p-values are presented in Supplementary Data 2.

Supplementary Fig. 5

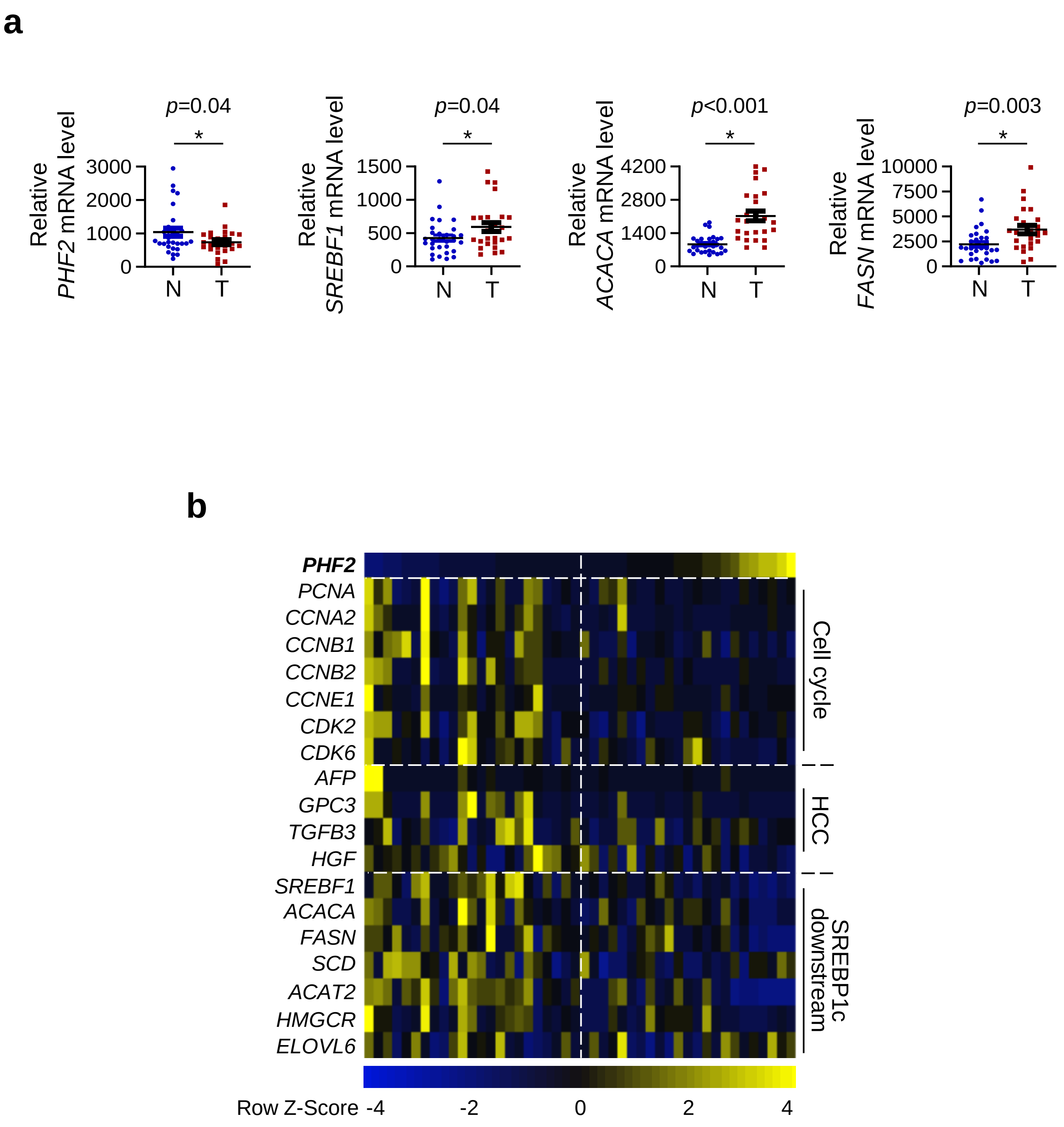

Supplementary Fig. 5: Clinical implications of PHF2 in hepatocellular carcinoma.

a The expressions of *PHF2* and lipogenic genes in HCC tissues and paired non-tumor tissues were analyzed using the GSE54238 dataset. Mean ± SD (n = 56 human liver tissue samples). *P < 0.05. Data were compared using a two-tailed unpaired t-test. T: tumor tissues. N: paired normal liver tissues. **b** Hepatic gene expressions were obtained from the NCBI GEO dataset (GSE54238, n = 56 human liver tissue samples). A heatmap based on *PHF2*-low/high expression values was shown. The color scale bar represents relative expression values ranging from low (blue) to high (yellow).

Supplementary Fig. 6

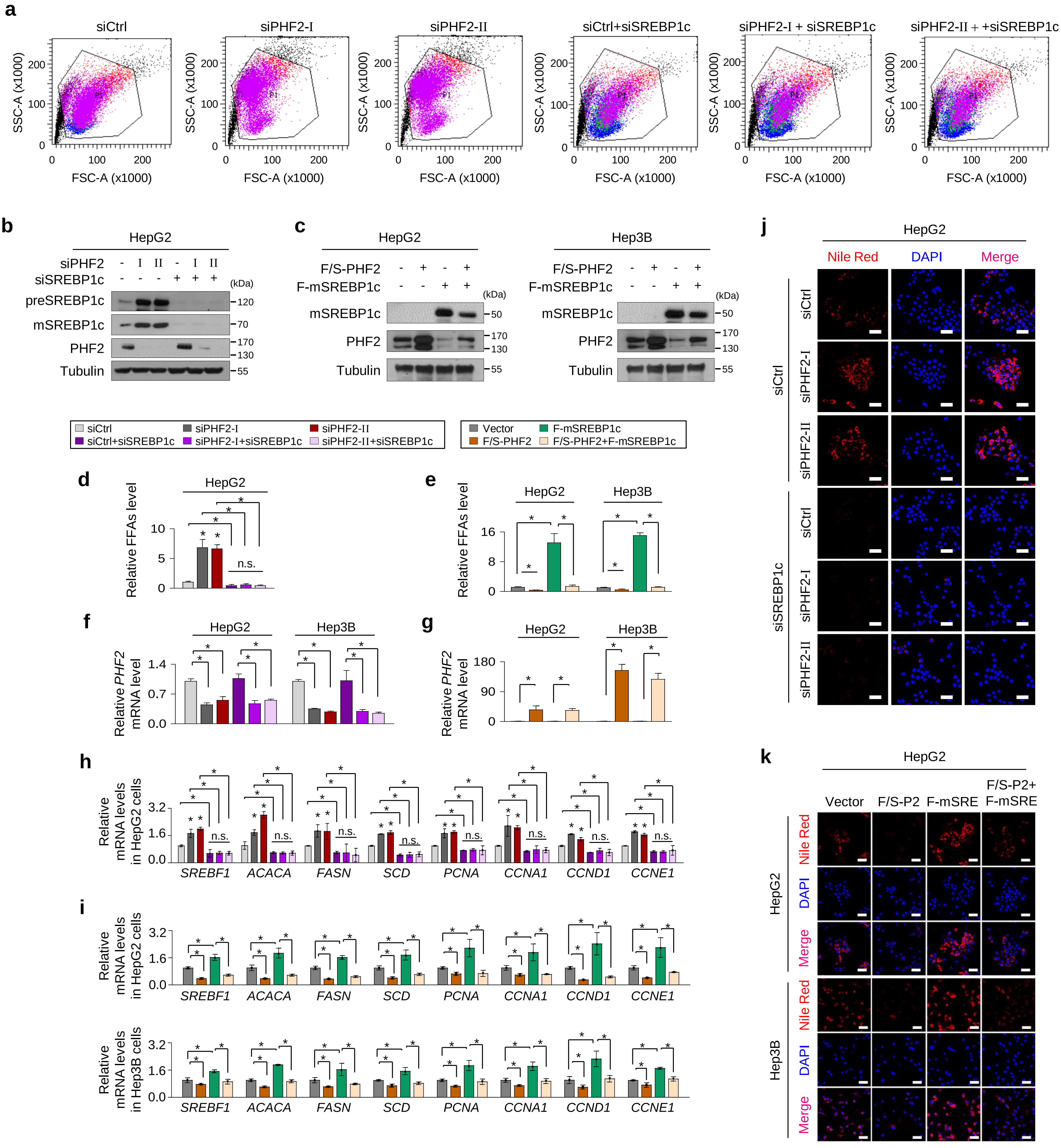

Supplementary Fig. 6: SREBP1c is essential for PHF2 loss-mediated lipogenesis in HCC cells.
a-k HCC cells were transfected with the indicated siRNAs or plasmids. **a** A flow cytometric analysis of Nile Red stained cells was performed (related to Fig. 4e). (**b,c**) Proteins were eluted using a 2X SDS sample buffer and subjected to immunoblotting. (**d,e**) Total free fatty acids (FFAs) levels in cells were measured. Mean ± SD (n = 3 independent samples); *P < 0.05. (**f-i**) RT-qPCR analysis of indicated genes in cells. Gene expression levels were quantified relative to 18S RNA. Mean ± SD (n = 3 independent experiments); *P < 0.05. (**j,k**) HCC cells were fixed with formaldehyde, stained with Nile Red and DAPI, and visualized by fluorescence microscopy. Scale bar = 60 μm. P2: PHF2; SRE: SREBP1c. For the analyses in (**d-i**), an unpaired two-tailed Student's t-test was conducted. Supplementary Fig. **6b-c** and **6j-k** were performed in triplicate. The exact p-values are presented in Supplementary Data 2.

Supplementary Fig. 7

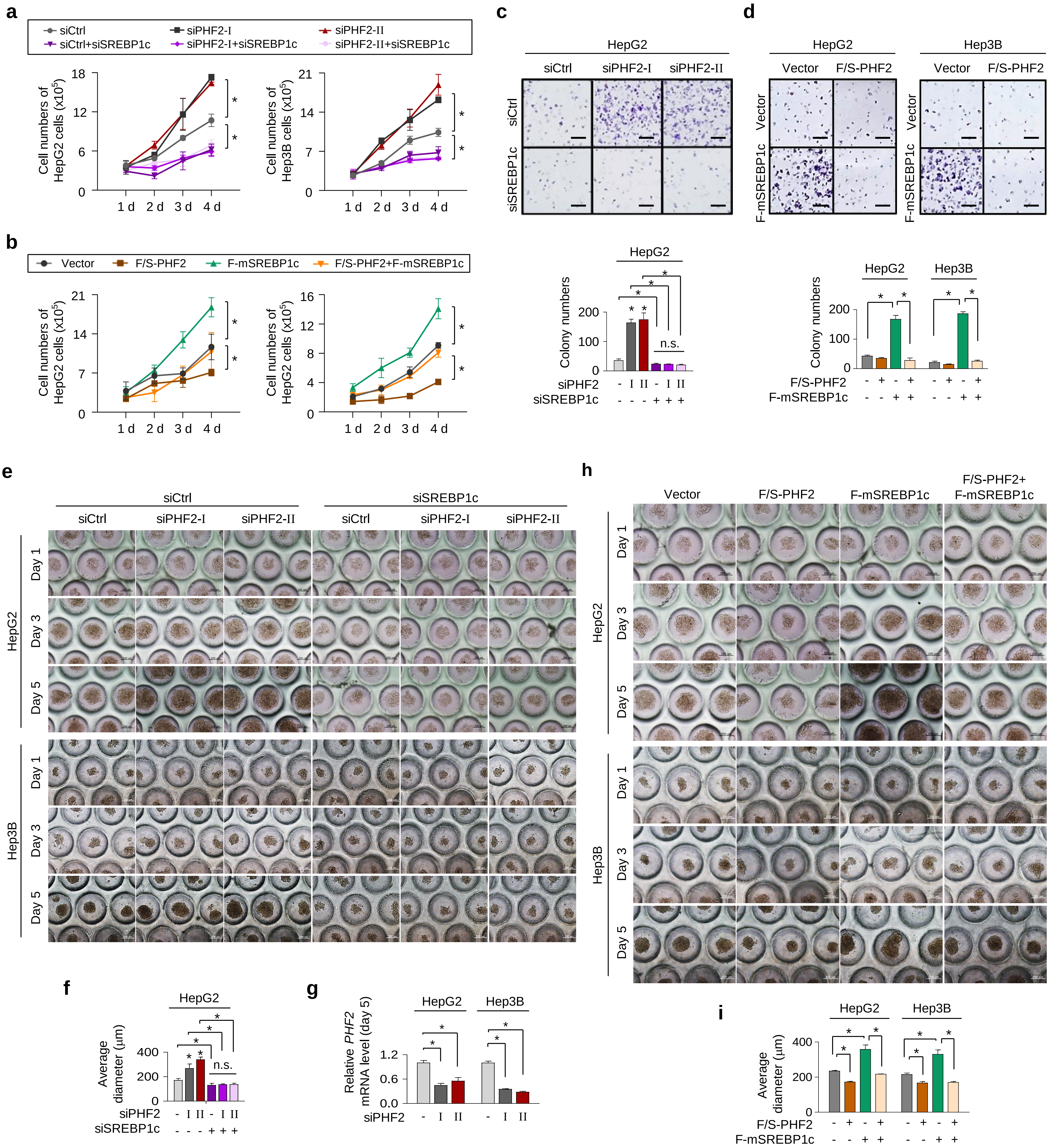

Supplementary Fig. 7: SREBP1c is essential for PHF2 loss-induced proliferation in HCC cells.

a-i HCC cells were transfected with the indicated siRNAs or plasmids. (**a,b**) Cell counting was performed using a hemocytometer 4 d after seeding. Mean ± SD (n = 3 independent samples); *P < 0.05. (**c,d**) HepG2 cells (5 × 10^3 cells/well) and Hep3B cells (1 × 10^4 cells/well) were seeded in 6-well plates. Images were acquired after fixation with methanol and staining with 0.5% crystal violet. Scale bar = 5 mm. Colony numbers were counted after 14 d; mean ± SD (n = 3 independent samples); *P < 0.05. (**e,h**) Full-size images of cells on Oxy chips (related to Fig. 4g). Scale bar = 200 μm. (**f,i**) The average spheroid diameter on Oxy chips was quantified. Mean ± SD (n = 3 independent samples); *P < 0.05. (**g**) The *PHF2* mRNA level on day five was assessed by RT-qPCR. Gene expression level was quantified relative to 18S RNA. Mean ± SD (n = 3 independent experiments); *P < 0.05. For the analyses in (**a-d,f,g,i**), an unpaired two-tailed Student's t-test was conducted. The exact p-values are presented in Supplementary Data 2.

Supplementary Fig. 8

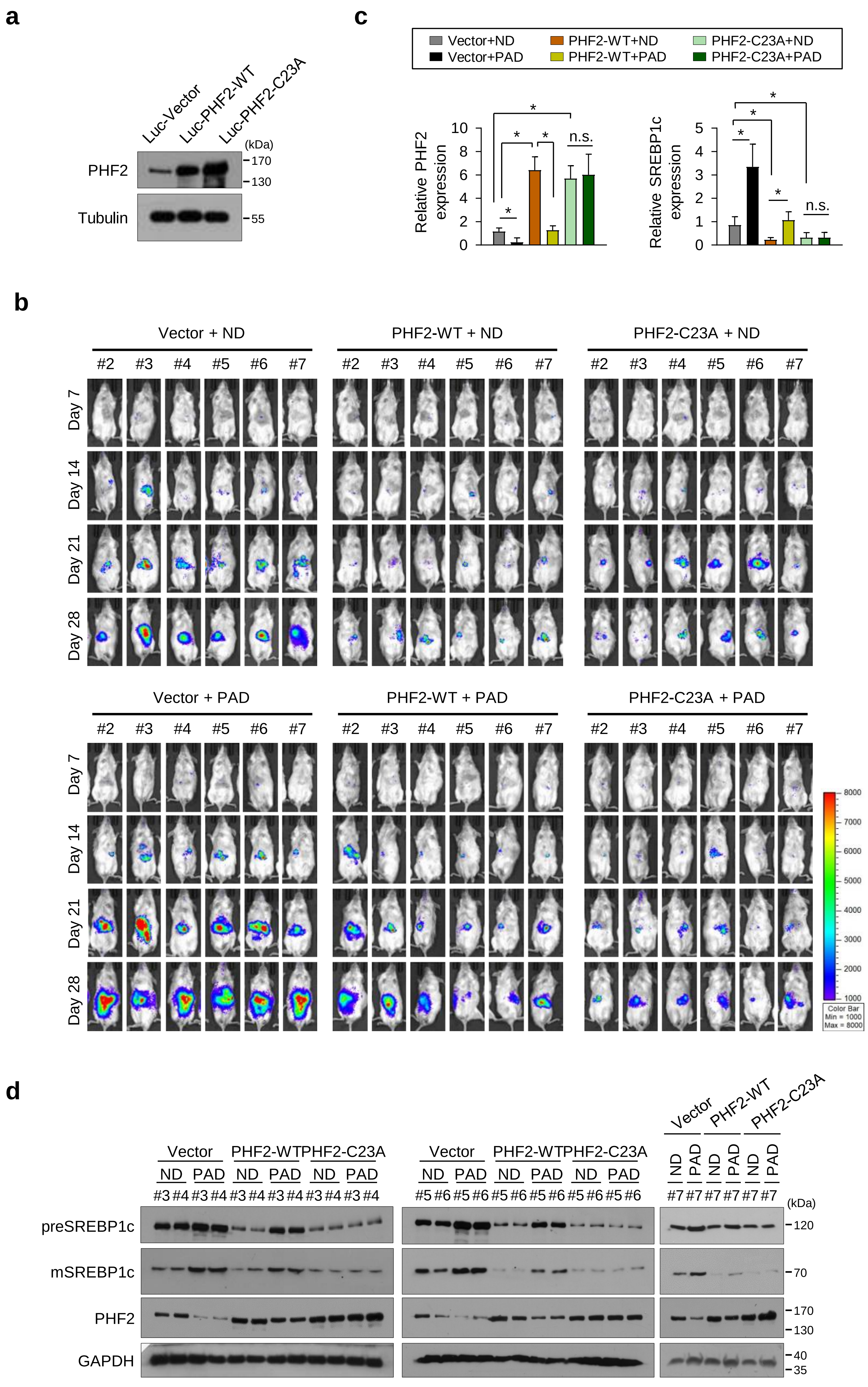

Supplementary Fig. 8: Tumor suppressive role of PHF2 in mice.

a Hep3B cells were transfected with the Luc-IRES-GFP-pcDNA (Luc-Vector), Luc-IRES-GFP-PHF2-WT, or Luc-IRES-GFP-PHF2-C23A plasmid. After cells were selected using G418, the expressions of PHF2 were confirmed using western blotting. **b** Bioluminescence images of mice (related to Fig. 5b). Color scale bars represent luminescence intensity from purple (low) to red (high). ND: normal diet; PAD: palmitic acid-enriched diet. **c** The staining intensity of the indicated antibodies from the immunohistochemical analysis (related to Fig. 5d) was quantified using ImageJ. Mean ± SD (n = 7 independent samples); *P < 0.05 by a two-tailed unpaired t-test. The exact p-values are presented in Supplementary Data 2. **d** The expression levels of the indicated proteins in tumors of mice were analyzed using western blotting. Supplementary Fig. **8a,d** were performed in triplicate.

Supplementary Fig. 9

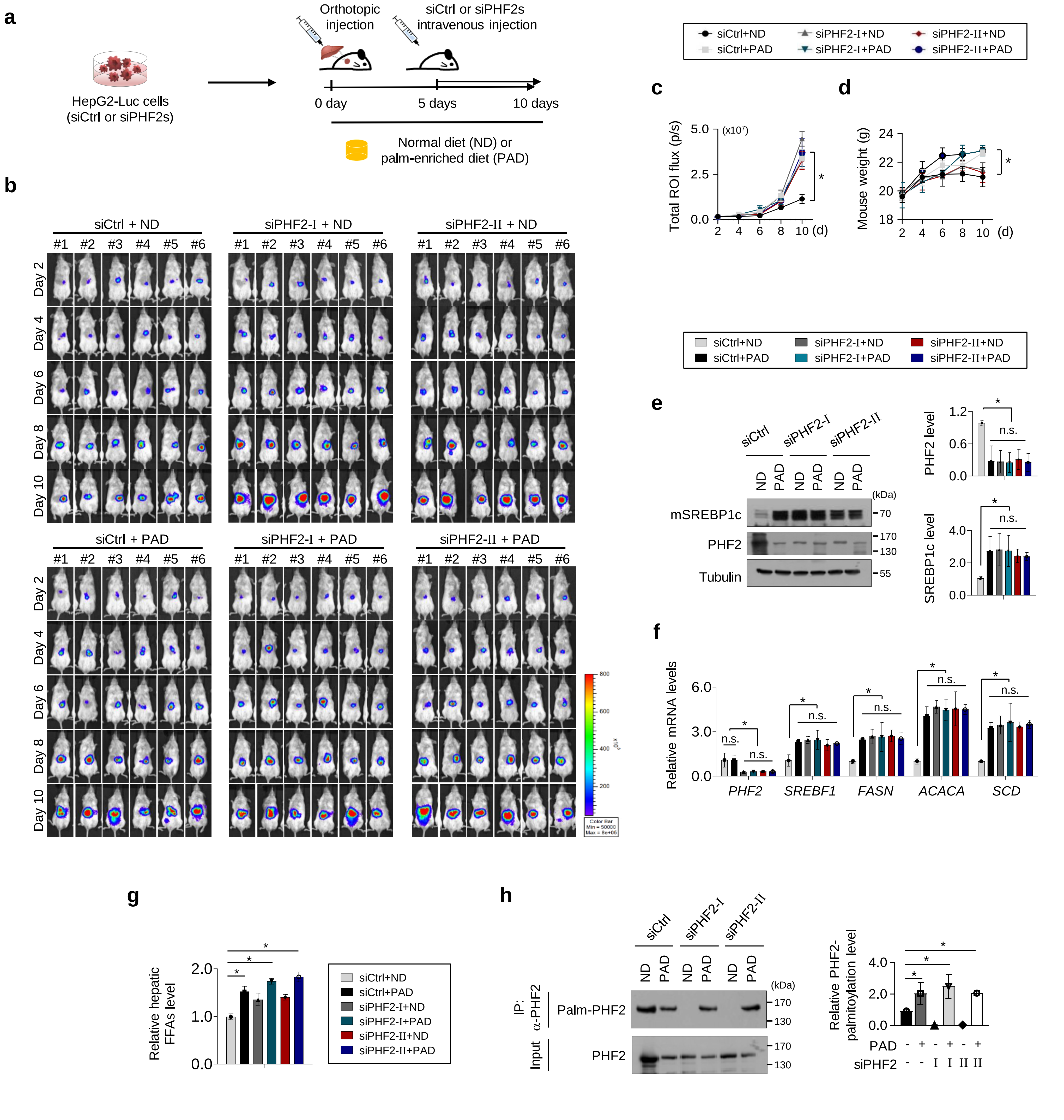

Supplementary Fig. 9: The effects of a palmitic acid-enriched diet and PHF2-loss in xenograft mice.
a Schematic diagram of the *in vivo* model. **b,c** Bioluminescence images of mice obtained using the Xenogen IVIS® Lumina 100. Color scale bars represent luminescence intensity ranging from low (purple) to high (red). Total flux (photons/s/cm^2/sr) was measured and growth curves were plotted based on the bioluminescence intensities. Mean ± SD (n = 6 independent animals for each group); *P < 0.05. **d** Mice were weighed at the indicated times. Mean ± SD (n = 6 independent animals for each group); *P < 0.05. **e,f** Protein expression and mRNA levels were examined in cancer tissues on day 10. The levels were quantified relative to those of tubulin or 18S mRNA, respectively; mean ± SD (n = 3 independent experiments); *P < 0.05. **g** FFAs levels were measured in the hepatic tissues of mice; mean ± SD (n = 3 independent samples); *P < 0.05. **h** An ABE assay was performed to detect PHF2 palmitoylation in cancer tissues. Relative palmitoylation levels were quantified using ImageJ. Mean ± SD (n = 3 independent samples); *P < 0.05. For the analyses in (**c**-**h**), an unpaired two-tailed Student's t-test was conducted. The exact p-values are provided in Supplementary Data 2.

Supplementary Fig. 10

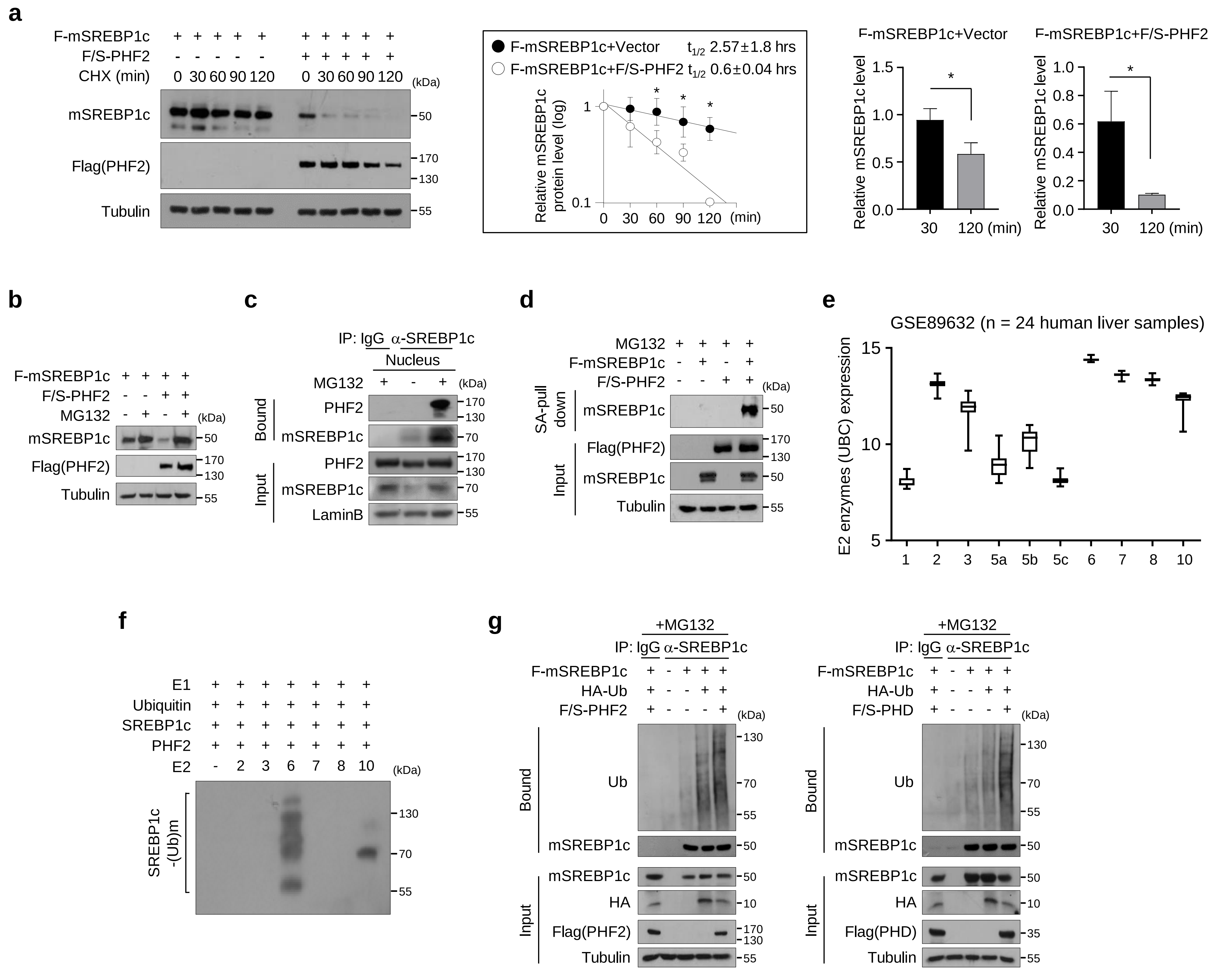

Supplementary Fig. 10: PHF2, as an E3 ubiquitin ligase, degrades SREBP1c.

a (Left) HepG2 cells were transfected with the indicated plasmids and incubated in the presence of cycloheximide for the indicated times. (Middle) Band intensities were quantified using ImageJ. Mean ± SD (n = 3 independent samples); *P < 0.05. (Right) Comparison of signals between 30 and 120 min in the F-mSREBP1c+Vector and between 30 and 120 min in the F-mSREBP1c+F/S-PHF2. Statistical analyses were based on a two-tailed unpaired t-test. The exact p-values are provided in Supplementary Data 2. **b** Protein expression was measured in HepG2 cells transfected with the indicated plasmids and incubated with MG132. Cell lysates were isolated and subjected to western blotting. **c** HepG2 cells were treated with MG132 and subjected to nuclear extraction assays, followed by IP and SDS-PAGE. **d** 293T cells were co-transfected with the indicated plasmids. Proteins were pulled down using SA affinity beads and subjected to SDS-PAGE. **e** mRNA expression of E2 enzymes in the GSE89632 human liver dataset (n = 24 human liver samples). UbcH1 (LMN_3297392), UbcH2 (ILMN_1674633), UbcH3 (ILMN_1713006), UbcH5a (ILMN_1787988), UbcH5b (ILMN_1699503), UbcH5c (ILMN_2241679), UbcH6 (ILMN_1806778), UbcH7 (ILMN_1719039), UbcH8 (ILMN_1707475), and UbcH10 (ILMN_1793651). The box plot of UBC1 represents a maximum of 8.72; upper quartile of 8.23; median of 7.93; lower quartile of 7.88; minimum of 7.69. The box plot of UBC2 represents a maximum of 13.68; upper quartile of 13.28; median of 13.14; lower quartile of 12.99; minimum of 12.38. The box plot of UBC3 represents a maximum of 12.77; upper quartile of 12.22; median of 11.95; lower quartile of 11.62; minimum of 9.66. The box plot of UBC5a represents a maximum of 10.45; upper quartile of 9.26; median of 8.93; lower quartile of 8.35; minimum of 7.98. The box plot of UBC5b represents a maximum of 10.99; upper quartile of 10.66; median of 10.34; lower quartile of 9.54; minimum of 8.75. The box plot of UBC5c represents a maximum of 8.75; upper quartile of 8.23; median of 8.1; lower quartile of 7.98; minimum of 7.82. The box plot of UBC6 represents a maximum of 14.64; upper quartile of 14.43; median of 14.39; lower quartile of 14.32; minimum of 14.26. The box plot of UBC7 represents a maximum of 13.81; upper quartile of 13.66; median of 13.61; lower quartile of 13.54; minimum of 13.27. The box plot of UBC8 represents a maximum of 13.69; upper quartile of 13.43; median of 13.37; lower quartile of 13.25; minimum of 13.07. The box plot of UBC10 represents a maximum of 12.63; upper quartile of 12.59; median of 12.44; lower quartile of 12.13; minimum of 10.66. **f** To identify the E2 enzymes responsible for SREBP1c ubiquitination, an *in vitro* ubiquitination analysis was performed and followed by western blotting. **g** HepG2 cells were co-transfected with the indicated plasmids and incubated with MG132. The cells were incubated with an anti-SREBP1 antibody for 16 h at 4°C, after which the precipitated proteins were subjected to SDS-PAGE. Supplementary Fig. **10b-d** and **10f-g** were performed in triplicate.

Supplementary Fig. 11

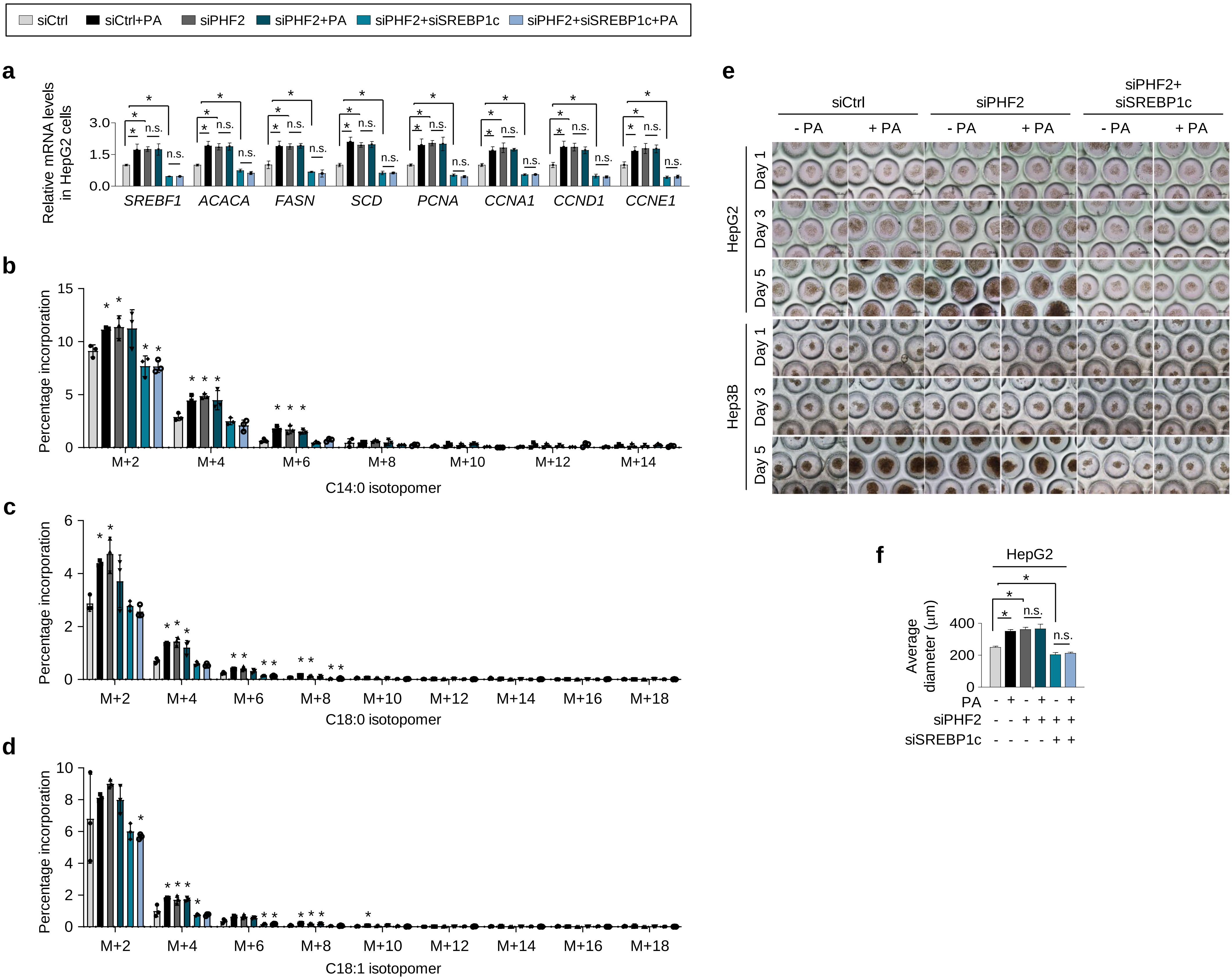

Supplementary Fig. 11: The PA/PHF2/SREBP1c loop rewires lipogenesis and promotes cell proliferation.

a HepG2 cells were transfected with the indicated siRNAs and treated with PA. Gene expression levels related to lipogenesis and proliferation were quantified relative to 18S RNA. Mean ± SD (n = 3 independent samples); *P < 0.05. **b-d** The percent labeling of even isotopomers of indicated metabolites in HepG2 cells treated with ^{13}C-acetate for 24 or 48 h; mean ± SD (n = 3 independent samples); *P < 0.05. **e,f** HCC cells were transfected with the indicated siRNAs, treated with PA, and seeded on 3D chips. Scale bar = 200 µm. (**e**) Full-size images of cells on Oxy chips on 1-5 days (related to Fig. 7e). (**f**) The average diameter of the spheroid was quantified using ImageJ. Mean ± SD (n = 3 independent samples); *P < 0.05. For the analyses in (**a-d,f**), an unpaired two-tailed Student's t-test was conducted. The exact p-values are provided in Supplementary Data 2.

Supplementary Fig. 12

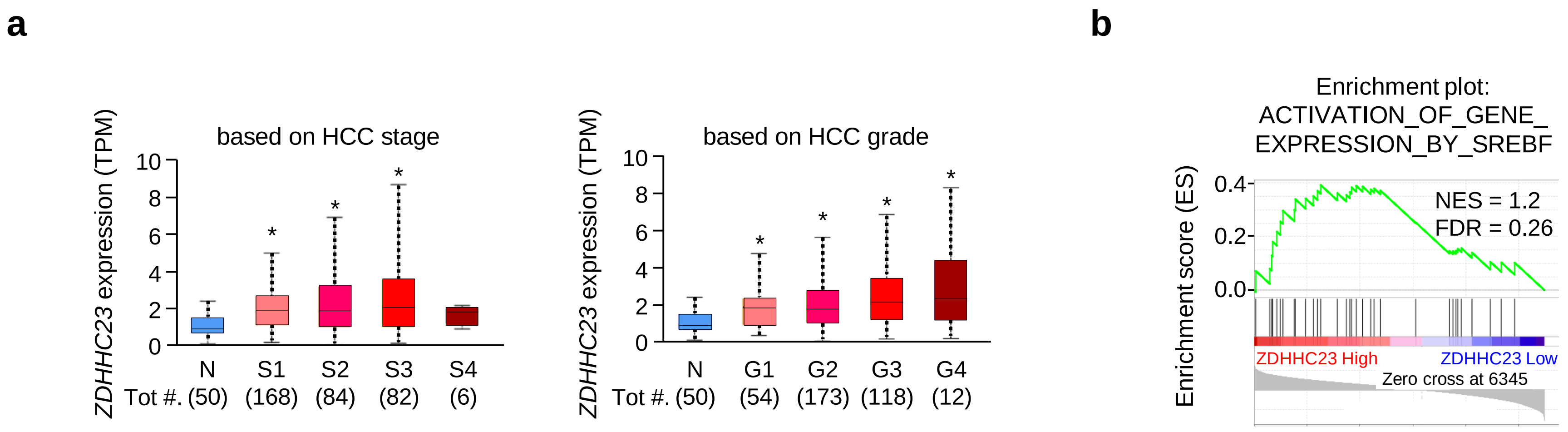

Supplementary Fig. 12: ZDHHC23 is related to HCC progression.

a *ZDHHC23* expression in HCC tissues is shown according to the HCC stage or grade from the UALCAN web resources based on the TCGA datasets. (Left) The box plot of the normal stage (N, n = 50 human liver tissues) represents a maximum of 2.421; upper quartile of 1.467; median of 0.906; lower quartile of 0.711; minimum of 0.101. The box plot of stage 1 (S1, n = 168 human liver tissues) represents a maximum of 5.01; upper quartile of 2.641; median of 1.831; lower quartile of 1.131; minimum of 0.168. The box plot of stage 2 (S2, n = 84 human liver tissues) represents a maximum of 6.914; upper quartile of 3.186; median of 1.862; lower quartile of 1.052; minimum of 0.062. The box plot of stage 3 (S3, n = 82 human liver tissues) represents a maximum of 8.703; upper quartile of 3.539; median of 2.104; lower quartile of 1.054; minimum of 0.165. The box plot of stage 4 (S4, n = 6 human liver tissues) represents a maximum of 2.171; upper quartile of 2.022; median of 1.827; lower quartile of 1.17; minimum of 0.914. (Right) The box plot of normal grade (N, n = 50 human liver tissues) represents a maximum of 2.421; upper quartile of 1.467; median of 0.906; lower quartile of 0.711; minimum of 0.101. The box plot of grade 1 (G1, n = 54 human liver tissues) represents a maximum of 4.724; upper quartile of 2.308; median of 1.849; lower quartile of 0.945; minimum of 0.353. The box plot of grade 2 (G2, n = 173 human liver tissues) represents a maximum of 5.62; upper quartile of 2.694; median of 1.771; lower quartile of 1.094; minimum of 0.062. The box plot of grade 3 (G3, n = 118 human liver tissues) represents a maximum of 6.812; upper quartile of 3.38; median of 2.137; lower quartile of 1.295; minimum of 0.168. The box plot of grade 4 (G4, n = 12 human liver tissues) represents a maximum of 8.251; upper quartile of 4.332; median of 2.343; lower quartile of 1.235; minimum of 0.217. *$P < 0.05$. The exact p-values are provided in Supplementary Data 2. TPM: transcripts per kilobase million; Tot #.: total patients number. **b** Gene expressions were obtained from GSE54238 (n = 56 human liver tissue samples). The GSEA plot for the ACTIVATION_OF_GENE_EXPRESSION_BY_SREBF gene set was shown according to the *ZDHHC23*-high and -low expression groups.

Supplementary Fig. 13

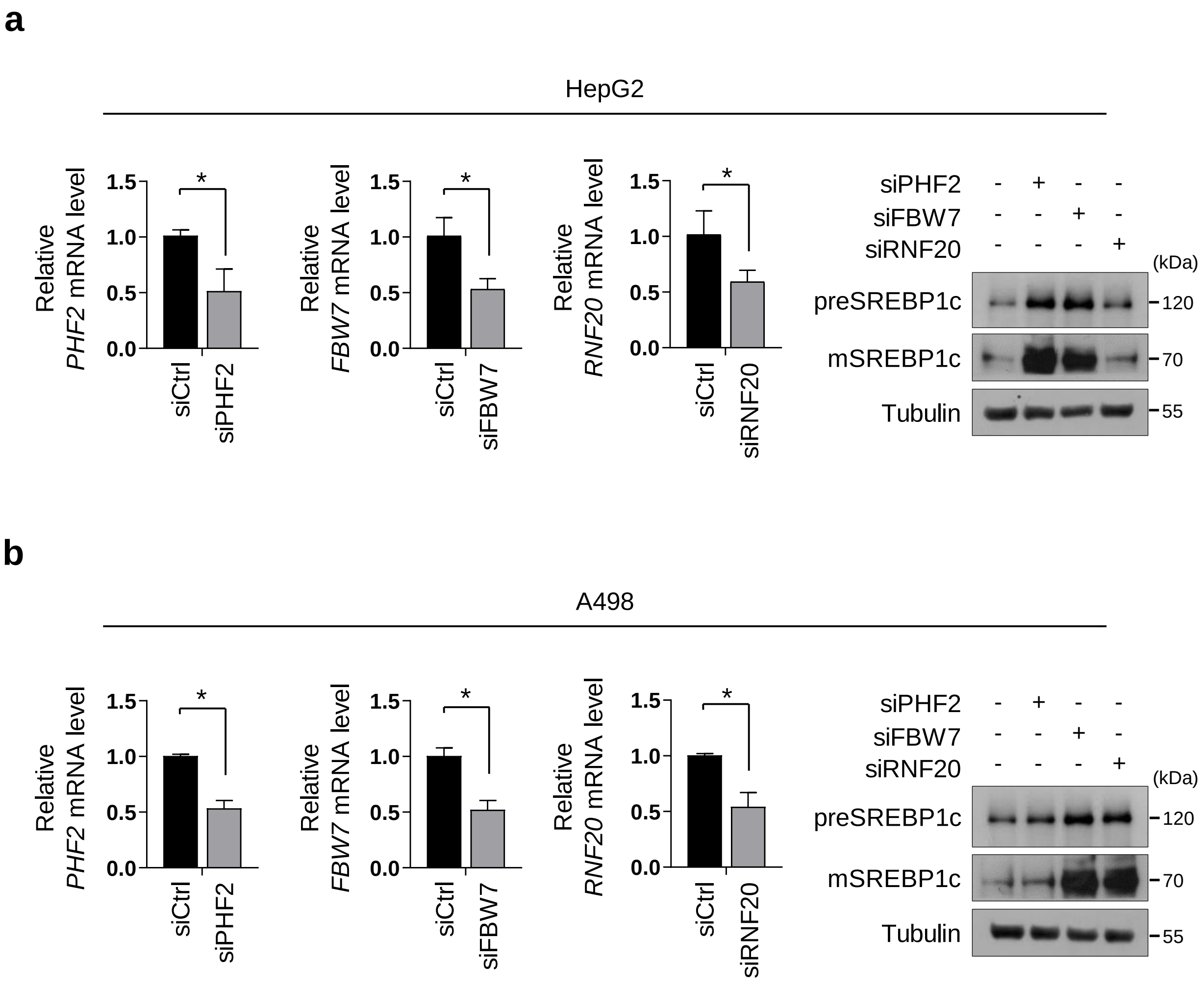

Supplementary Fig. 13: PHF2 is a major E3 ligase of SREBP1c in HCC cells.

a,b mRNA levels and protein expression were examined using RT-qPCR and immunoblotting in lysates from HepG2 and A498 cells transfected with the indicated siRNAs. Expression levels were quantified relative to 18S RNA or tubulin; mean ± SD (n = 3 independent samples); $*P < 0.05$. Statistical analyses were based on a two-tailed unpaired t-test. Supplementary Fig. **13a-b** were performed in triplicate. The exact p-values are provided in Supplementary Data 2.

Supplementary Tables

Supplementary Table 1 Primers used for quantitative RT-PCR and ChIP analyses.

Target gene	Forward primer (5' to 3')	Target gene	Forward primer (5' to 3')
SREBP1c (h)	F-GGAGGGGTAGGGCCAACGGCCT	ZDHHC9	F-ACTTTCCTCGTGGCTCTCAA
	R-CATGTCTTCGAAAGTGCAATCC		R-TCCAGTGGCAAAATACCCCT
ACC (h)	F-GTTGCACAAAAGGATTTCAG	ZDHHC11	F-CTCCCTGCTGATTCACAAGC
	R-CGCATTACCATGCTCCGCAC		R-CATCTGCTTCCTGTGCCATC
FAS (h)	F-ACAGGGACAACCTGGAGTTCT	ZDHHC12	F-GGATGGAGAACTGTGTGGGA
	R-CTGTGGTCCCACTTGATGAGT		R-ACCAACGAGAAGAGGGACAG
SCD1 (h)	F-TGGGTGGCTGCTTGTG	ZDHHC13	F-AGACTTGGGCAACTGATCCA
	R-GCGTGGGCAGGATGAAG		R-GCATACATGGCAGTGGAGTG
PHF2 (h)	F-CCTGCTGGAGGCATTCAAAG	ZDHHC14	F-AGAAGAAGAAAATCGCGGCC
	R-CACGATCGGAAAGCACCATT		R-GGATGGCAGGGGTGATTTTC
PCNA (h)	F-CATGGGCGTGAACCTCACC	ZDHHC15	F-CCAGTGTTTACAAGTGGCCC
	R-CACAGCTGTACTCCTGTTCTGG		R-ATCCTCGTTGTCTTCCCAGG
CyclinA (h)	F-CCTTAGGGAAATGGAGGTTAAA	ZDHHC16	F-TTATCACCAGACCCCACCAC
	R-CCAAATGCAGGGTCTCATTC		R-TCCAAGCAGCCGTAGTTGTA
CyclinD (h)	F-TTCCTCTCCAAAATGCCAGA	ZDHHC17	F-AGCGGGAGGAGGGATTTAAC
	R-CAGTCCGGGTCACACTTGAT		R-CCTGCTTCCACCAATTCTCG
CyclinE (h)	F-TCAGTGGTGCGACATAGAGAA	ZDHHC18	F-TTCCTGACGGCCTTCATCTT
	R-TGTCCAGCAAATCCAAGCTG		R-GACCACGAGCCTTTGATGTC
18s	F-TTCGTATTGAGCCGCTAGA	ZDHHC19	F-ATACAACCCCTTCGACCAGG
	R-CTTTCGCTCTGGTCCGTCTT		R-TGCTTTGTAGGGACCCAGAG
ZDHHC1	F-CTCCCTGCTGATTCACAAGC	ZDHHC20	F-GCGTCCTCTCACTTTTCAGC
	R-CATCTGCTTCCTGTGCCATC		R-CCACAAGGCGAGTTGGAAAA
ZDHHC2	F-TCTTAGGCGAGCAGCCAAGGAT	ZDHHC21	F-GAGAACCCCAAGATCCCACA
	R-CAGTGATGGCAGCGATCTGGTT		R-CACAACTGCAGAAAGAGCCA
ZDHHC3	F-TCTTAGGCGAGCAGCCAAGGAT	ZDHHC22	F-CACTGTTTCTTCACCGGCAA
	R-GCATTTGGGGCACTTGTACA		R-TCCGACATTGAACATGGGGA
ZDHHC4	F-CGGATTGTCTTCATGCTGGG	ZDHHC23	F-GCGCGGGGAGGCGGGCGCGC
	R-AGTGAATGTTCCGGTGGACT		R-CAGTTGCACCTCCGGCCAGA
ZDHHC5	F-ATCCATTCGTTCAGAGGGCA	ZDHHC24	F-CTCTTCCATGGGATGCTGCT
	R-TAAAGGTGGGTCTGGCTCAG		R-ATCTGCTGTGGTCTGGAAGG
ZDHHC6	F-GCCTCGAATACAGCTGCAAA	FAS_ChIP_P1 (h)	F-CCCTGAACTGAATGGGTCAG
	R-TCAGCATCACAGGGACACTT		R-CTTCCTCATGTGGCCAGTTC
ZDHHC7	F-CTTCATCGTCCTCCTCCTCC	FAS_ChIP_P2 (h)	F-AGACGGGACGGAGATGTTAGT
	R-GGTTCTCAGGTGGGATGACA		R-TCCCCTCTGTTTCTCCTTCTC
ZDHHC8	F-TTCATCCCTGTCATTGGCCT	SCD1_ChIP_P1 (h)	F-CAGGCGCGTTCCCGCGCAGG
	R-GAAAGGCGGCTTCAAACTCA		R-GAGAGCGAGGCTGGAGCGCG
		SCD1_ChIP_P2 (h)	F-GAGAAGGAGAAACAGAGGGGA
			R-CCGACACCACACCACCCGGCC

Supplementary Table 2 Oligo sequences for si-RNAs.

Target gene	Sense (5’-3’)
Control	UUGAGCAAUUCACGUUCAUTT
PHF2 I (h, m)	GAAGAAGCAGGCUUUGGCAGAGCAT
PHF2 II (h, m)	AAACCUGACUCCUUACUGAAGAUGG
SREBP1c (h)	GGAAGAGUCAGUGCCACUGAGCATC
ZDHHC1 (h)	ACCGGCUGUGAUGCUCCAAUAAACT
ZDHHC2 (h)	CAAGCCAAGUUCCAUAUUAUGUUTT
ZDHHC3 (h)	UGGUCCUCUAUGCGGAGUUCGUGGT
ZDHHC4 (h)	CGGAUUGUCUUCAUGCUGGGCUUTG
ZDHHC5 (h)	AUCCAUUCGUUCAGAGGGCACCACC
ZDHHC6 (h)	GCCUCGAAUACAGCUGCAAAAAGGG
ZDHHC7 (h)	CCGGGACGUCGAGCAUCAUCCUCTC
ZDHHC8 (h)	UUCAUCCCUGUCAUUGGCCUCACTG
ZDHHC9 (h)	ACUUUCCUCGUGGCUCUCAACCAGA
ZDHHC11 (h)	CUCCCUGCUGAUUCACAAGCACUTA
ZDHHC12 (h)	GGAUGGAGAACUGUGUGGGAGAGCG
ZDHHC13 (h)	AGACUUGGGCAACUGAUCCAGGCTT
ZDHHC14 (h)	CAAGCCUGAUCGACAGAAGAGGGTA
ZDHHC15 (h)	CCAGUGUUUACAAGUGGCCCAGAGA
ZDHHC16 (h)	UUAUCACCAGACCCCACCACCCACC
ZDHHC17 (h)	AGCGGGAGGAGGGAUUUAACACCAA
ZDHHC18 (h)	CCGGCCUCUUCUUCGUCUUUGACTG
ZDHHC19 (h)	AUACAACCCCUUCGACCAGGGCUGT
ZDHHC20 (h)	GCGUCCUCUCACUUUUCAGCUACCA
ZDHHC21 (h)	GAGAACCCCAAGAUCCCACAUGGAG
ZDHHC22 (h)	CACUGUUUCUUCACCGGCAACUGCA
ZDHHC23 (h)	GGGAUCACACUGACCUUGGACACCA
ZDHHC24 (h)	CUCUUCCAUGGGAUGCUGCUGCUGC
RNF20	GGAUAAAGAGAAAGGCAAA
FBX7	GTGTGGAATGCAGAGACTGGAGA

Supplementary Table 3 Diet composition of the palmitic acid-enriched diet.

	gram	Kcal
Protein	24	20
Carbohydrates	41	35
Fat	24	45
Total	89	100
Calories per gram	4.73	
Fat source		
Soybean oil	19	171
Palm oil	183.5	1652
Ingredients		
Casein	200	800
L-cysteine	3	12
Corn starch	72.8	291
Maltodextrin 10	100	400
Sucrose	172.8	691
Cellulose	50	0
Mineral mix S 10026	10	0
Dicalcium phosphate	13	0
Calcium carbonate	5.5	0
Potassium citrate, 1 H_2O	16.5	0
Vitamin mix V 10001	10	40
Choline bitartrate	2	0
Blue Dye #1, FD&C	0.05	0
Total	858.15	4057

Supplementary Table 4 Patients' information for hepatocellular carcinoma for the RT-qPCR analysis.

No.	age	sex	Diagnosis	Normal tissue	Tumor tissue
1	age ≤ 40	Male	Hepatocellular carcinoma	1	1
2		Female	Hepatocellular carcinoma	1	1
3	50 ≤ age < 60	Male	Hepatocellular carcinoma	1	1
4		Male	Hepatocellular carcinoma	1	1
5		Female	Hepatocellular carcinoma	1	1
6		Male	Hepatocellular carcinoma	1	1
7		Male	Hepatocellular carcinoma	1	1
8		Male	Hepatocellular carcinoma	1	1
9		Male	Hepatocellular carcinoma	1	1
10		Male	Hepatocellular carcinoma	1	1
11		Male	Hepatocellular carcinoma	1	1
12	60 ≤ age < 70	Male	Hepatocellular carcinoma	1	1
13		Male	Hepatocellular carcinoma	1	1
14		Male	Hepatocellular carcinoma	1	1
15		Male	Hepatocellular carcinoma	1	1
16		Male	Hepatocellular carcinoma	1	1
17		Male	Hepatocellular carcinoma	1	1
18		Male	Hepatocellular carcinoma	1	1
19		Male	Hepatocellular carcinoma	1	1
20		Male	Hepatocellular carcinoma	1	1
21		Male	Hepatocellular carcinoma	1	1
22		Male	Hepatocellular carcinoma	1	1
23		Female	Hepatocellular carcinoma	1	1
24	70 ≤ age < 80	Male	Hepatocellular carcinoma	1	1
25		Male	Hepatocellular carcinoma	1	1
26		Male	Hepatocellular carcinoma	1	1
27		Male	Hepatocellular carcinoma	1	1
28		Male	Hepatocellular carcinoma	1	1
29		Male	Hepatocellular carcinoma	1	1
30	80	Male	Hepatocellular carcinoma	1	1

Supplementary Table 5 Patients' information on follow-up months and survival in the cancer tissue array.

No.	age	sex	Diagnosis	Follow-up months	status
1	age ≤ 40	Male	Hepatocellular carcinoma	55	dead
2		Male	Hepatocellular carcinoma	31	alive
3		Male	Hepatocellular carcinoma	28	dead
4	40 ≤ age < 50	Female	Hepatocellular carcinoma	53	dead
5		Male	Hepatocellular carcinoma	130	alive
6		Male	Hepatocellular carcinoma	11	dead
7		Male	Hepatocellular carcinoma	35	dead
8		Male	Hepatocellular carcinoma	126	alive
9		Male	Hepatocellular carcinoma	29	dead
10	50 ≤ age < 60	Male	Hepatocellular carcinoma	23	dead
11		Male	Hepatocellular carcinoma	68	dead
12		Male	Hepatocellular carcinoma	35	alive
13		Male	Hepatocellular carcinoma	31	dead
14		Male	Normal (match of #28)	unknown	unknown
15		Male	Hepatocellular carcinoma	126	alive
16		Male	Normal (match of #6)	unknown	unknown
17		Male	Hepatocellular carcinoma	121	alive
18		Male	Hepatocellular carcinoma	65	alive
19		Male	Hepatocellular carcinoma	38	dead
20		Male	Hepatocellular carcinoma	36	dead
21		Male	Normal (match of #13)	unknown	unknown
22		Male	Hepatocellular carcinoma	47	alive
23		Male	Hepatocellular carcinoma	93	dead
24		Male	Hepatocellular carcinoma	34	dead
25	60 ≤ age < 70	Male	Hepatocellular carcinoma	29	alive
26		Male	Hepatocellular carcinoma	unknown	unknown
27		Male	Normal (match of #3)	unknown	unknown
28		Male	Hepatocellular carcinoma	132	alive
29		Male	Normal (match of #1)	unknown	unknown
30		Male	Hepatocellular carcinoma	5	alive
31		Male	Hepatocellular carcinoma	76	dead
32		Male	Hepatocellular carcinoma	39	dead
33		Male	Hepatocellular carcinoma	54	alive
34		Male	Normal (match of #2)	unknown	unknown
35		Male	Hepatocellular carcinoma	12	dead
36		Male	Normal (match of #4)	unknown	unknown
37		Male	Hepatocellular carcinoma	96	alive
38		Male	Hepatocellular carcinoma	54	alive
39		Female	Hepatocellular carcinoma	130	alive
40		Male	Hepatocellular carcinoma	127	alive
41		Male	Normal (match of #9)	unknown	unknown
42	70 ≤ age < 80	Male	Hepatocellular carcinoma	80	dead
43		Male	Hepatocellular carcinoma	10	dead
44		Male	Normal (match of #7)	unknown	unknown
45		Male	Hepatocellular carcinoma	8	dead
46		Male	Hepatocellular carcinoma	3	dead
47		Male	Hepatocellular carcinoma	44	alive

Supplementary Table 6 The list of antibodies for immunoblotting.

Antibodies	Source	Identifier	Blocking buffer	Dilution
Mouse monoclonal anti-SREBP1	BD Biosciences	557036	3% BSA	1:500
Mouse monoclonal anti-Ubiquitin	Santa Cruz	sc-9133	1% BSA + 1% skim milk	1:1,000
Rabbit polyclonal anti-PHF2	Cell Signaling	D45A2	5% skim milk	1:1,000
Mouse monoclonal anti-Flag	Sigma-Aldrich	F3165	5% skim milk	1:1,000
Mouse monoclonal anti-Tubulin	Cell Signaling	2146S	1% BSA + 1% skim milk	1:10,000
Rabbit polyclonal anti-ZDHHC23	Sigma-Aldrich	HPA016808	3% BSA	1:1,000
Rabbit polyclonal anti-HA	Santa Cruz	sc-805	5% skim milk	1:1,000
Mouse monoclonal anti-Myc	Cell Signaling	2276s	3% BSA	1:1,000
Rabbit polyclonal anti-Myc	Cell Signaling	2278s	3% BSA	1:1,000
Mouse monoclonal anti-Lamin B	Santa Cruz	sc-374015	5% skim milk	1:1,000
Mouse monoclonal anti-His-tag	MBL	D291-3	5% skim milk	1:1,000

* BSA: bovine serum albumin
