# Supplementary material for: Palmitoylation-driven PHF2 ubiquitination remodels lipid metabolism through the SREBP1c axis in hepatocellular carcinoma
Source: Nat Commun. 2023 Oct 12;14:6370. doi: 10.1038/s41467-023-42170-0 (PMC10570296; doi:10.1038/s41467-023-42170-0)
Supplement: Supplementary file 2 — Description of Additional Supplementary Information [file 41467_2023_42170_MOESM2_ESM.pdf]

Description of Additional Supplementary Information

File name: Supplementary Data 1
Description: PHF2-interacting proteins detected using LC-MS in HepG2 cells.

File name: Supplementary Data 2
Description: The exact p-values of main and supplementary figures.
