# Supplementary material for: Palmitoylation-driven PHF2 ubiquitination remodels lipid metabolism through the SREBP1c axis in hepatocellular carcinoma
Source: Nat Commun. 2023 Oct 12;14:6370. doi: 10.1038/s41467-023-42170-0 (PMC10570296; doi:10.1038/s41467-023-42170-0)
Supplement: Supplementary file 5 — Reporting Summary [file 41467_2023_42170_MOESM5_ESM.pdf]

nature portfolio

Corresponding author(s): Yang-Sook Chun

Last updated by author(s): Jun 26, 2023

Reporting Summary

Nature Portfolio wishes to improve the reproducibility of the work that we publish. This form provides structure for consistency and transparency in reporting. For further information on Nature Portfolio policies, see our Editorial Policies and the Editorial Policy Checklist.

Statistics

For all statistical analyses, confirm that the following items are present in the figure legend, table legend, main text, or Methods section.

n/a	Confirmed	
☐	☒	The exact sample size (*n*) for each experimental group/condition, given as a discrete number and unit of measurement
☐	☒	A statement on whether measurements were taken from distinct samples or whether the same sample was measured repeatedly
☐	☒	The statistical test(s) used AND whether they are one- or two-sided<br>*Only common tests should be described solely by name; describe more complex techniques in the Methods section.*
☐	☒	A description of all covariates tested
☐	☒	A description of any assumptions or corrections, such as tests of normality and adjustment for multiple comparisons
☐	☒	A full description of the statistical parameters including central tendency (e.g. means) or other basic estimates (e.g. regression coefficient) AND variation (e.g. standard deviation) or associated estimates of uncertainty (e.g. confidence intervals)
☐	☒	For null hypothesis testing, the test statistic (e.g. *F*, *t*, *r*) with confidence intervals, effect sizes, degrees of freedom and *P* value noted<br>*Give P values as exact values whenever suitable.*
☒	☐	For Bayesian analysis, information on the choice of priors and Markov chain Monte Carlo settings
☒	☐	For hierarchical and complex designs, identification of the appropriate level for tests and full reporting of outcomes
☐	☒	Estimates of effect sizes (e.g. Cohen's *d*, Pearson's *r*), indicating how they were calculated

Our web collection on statistics for biologists contains articles on many of the points above.

Software and code

Policy information about availability of computer code

Data collection	Immunofluorescence images were observed using a confocal microscope (FV3000; Olympus, Tokyo, Japan).<br>Fluorescence from in vitro palmitoylation assay was detected using the Gel-Doc EQ system (BIO-RAD, Hercules, CA).<br>The initial 3D structure of PHF2 (a.a. 1-444) was constructed using the SWISS-MODEL homology modelling server (PMID: 29788355).<br>docking simulations were performed using ZDOCK server (PMID: 21949741).<br>NPT-ensemble MD simulations with the distant restraint were carried out using NAMD program (PMID: 16222654).<br>The bonds involving hydrogen were constrained to be rigid by using the SHAKE algorithm (DOI: 10.1016/0021-9991(77)90098-5).<br>The LC/MS spectra for analysis of PHF2-interacting proteins were searched SEQUEST software in Proteome Discoverer 1.4 (Ver. 1.4.0.288).<br>Flow cytometric analysis was performed using a flow cytometer (FACSymphony A5, BD Biosciences).<br>GC-TOF/MS analysis was performed using an Agilent 7890B GC system (Agilent, Santa Clara, CA, USA) coupled with a LECO Pegasus HT TOF/MS device (LECO, St. Joseph, MI, USA).<br>Bioluminescence images of mice were obtained using the Xenogen IVIS® Lumina in vivo imaging technology platform (Xenogen, Alameda, CA).<br>The human tissue slide was photographed using a TMA scanner (Leica, APERIO AT1).

Data analysis

The LC-MS analyses for quantification of palmitoyl coenzyme A and 13C-labeled acetate were processed using QuantLynx software (Ver 4.2, Waters, Milford, MA, USA).
The protein-protein networks were visualized using the STRING App within Cytoscape (Ver 3.8.0).
RT-qPCR value, Pearson's correlation coefficient value and Kaplan–Meier analysis of the survival rates were analyzed using GraphPad Prism (Ver. 8.0.2).
ImageJ program (Ver. 1.52a) was used to analyze average spheroid diameter from 3D culture, immunofluorescence, immunohistochemistry, and western blots.

For manuscripts utilizing custom algorithms or software that are central to the research but not yet described in published literature, software must be made available to editors and reviewers. We strongly encourage code deposition in a community repository (e.g. GitHub). See the Nature Portfolio guidelines for submitting code & software for further information.

Data

Policy information about availability of data

All manuscripts must include a data availability statement. This statement should provide the following information, where applicable:

- Accession codes, unique identifiers, or web links for publicly available datasets
- A description of any restrictions on data availability
- For clinical datasets or third party data, please ensure that the statement adheres to our policy

Structural difference between wild-type and mutant residues of protein (PDB file 3kqi) was predicted using Dynamut (http://biosig.unimelb.edu.au/dynamut/).
PHF2-interacting proteins obtained from LC-MS were annotated using DAVID Bioinformatics Resources (https://david.ncifcrf.gov/chartReport.jsp?d-16544-p=1&d-16544-o=1&annot=85&d-16544-s=5).
The public NCBI dataset were acquired under accession code GSE54238 (https://www.ncbi.nlm.nih.gov/geo/query/acc.cgi?acc=GSE54238) and GSE89632 (https://www.ncbi.nlm.nih.gov/geo/query/acc.cgi?acc=GSE89632).
Arraystar human lncRNA microarray GPL16955 (https://www.ncbi.nlm.nih.gov/geo/query/acc.cgi?acc=GPL16955) and GPL14951 (https://www.ncbi.nlm.nih.gov/geo/query/acc.cgi?acc=GPL14951) platforms were obtained from GEO public database.
Publicly available databases from UALCAN (https://ualcan.path.uab.edu/) were used for analyzing ZDHHC23 expression.

Human research participants

Policy information about studies involving human research participants and Sex and Gender in Research.

Reporting on sex and gender

A total of 30 HCC tissues and the corresponding adjacent non-cancerous specimens were obtained from the tissue bank of Seoul National University Hospital (Seoul, South Korea) with consent under approval from the Institutional Review Board of Seoul National University Hospital (approval No. C-1908-039-1053). Although samples contained 27 male and 3 female patients' tissues, sex and/or gender were not considered in the study design to avoid bias induced by sex and/or gender.

Population characteristics

A total of 30 cancer tissues and the adjacent non-cancerous tissues were obtained from patients with hepatocellular carcinoma. Tissues were stored at the tissue bank of Seoul National University Hospital (Seoul, South Korea) under approval from the Institutional Review Board of Seoul National University Hospital (approval No. C-1908-039-1053). Detailed patient information including sex and age is presented in Supplementary Table 4.

Recruitment

Patients diagnosed with hepatocellular carcinoma were randomly recruited and stored under authorization by the Institutional Review Board of Seoul National University Hospital (approval No. C-1908-039-1053). There is no selection bias in the recruitment.

Ethics oversight

To analyze HCC tissues, this study was additionally approved by the Institutional Review Board of Seoul National University Hospital (approval No. C-1908-039-1053). Because it was minimal-risk research, no informed consent by the participants was needed.

Note that full information on the approval of the study protocol must also be provided in the manuscript.

Field-specific reporting

Please select the one below that is the best fit for your research. If you are not sure, read the appropriate sections before making your selection.

☒ Life sciences ☐ Behavioural & social sciences ☐ Ecological, evolutionary & environmental sciences

For a reference copy of the document with all sections, see nature.com/documents/nr-reporting-summary-flat.pdf

Life sciences study design

All studies must disclose on these points even when the disclosure is negative.

Sample size

No statistical methods were used to pre-determine the sample size. Sample sizes are based on several factors including the purpose of the experiment, the available resources, and previous research in the field. The sample size of each experiment is provided in the figure legends in

the manuscript and supplementary files. For in vivo mouse experiments, mouse number >=6 was used. Published papers were used as references (PMID: 33128030, 32332706, 34179825, 30291252, 26900866)

Data exclusions	No data were excluded and all samples were included in the data analysis.
Replication	All experiments were experimental triplicates repeated with similar results as stated in the Figure Legends section. For the animal study, each group has at least six mice.
Randomization	For all animal studies, animals in each group of the same gender, age, and genetic background were assigned randomly to experimental and control groups. For other experiments, randomization was not required because all samples were analyzed with the same indicated methods.
Blinding	All the control and experimental groups of mice/cells were grown under identical conditions. The investigators were blinded to group allocation during data collection and/or analysis and the data were processed and analyzed by the blinded co-author.

Reporting for specific materials, systems and methods

We require information from authors about some types of materials, experimental systems and methods used in many studies. Here, indicate whether each material, system or method listed is relevant to your study. If you are not sure if a list item applies to your research, read the appropriate section before selecting a response.

Materials & experimental systems

n/a	Involved in the study
☐	☒ Antibodies
☐	☒ Eukaryotic cell lines
☒	☐ Palaeontology and archaeology
☐	☒ Animals and other organisms
☒	☐ Clinical data
☒	☐ Dual use research of concern

Methods

n/a	Involved in the study
☒	☐ ChIP-seq
☒	☐ Flow cytometry
☒	☐ MRI-based neuroimaging

Antibodies

Antibodies used

For immunoblotting (primary antibodies):
Mouse monoclonal anti-SREBP1 (BD Biosciences, 557036, 1:500 in 3% BSA)
Mouse monoclonal anti-Ubiquitin (Santa Cruz, sc-9133, 1:1,000 in 1% BSA + 1% skim milk)
Rabbit polyclonal anti-PHF2 (Cell Signaling, D45A2, 1:1,000 in 5% skim milk)
Mouse monoclonal anti-Flag (Sigma-Aldrich, F3165, 1:1,000 in 5% skim milk)
Mouse monoclonal anti-Tubulin (Cell Signaling, 2146S, 1:10,000 in 1% BSA + 1% skim milk)
Rabbit polyclonal anti-ZDHHC23 (Sigma-Aldrich, HPA016808, 1:1,000 in 3% BSA)
Rabbit polyclonal anti-HA (Santa Cruz, sc-805, 1:1,000 in 5% skim milk)
Mouse monoclonal anti-Myc (Cell Signaling, 2276s, 1:1,000 in 3% BSA)
Rabbit polyclonal anti-Myc (Cell Signaling, 2278s, 1:1,000 in 3% BSA)
Mouse monoclonal anti-Lamin B (Santa Cruz, sc-374015, 1:1,000 in 5% skim milk)
Mouse monoclonal anti-His-tag (MBL, D291-3, 1:1,000 in 5% skim milk)

For immunoblotting (secondary antibodies):
Goat anti-mouse igG horseradish peroxidase conjugate (Invitrogen, G21040, 1:5,000 in the blocking buffer)
Goat anti rabbit IgG horse radish peroxide conjugate (Invitrogen, G21234, 1:5,000 in the blocking buffer)

For immunofluorescence (primary antibodies):
Goat polyclonal anti-PHF2 (Santa Cruz, sc-324199, 1:250 in PBS containing 3% BSA and 0.3% triton X-100)
Rabbit polyclonal anti-ZDHHC23 (Sigma-Aldrich, HPA016808, 1:500 in PBS containing 3% BSA and 0.3% triton X-100)
Rabbit polyclonal anti-PHF2 (Cell Signaling, D45A2, 1:500 in PBS containing 3% BSA and 0.3% triton X-100)
Mouse monoclonal anti-SREBP1 (BD Biosciences, 557036, 1:500 in PBS containing 3% BSA and 0.3% triton X-100)

For immunofluorescence (secondary antibodies):
Goat anti-Mouse IgG (H+L) Cross-Adsorbed Secondary Antibody, Alexa Fluor™ 568 (Invitrogen, A11004, 1:1,000 in PBS)
Rabbit anti-Goat IgG (H+L) Cross-Adsorbed Secondary Antibody, Alexa Fluor™ 488 (Invitrogen, A11078, 1:1,000 in PBS)
Goat anti-Rabbit IgG (H+L) Highly Cross-Adsorbed Secondary Antibody, Alexa Fluor™ 488 (Invitrogen, A11034, 1:1,000 in PBS)
Goat anti-Rabbit IgG (H+L) Cross-Adsorbed Secondary Antibody, Alexa Fluor™ 568 (Invitrogen, A11011, 1:1,000 in PBS)

For immunohistochemistry (primary antibodies):
Rabbit polyclonal anti-ZDHHC23 (Sigma-Aldrich, HPA016808, 1:200 in PBS containing 2.5% BSA, 0.2% triton X-100, and 10% normal goat serum)
Rabbit polyclonal anti-PHF2 (Cell Signaling, D45A2, 1:200 in PBS containing 2.5% BSA, 0.2% triton X-100, and 10% normal goat serum)
Rabbit polyclonal anti-SREBP1 (Abcam, ab28481, 1:200 in PBS containing 2.5% BSA, 0.2% triton X-100, and 10% normal goat serum)

For immunohistochemistry (secondary antibodies):

	Goat Anti-Rabbit IgG Antibody (H+L), Biotinylated (Vector, BA-1000, 1:200 in PBS containing 2.5% BSA and 0.1% triton X-100)<br>Goat Anti-Mouse IgG Antibody (H+L), Biotinylated (Vector, BA-9200, 1:200 in PBS containing 2.5% BSA and 0.1% triton X-100)
Validation	All antibodies are commercially available and have been tested for species reactivity and validated by the manufacturers and suppliers. The commercial information for the antibodies is as follows:<br><br>Mouse monoclonal anti-SREBP1 (BD Biosciences, 557036): https://www.bdbiosciences.com/ko-kr/products/reagents/western-blotting-and-molecular-reagents/purified-mouse-anti-srebp-1.557036<br>Mouse monoclonal anti-Ubiquitin (Santa Cruz, sc-9133): https://www.scbt.com/p/ub-antibody-fl-76?requestFrom=search<br>Rabbit polyclonal anti-PHF2 (Cell Signaling, D45A2): https://www.cellsignal.com/products/primary-antibodies/phf2-d45a2-rabbit-mab/3497<br>Mouse monoclonal anti-Flag (Sigma-Aldrich, F3165): https://www.sigmaaldrich.com/KR/ko/product/sigma/f3165?gclid=Cj0KCQjwteOaBhDuARIsADBqReialP2D5DLXbdYYwJtZaYmqRAFSeolC_VWELKWxgFFXmFsLYEf6nFEaAtt5EALw_wcB&gclsrc=aw.ds<br>Mouse monoclonal anti-Tubulin (Santa Cruz, sc-166729): https://www.scbt.com/p/beta-tubulin-antibody-f-1?requestFrom=search<br>Rabbit polyclonal anti-ZDHHC23 (Sigma-Aldrich, HPA016808): https://www.sigmaaldrich.com/KR/ko/product/sigma/hpa016808<br>Rabbit polyclonal anti-HA (Santa Cruz, sc-805): https://www.scbt.com/p/ha-probe-antibody-y-11?requestFrom=search<br>Mouse monoclonal anti-Myc (Cell Signaling, 2276s): https://www.cellsignal.com/products/primary-antibodies/myc-tag-9b11-mouse-mab/2276?site-search-type=Products&N=4294956287&Ntt=2276s&fromPage=plp&_requestid=5692836<br>Rabbit polyclonal anti-Myc (Cell Signaling, 2278s): https://www.cellsignal.com/products/primary-antibodies/myc-tag-71d10-rabbit-mab/2278?site-search-type=Products&N=4294956287&Ntt=2278s&fromPage=plp&_requestid=5692866<br>Mouse monoclonal anti-Lamin B (Santa Cruz, sc-374015): https://www.scbt.com/ko/p/lamin-b1-antibody-b-10?requestFrom=search<br>Mouse monoclonal anti-His-tag (MBL, D291-3): https://www.mblbio.com/bio/g/dtl/A/?pcd=D291-3<br>Goat polyclonal anti-PHF2 (Santa Cruz, sc-324199): https://datasheets.scbt.com/sc-324199.pdf<br>Goat anti-mouse igG horseradish peroxidase conjugate (Invitrogen, G21040): https://www.thermofisher.com/antibody/product/Goat-anti-Mouse-IgG-H-L-Cross-Adsorbed-Secondary-Antibody-Polyclonal/G-21040<br>Goat anti rabbit IgG horse radish peroxide conjugate (Invitrogen, G21234): https://www.thermofisher.com/antibody/product/Goat-anti-Rabbit-IgG-H-L-Cross-Adsorbed-Secondary-Antibody-Polyclonal/G-21234<br>Rabbit polyclonal anti-SREBP1 (Abcam, ab28481): https://www.abcam.com/products/primary-antibodies/srebp1-antibody-ab28481.html<br>Goat Anti-Rabbit IgG Antibody (H+L), Biotinylated (Vector, BA-1000): https://vectorlabs.com/products/antibodies/biotinylated-goat-anti-rabbit-igg<br>Goat Anti-Mouse IgG Antibody (H+L), Biotinylated (Vector, BA-9200): https://vectorlabs.com/products/antibodies/biotinylated-goat-anti-mouse-igg<br>Goat anti-Mouse IgG (H+L) Cross-Adsorbed Secondary Antibody, Alexa Fluor™ 568 (Invitrogen, A11004): https://www.thermofisher.com/antibody/product/Goat-anti-Mouse-IgG-H-L-Cross-Adsorbed-Secondary-Antibody-Polyclonal/A-11004<br>Rabbit anti-Goat IgG (H+L) Cross-Adsorbed Secondary Antibody, Alexa Fluor™ 488 (Invitrogen, A11078): https://www.thermofisher.com/antibody/product/Rabbit-anti-Goat-IgG-H-L-Cross-Adsorbed-Secondary-Antibody-Polyclonal/A-11078<br>Goat anti-Rabbit IgG (H+L) Highly Cross-Adsorbed Secondary Antibody, Alexa Fluor™ 488 (Invitrogen, A11034): https://www.thermofisher.com/antibody/product/Goat-anti-Rabbit-IgG-H-L-Highly-Cross-Adsorbed-Secondary-Antibody-Polyclonal/A-11034<br>Goat anti-Rabbit IgG (H+L) Cross-Adsorbed Secondary Antibody, Alexa Fluor™ 568 (Invitrogen, A11011): https://www.thermofisher.com/antibody/product/Goat-anti-Rabbit-IgG-H-L-Cross-Adsorbed-Secondary-Antibody-Polyclonal/A-11011

Eukaryotic cell lines

Policy information about cell lines and Sex and Gender in Research

Cell line source(s)	HepG2 cells (No. 88065) were obtained from the Korea Cell Line Bank (Seoul, Korea). HEK293T (No. CRL-3216), Hep3B (No. HB-8064), PLC/PRF/5 (No. CRL-8024), and SK-HEP-1 (No. HTB-52) cells were obtained from the American Type Culture Collection (ATCC, Manassas, VA).
Authentication	Cell lines were originally authenticated by ATCC and the Korea Cell Line Bank, and were not further authenticated as part of this study.
Mycoplasma contamination	All cell lines tested negative for mycoplasma contamination.
Commonly misidentified lines (See ICLAC register)	No commonly misidentified line was used in this study.

Animals and other research organisms

Policy information about studies involving animals; ARRIVE guidelines recommended for reporting animal research, and Sex and Gender in Research

Laboratory animals	NOD.Cg-PrkdcscidIl2rgtm1Wjl/SzJ mice (NSG, female, 6 weeks old) were obtained from the Jackson Laboratory. Mice were housed in a pathogen-free facility at a temperature of 22-26°C and humidity of 40-60% under a 12-hour light/12-hour dark cycle. Less than 4 mice of the same sex were housed in a cage with free access to food and water.
Wild animals	No wild animals were used in this study.
Reporting on sex	No sex analysis was performed in this study to avoid bias induced by sex.
Field-collected samples	No field-collected samples were used in this study.

Ethics oversight	All experiments were performed following the guidelines of the Seoul National University Institutional Animal Care and Use Committee (approval No. SNU-200108-5-2). Because cancer cells were directly injected into the livers of mice, tumor growth could not be detected without bioluminescence monitoring. Thus, the tumor growth was monitored using the Xenogen IVIS Lumina in vivo imaging technology platform every week for ethics oversight. Mice were also monitored every other day for signs of pain such as hunched posture, highly reduced body weight, and reduced mobility after tumor cells injection. Euthanasia was considered in case of severe signs of pain even before the experiment's endpoint. If euthanasia is needed, mice were subjected to 70% CO_2, and complete euthanasia was confirmed by checking the tail pinch.

Note that full information on the approval of the study protocol must also be provided in the manuscript.
