# Supplementary figures and images for: Palmitoylation-driven PHF2 ubiquitination remodels lipid metabolism through the SREBP1c axis in hepatocellular carcinoma
Source: Nat Commun. 2023 Oct 12;14:6370. doi: 10.1038/s41467-023-42170-0 (PMC10570296; doi:10.1038/s41467-023-42170-0)

# The Source Data of main figures

Fig. 1

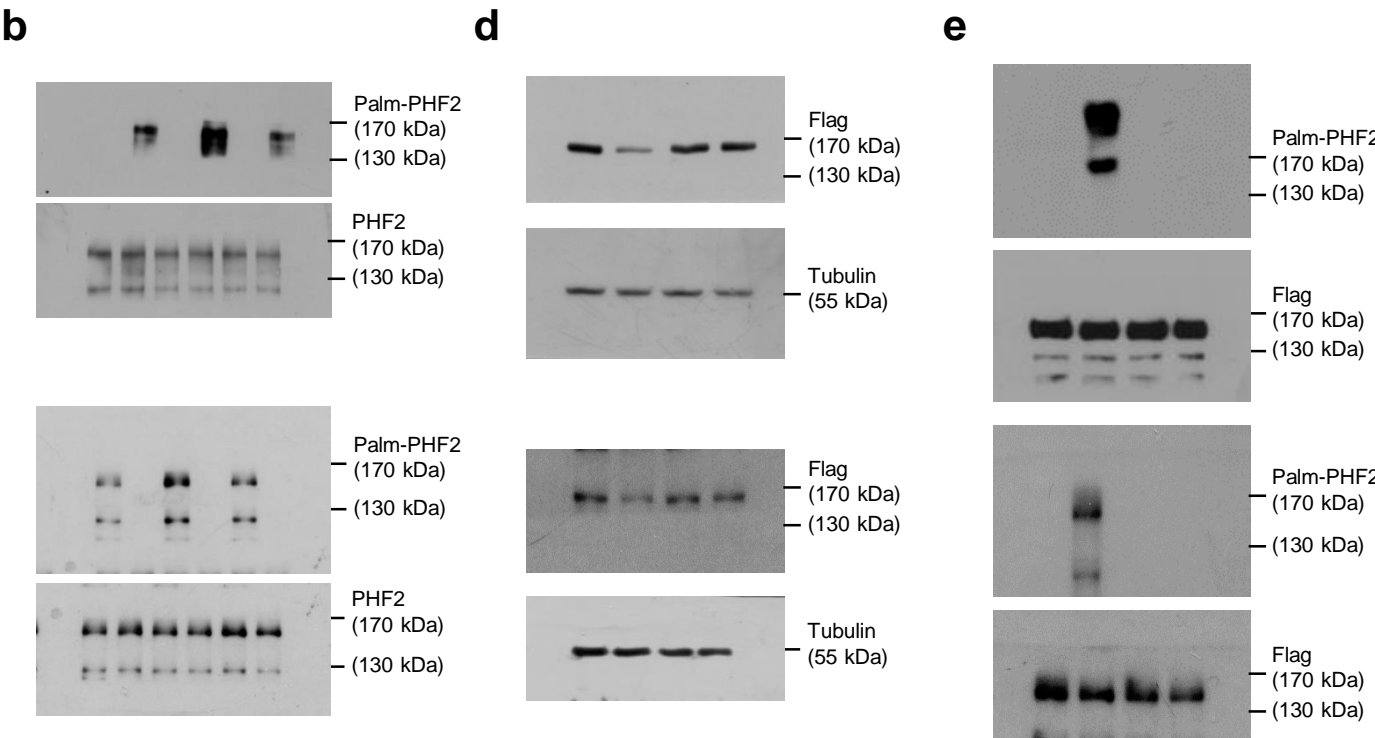

**Fig. 2**

**b**

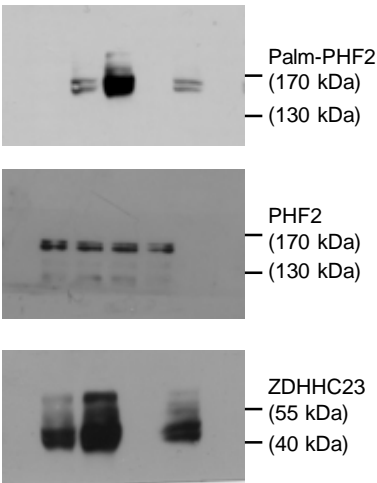

**c**

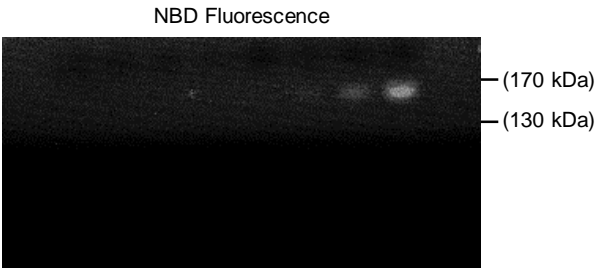

**g**

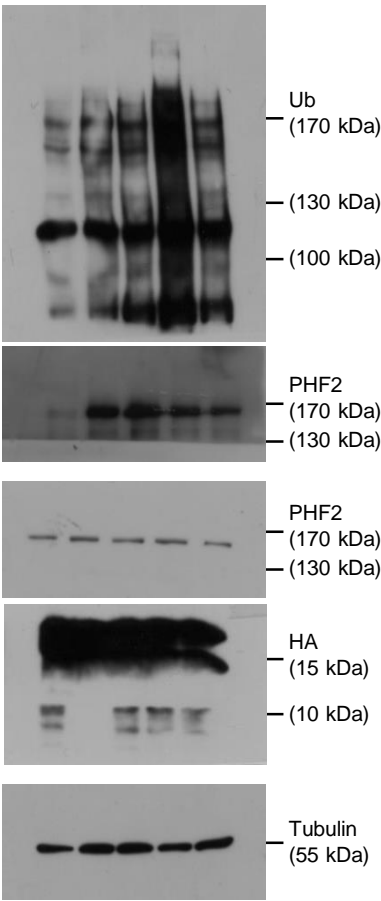

**h**

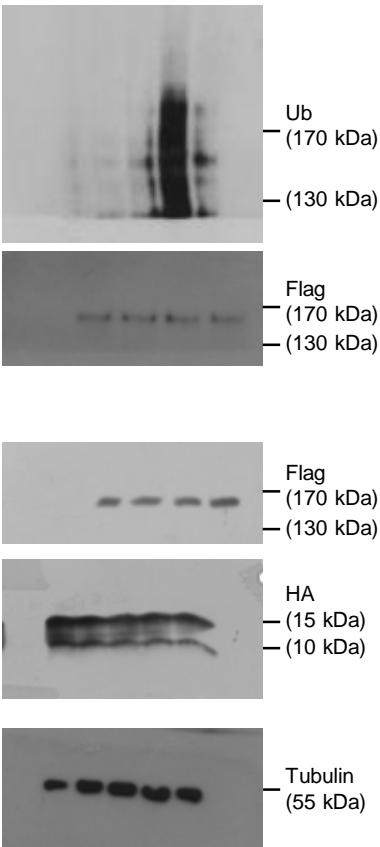

**Fig. 4**

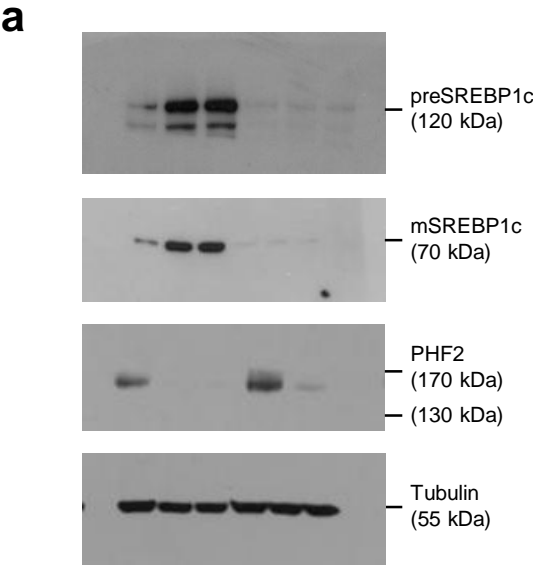

**Fig. 5**

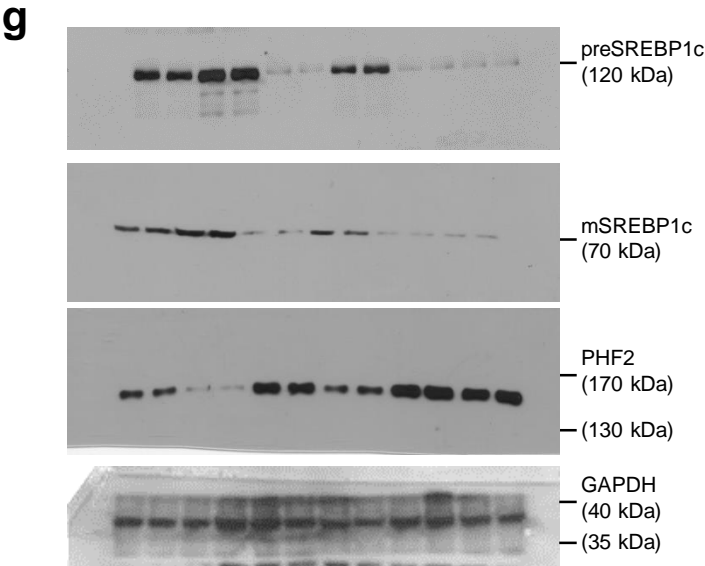

**Fig. 6**

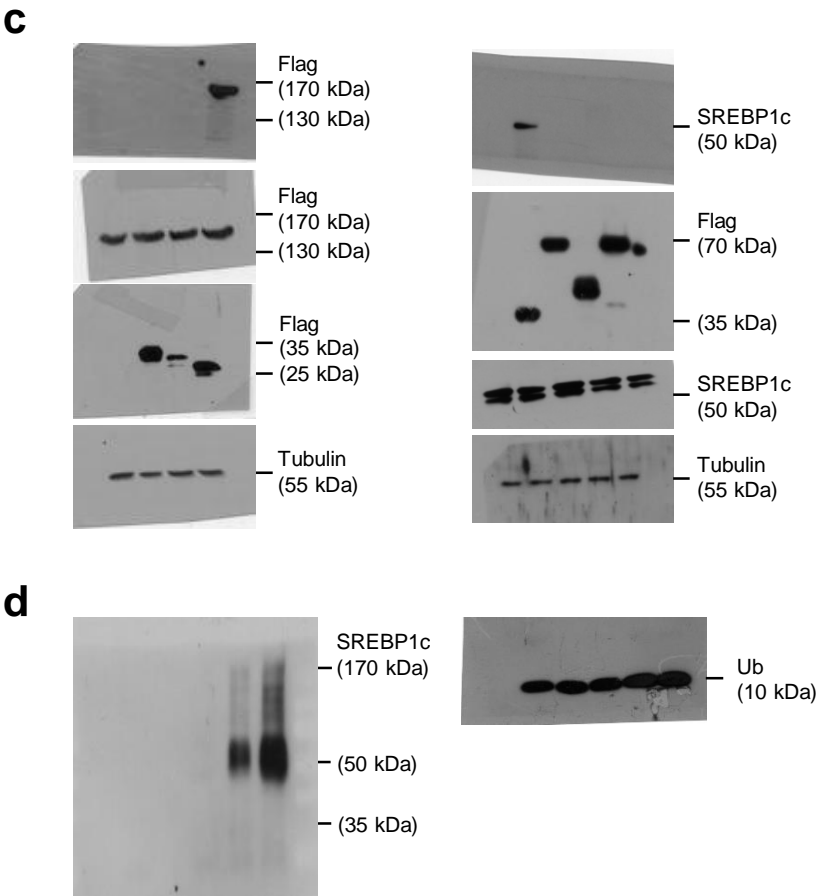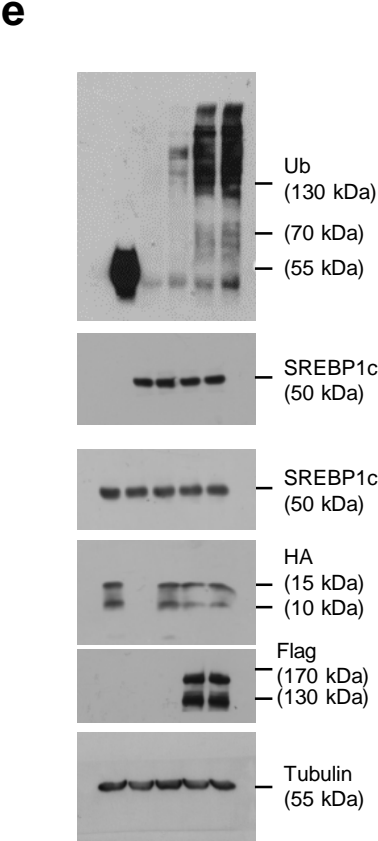

Supplement: Supplementary file 6 — Source Data [file 41467_2023_42170_MOESM6_ESM.pdf]
